# The role of the spleen in red blood cell loss caused by malaria: A mathematical model

**DOI:** 10.1371/journal.pcbi.1013865

**Published:** 2026-01-12

**Authors:** Robert Moss, Saber Dini, Steven Kho, Bridget E. Barber, Pierre A. Buffet, Megha Rajasekhar, David J. Price, Nicholas M. Anstey, Julie A. Simpson

**Affiliations:** 1 Centre for Epidemiology and Biostatistics, Melbourne School of Population and Global Health, University of Melbourne, Melbourne, Victoria, Australia; 2 Global and Tropical Health Division, Menzies School of Health Research and Charles Darwin University, Darwin, Northern Territory, Australia; 3 QIMR Berghofer Medical Research Institute, Brisbane, Queensland, Australia; 4 UMR-S1134, BIGR, Inserm, Université Paris Cité, Institut National de la Transfusion Sanguine and Laboratory of Excellence GR-Ex, Paris, France; 5 Department of Infectious Diseases, The University of Melbourne at the Peter Doherty Institute for Infection and Immunity, Melbourne, Victoria, Australia; 6 Centre for Tropical Medicine and Global Health, Nuffield Department of Clinical Medicine, University of Oxford, Oxford, United Kingdom; Texas Biomedical Research Institute, UNITED STATES OF AMERICA

## Abstract

The human spleen significantly influences red blood cell (RBC) dynamics due to its ability to retain and/or remove RBCs from peripheral blood circulation. This filtering can mediate a range of malaria disease manifestations, depending on the physiological properties of the spleen. Data collected from patients undergoing splenectomy in Papua, Indonesia, revealed that in asymptomatic infections the spleen harboured substantially more infected RBCs than were circulating in the peripheral blood and that the spleen is also congested with uninfected RBCs. We hypothesise that two conditions hold for the spleen to retain such a high proportion of infected and uninfected RBCs: (i) the retention rate of uninfected RBCs is significantly higher than in uninfected patients; and (ii) phagocytosing macrophages cannot clear all of the infected RBCs from the spleen. In this paper, we present a mathematical model of RBC dynamics that includes, for the first time, the spleen as a compartment capable of retaining large numbers of infected and uninfected RBCs in *Plasmodium falciparum* and *P. vivax* infections. By calibrating the model to the Papuan data, we demonstrate that the spleen plays a significant role in removing not only infected RBCs but also uninfected RBCs. Uninfected RBC retention in the spleen, attributable to malaria, is substantially higher than circulating RBC loss due to parasitisation, for infections by both *Plasmodium* species. In chronic infections, the ratio of circulating uninfected RBCs lost to splenic retention per circulating uninfected RBC lost to parasitisation is 17:1 for *P. falciparum* and 82:1 for *P. vivax*. These ratios are larger than previously published estimates for acute clinical infections.

## Author summary

The human spleen is a blood filter, which retains red blood cells (RBCs) that are too rigid to pass through narrow slits and return to the circulation. It has long been considered to protect against *Plasmodium* (malaria) infection, because it removes dead and damaged malaria parasites after antimalarial treatment, and can retain infected RBCs that have reduced deformability. However, a recent study of asymptomatic adults in Papua revealed that the spleen can also act as a reservoir for large quantities of malaria parasites, which suggests that the spleen may be capable of sustaining chronic malaria infection. We present a mathematical model of RBC dynamics in response to malaria, which includes the spleen as an explicit compartment, and use this model to quantify how the removal and retention of uninfected and infected RBCs in the spleen may result in chronic malaria infections with, and without, anaemia. We calibrate this model to the Papuan data to simulate chronic asymptomatic infections, and quantify how much RBC loss is due to (a) retention in the spleen; and (b) infection by malaria parasites. Our findings highlight the spleen as a site in which major RBC retention and parasitisation can be achieved.

## Introduction

The human spleen removes defective and senescent red blood cells (RBCs) from circulation. This removal is primarily facilitated by the narrow inter-endothelial slits through which RBCs must pass in order to return to the peripheral circulation [1]. A RBC must be sufficiently deformable in order to cross these inter-endothelial slits and avoid biomechanical trapping and splenic clearance.

The spleen has long been considered a protective organ against *Plasmodium* infection, by removing dead/damaged intraerythrocytic parasites from the blood circulation after antimalarial treatment [2]. *Plasmodium falciparum* (Pf)-infected RBCs (iRBCs) have reduced deformability, and so are more likely to be retained in the spleen and subject to splenic clearance [3]. Moreover, in patients treated with artemisinins, “pitting” (a splenic process where parasite remnants are removed from iRBCs while crossing inter-endothelial slits) helps to recover large numbers of RBCs [4]. Consequently, studies have repeatedly shown that patients who have undergone a splenectomy (removal of the spleen) have higher risk of clinical malaria and are more prone to adverse outcomes [5–10].

Despite these protective benefits conferred by the spleen, Kho et al. [11] recently showed that the spleen can also act as a reservoir for large quantities of viable malaria parasites. The authors studied 15 asymptomatic patients who underwent splenectomy in Papua, and found that asexual parasitaemia in the spleen of these patients was, on average, 289-fold and 3590-fold higher than in peripheral circulation, for patients infected with Pf and *Plasmodium vivax* (Pv), respectively. This suggests a new paradigm: that the spleen can harbour a large parasite biomass and sustain chronic malaria infection. The study also revealed that in the majority of these asymptomatic individuals, the size of the spleens were enlarged (splenomegaly), and was later described to be mostly a result of congestion with uninfected RBCs (uRBCs) [12]. Similarly, Woodford et al. [13] observed an increase in splenic volume during the early stages of human volunteer infection studies with Pv. These studies [11–13] and others [14,15] highlight the importance of the spleen as a compartment for hidden malaria parasites, with major implications for our understanding of the biology, pathology, and treatment of malaria.

In endemic regions, asymptomatic and submicroscopic *Plasmodium* infections are associated with elevated risks of anaemia [16,17] and congestion of the spleen has been identified as a major cause of apparent uRBC loss from circulation in asymptomatic infections [12]. In acute clinical malaria, there is clear evidence that a large proportion of RBC loss is also attributable to uRBCs. Jakeman et al, [18] fitted a mathematical model to historical data (albeit without inclusion of a splenic compartment) from neurosyphilis patients undergoing malaria therapy and inferred that parasitisation (invasion of uRBCs by merozoites) only caused around 10% of observed RBC loss. Analyses of human data from epidemiological studies and experimentally-induced blood-stage infections have also found that parasitisation accounts for only a small fraction of overall RBC loss [19–21].

Reduced uRBC deformability, decoration of uninfected RBC with *P. falciparum* proteins [22,23], and immune effects on bystander uRBC have been reported in patients with falciparum and vivax malaria [24–30], and increased splenic filtration stringency has also been proposed [12,31,32], all of which may contribute to increased splenic trapping of uRBCs and apparent loss in circulation. In an empirical rat malaria model, Safeukui et al. [33] found that increasing parasite removal in the spleen also increased uninfected-erythrocyte removal in the spleen. Furthermore, multiple studies in clinical malaria show that splenomegaly is associated with anaemia, including severe malarial anaemia [15,34–36]. Thus, like in asymptomatic infections, the spleen may play a major direct role in the development of anaemia in clinical disease.

In this study, we present a within-host mathematical model that characterises RBC dynamics in response to malaria, and includes the spleen as an explicit compartment that interacts with the peripheral circulation. The model accounts for RBC age-dependence in various regulatory processes that govern RBC dynamics. We use this model to quantify how splenic processes, such as the removal and retention of uRBCs and iRBCs from the circulation, and RBC phagocytosis by macrophages, may result in chronic malaria infections with, and without, anaemia. In particular, we identify parameter values for which the model reproduces the observed numbers of uRBCs and iRBCs in the spleen and circulation of asymptomatic Pf- and Pv-infected individuals with a large hidden splenic biomass [11]. By calibrating the model to these cross-sectional splenectomy data, we are able to characterise chronic asymptomatic infections, but stress that the model results do not accurately describe the acute infection phase (i.e., the transition from initial blood infection to chronic asymptomatic infection). We also use this model to quantify the ratio of circulating uRBCs lost to splenic retention per circulating uRBC lost to parasitisation, and report how our values differ from previous estimates without inclusion of a splenic compartment. Our findings highlight the spleen as a site in which major RBC retention and parasitisation can be achieved.

## Materials and methods

### Ethics statement

We obtained ethical approval from the Human Research Ethics Committee of Northern Territory Health and Menzies School of Health Research for (a) the uRBC and iRBC counts in 15 asymptomatic Papuan adults (HREC-2010-1397); and (b) the RBC counts and blood volumes from 708 healthy Papuan residents (HREC-2010-1434). Written informed consent was obtained from each participant, or from their parent/guardian.

### Patient data

Model outputs were compared to published cross-sectional uRBC and iRBC counts in the circulation and spleen of 15 asymptomatic adults in Papua, Indonesia (9 Pf infections, 6 Pv infections) who underwent splenectomy [11]. Briefly, splenectomy in this cohort was primarily due to splenic injury from trauma (8 Pf, 5 Pv); the remaining two patients (1 Pf, 1 Pv) underwent elective splenectomy due to clinically significant splenomegaly. The uRBC and iRBC counts for each patient are listed in Table 1, determined in peripheral blood by automated counts and Giemsa-based microscopy, respectively, and in the spleen by Giemsa-based histology, as previously described [12,37]. Infecting Plasmodium species was confirmed by PCR [37]. Notably, patients 5 (Pf) and 9 (Pf) had no iRBCs detected in their circulation, patient 8 (Pf) had a much larger circulating iRBC count than other patients ($2.15\;\times \;10^{10}$) and patient 9 (Pf) had a much larger splenic uRBC count than other patients ($5.27\times 10^{12}$, 32% of their total uRBC count), but they otherwise had RBC counts consistent with the other patients.

**Table 1:** RBC counts for each asymptomatic adult [11].

ID	Species	iRBC circ	iRBC spleen	uRBC circ	uRBC spleen
1	Pf	$3.15\times 10^{9}$	$4.27\times 10^{10}$	$1.24\times 10^{13}$	$4.95\times 10^{11}$
2	Pf	$3.42\times 10^{8}$	$8.56\times 10^{9}$	$1.28\times 10^{13}$	$3.81\times 10^{11}$
3	Pf	$3.97\times 10^{7}$	$9.56\times 10^{9}$	$1.49\times 10^{13}$	$1.67\times 10^{12}$
4	Pf	$3.99\times 10^{9}$	$1.99\times 10^{9}$	$2.23\times 10^{13}$	$3.28\times 10^{11}$
5	Pf	$0.00\times 10^{0}$	$2.18\times 10^{9}$	$1.53\times 10^{13}$	$9.46\times 10^{10}$
6	Pf	$4.28\times 10^{8}$	$4.69\times 10^{9}$	$9.10\times 10^{12}$	$1.12\times 10^{12}$
7	Pf	$3.55\times 10^{8}$	$4.36\times 10^{9}$	$1.57\times 10^{13}$	$1.78\times 10^{11}$
8	Pf	$2.15\times 10^{10}$	$1.03\times 10^{10}$	$1.35\times 10^{13}$	$3.59\times 10^{11}$
9	Pf	$0.00\times 10^{0}$	$2.89\times 10^{10}$	$1.12\times 10^{13}$	$5.27\times 10^{12}$
10	Pv	$2.65\times 10^{8}$	$2.57\times 10^{10}$	$2.28\times 10^{13}$	$6.19\times 10^{11}$
11	Pv	$2.97\times 10^{7}$	$2.80\times 10^{9}$	$1.22\times 10^{13}$	$3.41\times 10^{11}$
12	Pv	$4.01\times 10^{7}$	$2.68\times 10^{9}$	$1.12\times 10^{13}$	$2.48\times 10^{11}$
13	Pv	$3.03\times 10^{7}$	$1.61\times 10^{10}$	$1.13\times 10^{13}$	$1.87\times 10^{12}$
14	Pv	$3.97\times 10^{7}$	$6.40\times 10^{8}$	$1.95\times 10^{13}$	$7.61\times 10^{10}$
15	Pv	$3.13\times 10^{7}$	$2.65\times 10^{8}$	$1.07\times 10^{13}$	$4.91\times 10^{10}$

To define the RBC count at homeostasis, in the absence of malaria, we used published RBC counts ($10^{6}/\mu l$) and blood volumes (L) from 708 healthy Papuan residents (negative results for both *Plasmodium* microscopy and PCR), who reported no fever within the preceding 24 hours, as collected in a cross-sectional household survey conducted in southern Papua, Indonesia [16]. The RBC count at homeostasis was set to $1.75\;\times \;10^{13}$ cells, the median total count in these Papuan residents. The mean haemoglobin was 12.8 g/dL and we converted between haemoglobin (g/dL) and circulating uRBC counts under the assumption that 12.8 g/dL of haemoglobin corresponded to $1.75\times 10^{13}$ uRBCs.

Although all biological measurements contain inherent uncertainty, the patient data, based on Giemsa-based blood microscopy and spleen histology, was obtained from results validated by multiple independent methods, conservative assumptions, and validation by multiple expert microscopists [11,37]. Briefly, in the original study describing the patient data, parasite quantification of Giemsa-based microscopy was validated by a second microscopist in 30% of patients, and a third external microscopist qualitatively reviewed 20%. Plasmodium species-specific PCR provided independent molecular confirmation of infecting species. Immunohistochemistry of macrophages (CD68), as well as electron microscopy, provided verification that parasites were intact and non-phagocytosed. Staining with PvAMA1 provided species and stage-specific confirmation of Giemsa-based results. Parasite morphology and quantification was further validated through *ex vivo* spleen perfusion experiments with known parasite cultures, which were also used for internal concordance validation between microscopists. Several features of the study also suggest a potential minimal impact of any error on the main patient data, including the magnitude of splenic enrichment and the relative consistency across patients, the use of conservative assumptions (10 parasite/uL assigned to submicroscopic PCR-positive peripheral infections), and exclusion of splenic parasites with indeterminate stage or phagocytosis status from parasitemia and biomass calculations, both of which biases against finding larger splenic biomass. Overall, the log-scale differences between splenic and peripheral parasitemia make it highly unlikely that any measurement error could substantially alter the conclusions of the original study.

### Mathematical model

**Fig 1:**
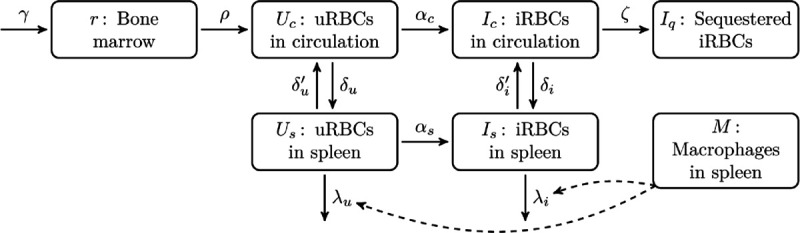
A schematic of the model structure. This shows the various compartments in which red blood cells (RBCs) may reside, and the permitted movements of RBCs between these compartments. See Table 2 for descriptions of each cell population, and Table 3 for descriptions of each rate parameter. In brief, normoblasts are produced in the bone marrow and mature into reticulocytes, which are then released from the bone marrow into the circulation, from where they may pass through the spleen and either be retained or released back into the circulation. Uninfected RBCs (uRBCs) can be parasitised in the circulation and in the spleen. Infected RBCs (iRBCs) in the circulation can become sequestered in the microvasculature (Pf only). Phagocytosis of uRBCs and iRBCs in the spleen is driven by the splenic macrophage population (indicated by dashed lines).

**Fig 2:**
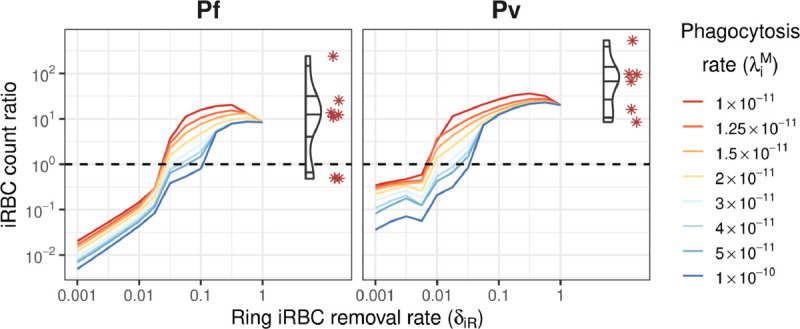
The steady-state ratio of iRBCs retained in the spleen (absolute counts, *I*_*s*_) to iRBCs in the circulation (absolute counts, *I*_*c*_). Ratios are shown for a range of values for ring iRBC removal from circulation ($\delta_{iR}$) and iRBC phagocytosis in the spleen ($\lambda_{i}^{M}$), shown as coloured lines. Corresponding data in the splenectomy patients (Pf: n = 9; Pv: n = 6) are shown on the right of each plot for comparison (violin plots for distribution and red asterisks for observed data points).

**Fig 3:**
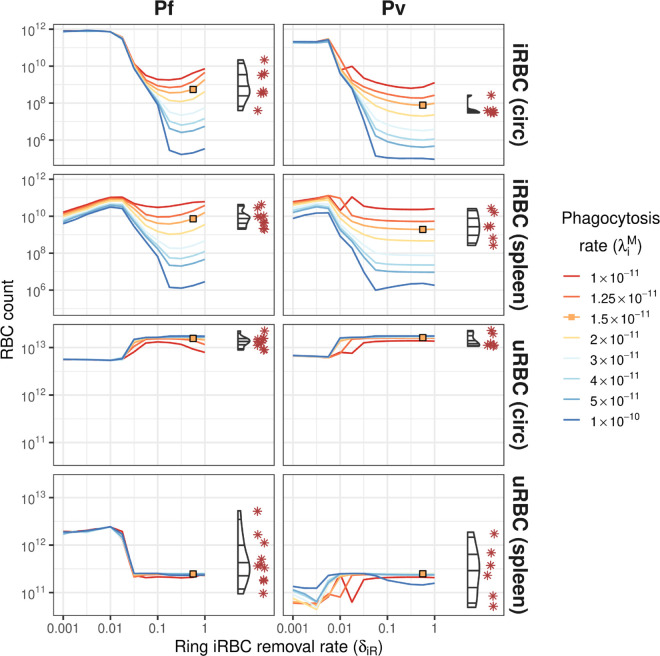
Steady-state uRBC and iRBC absolute counts in the circulation and spleen. RBC counts are shown for a range of values for ring iRBC removal from circulation ($\delta_{iR}$) and iRBC phagocytosis in the spleen ($\lambda_{i}^{M}$), shown as coloured lines. Corresponding data in the splenectomised patients (Pf: n=9; Pv: n=6) are shown on the right of each plot for comparison (violin plots for distribution and red asterisks for observed data points). Orange squares indicate good agreement between the model and the data.

The model structure is outlined in Fig 1, the model cell populations are described in Fig 2, and rate parameters are described in Fig 3. The model is implemented in the R programming language [38] as a set of difference equations that describe the dynamics of the age-structured RBC populations over a series of one-hour time steps. We present here an overview of the processes that govern RBC dynamics in the model, and provide all of the model equations. We define the net movement of uRBCs and iRBCs between model compartments with the notation $U_{x\to y}$ and $I_{x\to y}$, respectively, where *x* and *y* may refer to the circulation (*c*), the spleen (*s*), or the microvasculature (*q*). We also use this same notation to define the quantities of uRBCs and iRBCs that remain within a model compartment *x*: $U_{x\to x}$ and $I_{x\to x}$. Similarly, we define the net release of reticulocytes into the circulation with the notation $r_{\to c}$. For further details, including definitions and baseline values for each model parameter, see the supplementary Model Description (S1 File).

**Table 2:** The cell populations in the model, with respect to age *a* and time *t* where appropriate.

Symbol	Compartment	Cell type	Lifespan (days)
r(a,t)	Bone marrow	Normoblasts and reticulocytes	3.5
*U*_*c*_(*a*,*t*)	Circulation	Uninfected RBCs	120
*I*_*c*_(*a*,*t*)	Circulation	Infected RBCs	2
*I*_*q*_(*a*,*t*)	Microvasculature	Infected RBCs	2
*U*_*s*_(*a*,*t*)	Spleen	Uninfected RBCs	120
*I*_*s*_(*a*,*t*)	Spleen	Infected RBCs	2
M(t)	Spleen	Red-pulp macrophages	—

**Table 3:** The rates that govern the movements of RBCs between the model compartments (units: RBCs/hour).

Symbol	Process
*γ*	Production rate of normoblasts in the bone marrow.
*ρ*	Release rate of reticulocytes from the bone marrow into the circulation.
$\delta_{u}$	Removal rate of uRBCs from the circulation into the spleen.
$\delta_{u}^{\prime }$	Return rate of uRBCs from the spleen into the circulation.
$\lambda_{u}$	Phagocytosis rate of uRBCs in the spleen.
$\lambda_{u}^{M}$	uRBC phagocytosis rate per splenic macrophage.
$\delta_{i}$	Removal rate of iRBCs from the circulation into the spleen.
$\delta_{i}^{\prime }$	Return rate of iRBCs from the spleen into the circulation.
$\lambda_{i}$	Phagocytosis rate of iRBCs in the spleen.
$\lambda_{i}^{M}$	iRBC phagocytosis rate per splenic macrophage.
$\alpha_{c}$	Invasion rate of released merozoites in the circulation.
$\alpha_{s}$	Invasion rate of released merozoites in the spleen.
ζ	Sequestration rate of iRBCs into the microvasculature.

#### RBC production and release into the circulation.

Erythropoiesis was modelled by the hourly production of *γ* normoblasts in the bone marrow; other organs were assumed to make negligible contributions. We selected the baseline value of *γ* such that, in the absence of infection, the circulating RBC population remains constant at a steady-state value *U*_*ss*_; see Eq (1). Note that the precise value of *γ* depends on the rate at which uRBCs are removed by the spleen.

$$U_{ss}\approx \sum_{a}U_{c}(a):r(1)=\gamma$$ (1)

We assumed that erythropoiesis increases in response to decreases in the circulating RBC population [39], with a half-maximal increase when the circulating RBC population drops to a fraction $U_{c}^{l}$ of the baseline value *U*_*ss*_. We assumed a maximum 10-fold increase in RBC production $f_{max}$ based on findings from a previous modelling study [40].

$$U_{l}=U_{c}^{l}\cdot U_{ss}$$ (2)

$$eryth(t)=\gamma \cdot \left(1+\frac{f_{max}-1}{2}\cdot \left[1-\tanh\left(e_{sl}\cdot \frac{{𝐔}_{𝐜}(t)-U_{l}}{U_{l}}\right)\right]\right)$$ (3)

Under normal circumstances, erythroblasts in the bone marrow mature into normoblasts and then reticulocytes over a period of 3.5 days, before being released into the circulation and maturing into normocytes after 1 day in the peripheral blood [39,41]. With increasing anaemia, the maturation time *t*_*R*_ in the bone marrow shortens to a minimum of $T_{R}^{\min}=1$ day, with a corresponding increase in the time taken to mature into normocytes in the peripheral blood [39,41]. We calibrated the maturation time *t*_*R*_ as a function of the circulating RBC population based on manual reticulocyte counts and haematocrits data [41,42].

$$t_{r}=\left\{ \begin{array}{cc} T_{R} & \text{when }{𝐔}_{𝐜}(t)>U_{ss}\\ \begin{array}{c} T_{R}^{\min}+(T_{R}-T_{R}^{\min})\times \\ {\left(1+\exp\left[-\rho_{s}\cdot \left(\frac{{𝐔}_{𝐜}(t)}{U_{ss}}-\rho_{i}\right)\right]\right)}^{-1} \end{array} & \text{when }{𝐔}_{𝐜}(t)\leq U_{ss} \end{array}\right.$$ (4)

$${𝐔}_{𝐜}(t)=\sum_{a}U_{c}(a,t)$$ (5)

In the model, reticulocytes are initially released very slowly into circulation (hourly release probability of 0.001). Upon reaching the release age *t*_*R*_, which depends on the circulating RBC population, reticulocytes are rapidly released into the circulation (hourly release probability >0.9999).

$$\rho (a,t)=\left\{ \begin{array}{cc} 10\cdot \min(20,\exp[\kappa \cdot (U_{ss}-{𝐔}_{𝐜}(t))]) & \text{for }a\geq t_{r}\\ \rho_{o}\cdot \min(20,\exp[\kappa \cdot (U_{ss}-{𝐔}_{𝐜}(t))]) & \text{for }a<t_{r} \end{array}\right.$$ (6)

$$r(a,t)=\left\{ \begin{array}{cc} eryth(t) & \text{for }a=1\\ r(a-1,t-1)\cdot \exp[-\rho ] & \text{for }1<a\leq T_{R} \end{array}\right.$$ (7)

$$r_{\to c}(a,t)=r(a-1,t-1)\cdot (1-\exp[-\rho ])$$ (8)

#### Uninfected RBC circulation and retention in the spleen.

In the model, uRBC retention in the spleen arises from the combination of (a) the removal rate from circulation $\delta_{u}$; and (b) the return rate from the spleen $\delta_{u}^{\prime }$. Note that any desired level of uRBC retention can be achieved with arbitrarily many combinations of removal and return rates, due to their inverse relationship (e.g., a high removal rate can be countered by a high return rate).

We set the uRBC return rate from the spleen into the circulation ($\delta_{u}^{\prime }$) to zero for all uRBCs *except* maturing reticulocytes (3–4.5 days old) with a log-normal distribution *F*_*X*_, and characterised uRBC retention in the spleen by the removal rate $\delta_{u}$.

$$\delta_{u}^{\prime }(a,t)=mag\cdot F_{X}(a)$$ (9)

$$\log(X)∼𝒩(\mu =24\cdot \mu_{U},\sigma =24\cdot \sigma_{U})$$ (10)

$$U_{s\to c}(a,t)=U_{s}(a-1,t-1)\cdot \exp[-\lambda_{u}(a,t)]\cdot (1-\exp[-\delta_{u}^{\prime }(a)])$$ (11)

We assumed the uRBC removal rate rate is high (≈0.08) for very mature rigid RBCs (110–120 days old) and for immature reticulocytes (<4.5 days old), which have reduced deformability and increased cytoadherence capacity [43] and will remain in the splenic red-pulp until they mature and are returned to the circulation [37]. RBCs that are 4.5–110 days old are removed from the circulation into the spleen at an extremely low rate ($\approx 2\;\times \;10^{-4}$). We achieve this by combining two functional forms for $\delta_{u}$: an exponential *f*_*I*_ that decreases rapidly for the immature reticulocytes and a sigmoid *f*_*M*_ with a steep slope and late inflection point for the very mature RBCs.

$$\delta_{u}(a,t)=F_{U}(t)\cdot \left[f_{I}(a,t)+f_{M}(a,t)\right]$$ (12)

$$f_{I}(a,t)=\delta_{U}^{A}\exp[-k_{1}\cdot (a-1)]$$ (13)

$$f_{M}(a,t)=\delta_{U}^{\min}+\frac{(a-1{)}^{\delta_{U}^{g}}}{(a-1{)}^{\delta_{U}^{g}}+(\delta_{U}^{c50}{)}^{\delta_{U}^{g}}}$$ (14)

$$k_{1}=-\frac{\log\left(\frac{\delta_{U}^{\min}}{\delta_{U}^{A}}\right)}{23\cdot 7}$$ (15)

$$U_{c\to s}(a,t)=U_{c}(a-1,t-1)\cdot (1-\exp[-\delta_{u}(a,t)])$$ (16)

Finally, we assumed that circulating iRBCs stimulate an increase *F*_*U*_ in splenic retention of uRBCs, either from increased splenic filtration stringency [12,31,32] or reduced circulating uRBC deformability [24,26,27,30], with a maximum increase of $k_{\nu }^{U}=100\%$ (i.e., a doubling of the removal rate $\delta_{u}$) when the circulating iRBC population *I*_*c*_ exceeds 10^8^ cells.

$$F_{U}(t)=1+k_{\nu }^{U}\cdot \frac{I_{c}(t-1{)}^{g_{d}^{U}}}{I_{c}(t-1{)}^{g_{d}^{U}}+{\left(\delta_{50}^{U}\cdot \left[I_{c}(t-1)+{𝐔}_{𝐜}(t-1)\right]\right)}^{g_{d}^{U}}}$$ (17)

$$I_{c}(t)=\sum_{a}I_{c}(a,t)$$ (18)

#### Infected RBC dynamics.

Infected RBC retention in the spleen arises from the combination of (a) the removal rate from circulation $\delta_{i}$; and (b) the return rate from the spleen $\delta_{i}^{\prime }$. As per uRBC retention, there is an inverse relationship between these two rates. Safeukui et al. [14] conducted *ex vivo* experiments that showed 11% of Pf rings and 20% of Pf schizonts are retained in the spleen in every passage of the iRBCs through the spleen. Accordingly, we assumed that the removal rate increases with maturity (hourly removal probabilities of 0.43 for rings and 0.67 for schizonts), while the return rate decreases with maturity (hourly return probabilities of 0.017 for rings and 0.011 for schizonts), and characterised these age-specific removal and return rates using sigmoids with inflections at $\delta_{I}^{c50}=26$ hours.

$$\delta_{i}(a,t)=F_{I}(t)\cdot \left[\delta_{iR}+\left(\delta_{iS}-\delta_{iR}\right)\cdot \frac{(a-1{)}^{\delta_{I}^{sl}}}{(a-1{)}^{\delta_{I}^{sl}}+(\delta_{I}^{c50}{)}^{\delta_{I}^{sl}}}\right]$$ (19)

$$\delta_{iS}=\delta_{iR}\cdot k_{iS}$$ (20)

$$I_{c\to s}(a,t)=I_{c}(a-1,t-1)\cdot (1-\exp[-\delta_{i}(a,t)])$$ (21)

$$\delta_{i}^{\prime }(a)=\delta_{iR}^{\prime }+\left(\delta_{iS}^{\prime }-\delta_{iR}^{\prime }\right)\cdot \frac{(a-1{)}^{\delta_{I}^{sl}}}{(a-1{)}^{\delta_{I}^{sl}}+(\delta_{I}^{c50}{)}^{\delta_{I}^{sl}}}$$ (22)

$$\delta_{iR}^{\prime }=\delta_{iR}\cdot k_{iR}$$ (23)

$$\delta_{iS}^{\prime }=\delta_{iS}\cdot k_{iS}$$ (24)

$$I_{s\to c}(a,t)=I_{s}(a-1,t-1)\cdot \exp[-\lambda_{i}(a,t)]\cdot (1-\exp[-\delta_{i}^{\prime }(a,t)])$$ (25)

We assumed that circulating iRBCs stimulate an increase *F*_*I*_ in splenic retention of iRBCs, with an assumed maximum increase of $k_{\nu }^{I}=300\%$ (i.e., a four-fold increase) when the circulating iRBC population *I*_*c*_ exceeds 10^11^ cells.

$$F_{I}(t)=1+k_{\nu }^{I}\cdot \frac{I_{c}(t-1{)}^{g_{d}^{U}}}{I_{c}(t-1{)}^{g_{d}^{U}}+{\left(\delta_{50}^{I}\cdot \left[I_{c}(t-1)+{𝐔}_{𝐜}(t-1)\right]\right)}^{g_{d}^{U}}}$$ (26)

For Pf infections, circulating iRBCs are also sequestered into the microvasculature at an age-dependent rate ζ. This rate is very low for Pf rings, with an hourly sequestration probability of ≪0.01, and increases with maturity up to a maximum hourly sequestration probability of 0.99 for schizonts.

$$\zeta (a)=-\log(1-0.99)\cdot \frac{a^{\zeta_{sl}}}{a^{\zeta_{sl}}+\zeta_{50}^{\zeta_{sl}}}$$ (27)

$$I_{c\to q}(a,t)=I_{c}(a-1,t-1)\cdot (1-\exp[-\zeta (a)])$$ (28)

$$I_{q}(a,t)=I_{q}(a-1,t-1)+I_{c\to q}(a,t)$$ (29)

#### Infection of RBCs.

Merozoites are released when iRBCs in the circulation and in the spleen rupture ($I_{c}^{\nabla }$ and $I_{s}^{\nabla }$, respectively), and that this occurs 48 hours after parasitisation ($T_{irbc}$).

$$I_{c}^{\nabla }=I_{c}(T_{irbc},t-1)\cdot \exp[-\delta_{i}]\cdot \exp[-\zeta ]$$ (30)

$$I_{s}^{\nabla }=I_{s}(T_{irbc},t-1)\cdot \exp[-\delta_{i}^{\prime }]\cdot \exp[-\lambda_{i}]$$ (31)

We assume that, in the spleen and circulation, each ruptured iRBC infects 8 uRBCs (a parasite multiplication factor of 8) with an age-specific merozoite preference β(a). We have assumed a relatively low multiplication factor because of the sustained immune response in these asymptomatic chronic infections.

$$\alpha_{c}(a,t)=PMF\cdot \frac{\beta (a)\cdot U_{c}(a,t-1)}{\sum_{a^{\prime }}\left[\beta (a^{\prime })\cdot U_{c}(a^{\prime },t-1)\right]}$$ (32)

$$\alpha_{s}(a,t)=PMF\cdot \frac{\beta (a)\cdot U_{s}(a,t-1)}{\sum_{a^{\prime }}\left[\beta (a^{\prime })\cdot U_{s}(a^{\prime },t-1)\right]}$$ (33)

Merozoites released into the circulation infect uRBCs in the circulation [$\nabla_{c\to c}$; Eq (34)], while we assume that merozoites released into the spleen *primarily* infect uRBCs in the spleen [$\nabla_{s\to s}$; Eq (35)], but allow a small proportion (ω=10%) of these merozoites (those from rupturing schizonts in the splenic perifollicular zones, or those escaping through inter-endothelial slits [37]) to infect uRBCs in the circulation [$\nabla_{s\to c}$; Eq (36)]. We assume that merozoites released by iRBCs sequestered in the microvasculature infect uRBCs in the circulation; see Eq (34).

$$\nabla_{c\to c}(a,t)=\alpha_{c}(a,t)\cdot [I_{c}^{\nabla }+I_{q}(T_{irbc},t-1)]$$ (34)

$$\nabla_{s\to s}(a,t)=\alpha_{s}(a,t)\cdot (1-\omega )\cdot I_{s}^{\nabla }$$ (35)

$$\nabla_{s\to c}(a,t)=\alpha_{c}(a,t)\cdot \omega \cdot I_{s}^{\nabla }$$ (36)

This results in $\nabla_{c}$ newly infected RBCs in the circulation and $\nabla_{s}$ newly infected RBCs in the spleen.

$$\nabla_{c}(t)=\sum_{a}\nabla_{c\to c}(a,t)+\sum_{a}\nabla_{s\to c}(a,t)$$ (37)

$$\nabla_{s}(t)=\sum_{a}\nabla_{s\to s}(a,t)$$ (38)

Pf parasites invade RBCs of all ages, with some preference for younger RBCs [44,45], which we characterised using a sigmoid with an inflection at $a_{\beta }^{50}=80$ days:

$$\beta^{\prime }(a)=\frac{a_{\beta }^{50}\cdot \exp\left[-5\times 10^{-4}\cdot (a-1)\right]}{(a-1{)}^{{sl}_{\beta }^{\text{Pf}}}+(a_{\beta }^{50}{)}^{{sl}_{\beta }^{\text{Pf}}}}$$ (39)

In contrast, Pv parasites only invade reticulocytes (RBCs with age $a\leq T_{M}=4.5\text{ days}$) and prefer immature reticulocytes [46]. We characterise this with a linearly decreasing function of RBC age, with a slope parameter ${sl}_{\beta }^{\text{Pv}}$.

$$\beta^{\prime }(a)=\left\{ \begin{array}{cc} 1-(a-1)\cdot {\left[{sl}_{\beta }^{\text{Pv}}\right]}^{-1} & \text{if }a\leq T_{M}\\ 0 & \text{otherwise} \end{array}\right.$$ (40)

We normalise these age-specific preferences so that they sum to 1:

$$\beta (a)=\frac{\beta^{\prime }(a)}{\sum_{A}\beta^{\prime }(A)}$$ (41)

#### RBC destruction in the spleen.

Each individual macrophage destroys uRBCs at a rate $\lambda_{u}^{M}$ and destroys iRBCs at a rate $\lambda_{i}^{M}$, and we assume that $\lambda_{u}^{M}\gg \lambda_{i}^{M}$ from the larger number of aged uRBCs compared to iRBCs that are retained and subject to phagocytosis at interendothelial slits in the spleen.

$$\lambda_{u}(a,t)=\left\{ \begin{array}{cc} 0 & \text{for }a\leq T_{M}\\ \lambda_{u}^{M}\cdot M_{u}(t-1) & \text{for }a>T_{M} \end{array}\right.$$ (42)

$$\lambda_{i}(a,t)=\lambda_{i}^{M}\cdot M_{i}(t-1)$$ (43)

$$M_{u}(t)=M(t)\cdot \frac{U_{s}(t-1{)}^{\gamma_{M}}}{U_{s}(t-1{)}^{\gamma_{M}}+I_{s}(t-1{)}^{\gamma_{M}}}$$ (44)

$$M_{i}(t)=M(t)\cdot \frac{I_{s}(t-1{)}^{\gamma_{M}}}{U_{s}(t-1{)}^{\gamma_{M}}+I_{s}(t-1{)}^{\gamma_{M}}}$$ (45)

The net phagocytosis rates $\lambda_{u}$ and $\lambda_{i}$ depend on the splenic macrophage population in the red-pulp *M*(*t*). At homeostasis we set this population to ($M_{0}=1.2\;\times \;10^{9}$), as calculated from published healthy controls [47]. The macrophage population expands during infection in proportion to the quantity of RBCs retained in the spleen, relative to the quantity *U*_*r*,*ss*_ retained at homeostasis, based on higher red-pulp macrophage counts observed in asymptomatic infections [12].

$$M(t)=k_{M}\left[b_{M}\left(U_{s}(t-1)+I_{s}(t-1)\right)-M(t-1)\right]+M(t-1)$$ (46)

$$U_{s}(t)=\sum_{a}U_{s}(a,t)$$ (47)

$$I_{s}(t)=\sum_{a}I_{s}(a,t)$$ (48)

$$b_{M}=\frac{M_{0}}{U_{r,ss}}$$ (49)

We note that these parameters capture both innate and immune-mediated phagocytosis in chronic asymptomatic infections. We assume there is no phagocytosis of uRBCs or iRBCs in the peripheral circulation.

#### Final RBC equations.

We can now define the update rules for the RBC populations in the circulation and spleen, in terms of the equations defined above:

$$U_{c}(a,t)=\left\{ \begin{array}{cc} R_{c}(a,t) & \text{for }1<a\leq T_{R}\;\text{(reticulocytes)}\\ N_{c}(a,t) & \text{for }T_{R}<a\leq T_{U}\;\text{(normocytes)} \end{array}\right.$$ (50)

$$R_{c}(a,t)=\left\{ \begin{array}{cc} 0 & \text{for }a=1\\ \begin{array}{c} R_{c\to c}(a,t)+U_{s\to c}(a,t)+r_{\to c}(a,t)\\ -\nabla_{c\to c}(a,t)-\nabla_{s\to c}(a,t) \end{array} & \text{for }1<a\leq T_{M} \end{array}\right.$$ (51)

$$N_{c}(a,t)=\left\{ \begin{array}{cc} \begin{array}{c} R_{c\to c}(a,t)+U_{s\to c}(a,t)\\ -\nabla_{c\to c}(a,t)-\nabla_{s\to c}(a,t) \end{array} & \text{for }a=T_{M}+1\\ \begin{array}{c} N_{c\to c}(a,t)+U_{s\to c}(a,t)\\ -\nabla_{c\to c}(a,t)-\nabla_{s\to c}(a,t) \end{array} & \text{for }T_{M}+1<a\leq T_{U} \end{array}\right.$$ (52)

$$U_{s}(a,t)=U_{s\to s}(a,t)+U_{c\to s}(a,t)-\nabla_{s\to s}(a,t)$$ (53)

$$I_{c}(a,t)=\left\{ \begin{array}{cc} \nabla_{c}(t) & \text{for }a=1\\ I_{c\to c}(a,t)+I_{s\to c}(a,t) & \text{for }1<a\leq T_{irbc} \end{array}\right.$$ (54)

$$I_{s}(a,t)=\left\{ \begin{array}{cc} \nabla_{s}(t) & \text{for }a=1\\ I_{s\to s}(a,t)+I_{c\to s}(a,t) & \text{for }1<a\leq T_{irbc} \end{array}\right.$$ (55)

where the quantities of uRBCs and iRBCs that remain within the circulation and spleen are:

$$R_{c\to c}(a,t)=R_{c}(a-1,t-1)\cdot \exp[-\delta_{u}]$$ (56)

$$N_{c\to c}(a,t)=N_{c}(a-1,t-1)\cdot \exp[-\delta_{u}]$$ (57)

$$U_{s\to s}(a,t)=U_{s}(a-1,t-1)\cdot \exp[-\delta_{u}^{\prime }-\lambda_{u}]$$ (58)

$$I_{c\to c}(a,t)=I_{c}(a-1,t-1)\cdot \exp[-\delta_{i}-\zeta ]$$ (59)

$$I_{s\to s}(a,t)=I_{c}(a-1,t-1)\cdot \exp[-\delta_{i}^{\prime }-\lambda_{i}]$$ (60)

#### Differences between Pf and Pv.

In this model, Pf can be sequestered into the microvasculature (ζ>0) while Pv microvascular sequestration is negligible (ζ=0). The only other difference between the two species in this model is the age-dependent merozoite preference β(a) for uRBCs of age *a*, as illustrated in Fig J in S1 File.

Values for other biological parameters in the model are likely to differ between Pf and Pv, such as the parasite multiplication factor and the rate of iRBC removal from circulation due to differences in iRBC deformability. However, for simplicity we assumed identical values for these parameters for the two species. As the results of the sensitivity analysis show, the model dynamics are not particularly sensitive to the assigned values.

#### Fitting and sensitivity analysis.

The model comprises 36 parameters, see Table 4 for our chosen baseline values and parameter units. Further details are provided in the supplementary Model Description (S1 File).

**Table 4:** Baseline values and units for each model parameter.

Process	Parameter	Value	Units
Normoblast production	$e_{sl}$	16	—
	$U_{c}^{l}$	0.33	—
	$f_{max}$	10	—
Reticulocyte release	$\rho_{0}$	0.001	${\text{hour}}^{-1}$
	$\rho_{s}$	10	—
	$\rho_{i}$	0.5	—
	κ	10^−9^	${\text{RBC}}^{-1}$
uRBC removal	$\delta_{U}^{A}$	0.743	$\text{RBC}\cdot {\text{hour}}^{-1}$
	$\delta_{U}^{\min}$	$2.164\times 10^{-5}$	$\text{RBC}\cdot {\text{hour}}^{-1}$
	$\delta_{U}^{\max}$	1.207	$\text{RBC}\cdot {\text{hour}}^{-1}$
	$\delta_{U}^{c50}$	2954	hours
	$\delta_{U}^{g}$	43.73	—
	$k_{\nu }^{U}$	1	—
	$\delta_{50}^{U}$	10^−7^	—
uRBC release	mag	10	hours
	$\mu_{U}$	3.65	—
	$\sigma_{U}$	0.0025	—
RBC infection	*ω*	0.1	—
	PMF	8	—
	${sl}_{\beta }^{\text{Pf}}$	20	—
	$a_{\beta }^{50}$	80	days
	${sl}_{\beta }^{\text{Pv}}$	4.5	days
iRBC removal	$\delta_{iR}$	0.562	$\text{RBC}\cdot {\text{hour}}^{-1}$
	$\delta_{iS}$	1.124	$\text{RBC}\cdot {\text{hour}}^{-1}$
	$\delta_{I}^{sl}$	10	—
	$\delta_{I}^{c50}$	26	hours
	$k_{\nu }^{I}$	3	—
	$\delta_{50}^{I}$	10^−4^	—
iRBC release	*k*_*iR*_	0.03	—
	*k*_*iS*_	0.01	—
iRBC sequestration	$\zeta_{sl}$	10	—
	$\zeta_{50}$	26	hours
RBC destruction	$\lambda_{u}^{M}$	$5\times 10^{-7}$	${\text{hour}}^{-1}$
	$\lambda_{i}^{M}$	$1.5\times 10^{11}$	${\text{hour}}^{-1}$
	*k*_*M*_	0.01	—
	$\gamma_{M}$	0.25	—

We were unable to estimate the model parameters using standard model-fitting approaches, because longitudinal circulation and splenic RBC data were not available. Instead, previously-published studies were used to derive many parameter values, and key parameters were systematically varied within biologically plausible ranges to identify values that could explain the reported cross-sectional splenectomy data, under the assumption that these data characterised the steady-state dynamics of chronic asymptomatic malaria infections. Further, since we used subject-level data from two different cohorts (see Patient data), we calibrated the model against whole cohorts rather than individual subject data.

To quantify how well the model outputs agreed with the cross-sectional splenectomy data, we defined a relative error function that scaled the absolute error for a given model output $\hat{y}$ relative to the median and range of the values $y=\{y_{1},y_{2},\ldots \}$ reported in the splenectomy patients:


$$\mathrm{Err}(\hat{y},y)=\frac{\left|\log(\hat{y})-\log(\mathrm{med}(y))\right|}{\log(\max(y))-\log(\min(y))}$$

We calculated relative errors for the uRBC populations in the circulation and spleen (*U*_*c*_ and *U*_*s*_), for the iRBC populations in the circulation and spleen (*I*_*c*_ and *I*_*s*_), for the iRBC count ratio ($I_{s}:I_{c}$), and for the iRBC biomass ratio (the percentage of RBCs that are parasitised (a) in the spleen : (b) in the circulation). We defined the net error for a given simulation as the sum of these relative errors.

We undertook a sensitivity analysis in which we defined sampling distributions for each model parameter and for the initial RBC count *U*_*ss*_, used Latin hypercube sampling to draw 1000 samples for each parameter, and ran model simulations for Pf and Pv infections. We defined log-uniform distributions for parameters whose values might plausibly span multiple orders of magnitude, and defined uniform distributions for the remaining parameters. For each of the RBC populations, and for retention and removal ratios, we calculated median-centred 50% and 95% prior predictive intervals, which correspond to the 0.25–0.75 and 0.025–0.975 quantile intervals, respectively. We also calculated partial Spearman rank correlation coefficients between (a) each model parameter and the initial RBC count *U*_*ss*_; and (b) chronic infection steady-state outputs. The sampling distributions and supporting figures are included in the supplementary Sensitivity analysis (S2 File).

## Results

We begin by identifying plausible parameter combinations for which the model produces substantial splenic retention of iRBCs. We then demonstrate that, for some of these parameter combinations, the model is capable of producing chronic malaria infections that are consistent with the cross-sectional splenectomy dataset. For these parameter combinations, we show that uRBC retention in the spleen (attributable to malaria) is substantially higher than parasitisation of circulating uRBCs, for both Pf and Pv infections. Finally, we demonstrate that the model dynamics are not particularly sensitive to the choice of model parameter values, and that our primary findings are robust to the limits of our model calibration.

### Retention of infected RBCs in the spleen

A key characteristic in chronic *Plasmodium* infections is that the large majority of iRBCs are found in the spleen and a minority are present in the circulation [11]. For the model to retain the majority of iRBCs in the spleen, it must remove large quantities of iRBCs from the circulation and allow these retained iRBCs to remain in the spleen (i.e., avoid phagocytosis). Accordingly, we focused on the following RBC movement rates:

1. The removal rate of iRBCs from the circulation $\delta_{i}$, governed by the model parameter $\delta_{iR}$ (removal rate of ring iRBCs); and
2. The phagocytosis rate of iRBCs in the spleen $\lambda_{i}$, governed by the model parameter $\lambda_{i}^{M}$ (iRBC phagocytosis rate per individual macrophage).

We systematically varied these two parameters, and observed that removal rates $\delta_{iR}\geq 0.1$ result in chronic infections where the majority of iRBCs are retained in the spleen (Fig 2). The absolute removal rates of uRBCs and iRBCs into the spleen increase with the circulating parasite load, from baseline values for $\leq 10^{7}$ circulating iRBCs, up to a four-fold increase for $\geq 10^{11}$ circulating iRBCs. The removal rate $\delta_{iR}=0.1$ corresponds to hourly removal probabilities of 0.095 (at baseline) to 0.33 (high parasite load).

### Comparison to splenectomy patient data

A comparison of the model results to the absolute counts of peripheral and splenic uRBC and iRBC counts observed in the splenectomy patients is shown in Fig 3. While the circulating and splenic uRBC counts were relatively stable for removal rates $\delta_{iR}\geq 0.05$ across a wide range of phagocytosis rates, the iRBC counts exhibited much greater sensitivity to both parameters.

**Fig 4:**
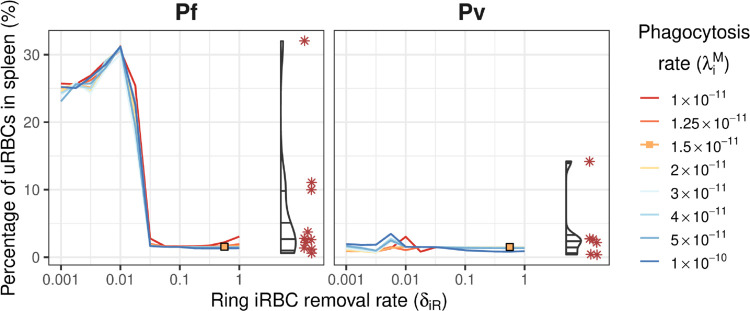
The steady-state percentage of total-body uRBCs that are retained in the spleen (*U*_*s*_). Percentages are shown for a range of values for ring iRBC removal from circulation ($\delta_{iR}$) and iRBC phagocytosis in the spleen ($\lambda_{i}^{M}$), shown as coloured lines. Corresponding data in the splenectomised patients (Pf: n=9; Pv: n=6) are shown on the right of each plot for comparison (violin plots for distribution and red asterisks for observed data points). Orange squares indicate good agreement between the model and the data.

**Fig 5:**
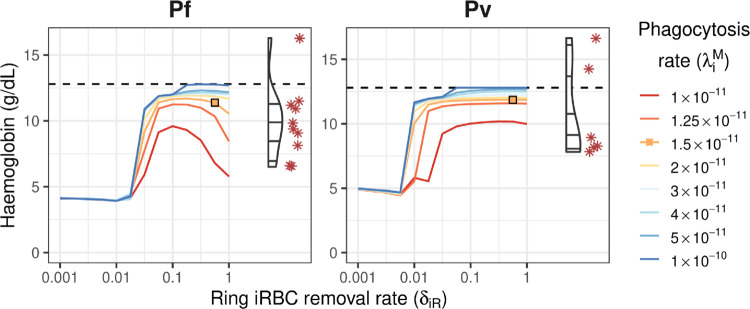
Steady-state haemoglobin levels. Haemoglobin levels are shown for a range of values for iRBC removal from circulation ($\delta_{iR}$) and iRBC phagocytosis in the spleen ($\lambda_{i}^{M}$), shown as coloured lines. Corresponding data in the splenectomised patients (Pf: n=9; Pv: n=6) are shown on the right of each plot for comparison (violin plots for distribution and red asterisks for observed data points). Orange squares indicate good agreement between the model and the data. Dashed lines indicate the baseline haemoglobin level of 12.8 g/dL for endemic healthy controls [16].

Based on the iRBC count ratio results (Fig 2), we considered parameter combinations where $\delta_{iR}\in [0.1,1]$. For Pf infections, the net error was smallest for $\lambda_{i}^{M}\in [1.25\times 10^{-11},1.5\times 10^{-11}]$ and $\delta_{iR}\in [0.1,0.6]$. For Pv infections, the net error was smallest for $\lambda_{i}^{M}\in [1\times 10^{-11},2\times 10^{-11}]$ and $\delta_{iR}\in [0.3,0.6]$. In Figs 3–5 we highlight the parameter combination for which the sum of the Pf net error and Pv net error was smallest ($\lambda_{i}^{M}=1.5\times 10^{-11}$, $\delta_{iR}=0.562$); this combination resulted in the third-smallest net error for each of Pf and Pv. With this parameter combination, approximately 1.45% of uRBCs were retained in the spleen for both Pf and Pv infections. For comparison, in the absence of infection approximately 0.13% of uRBCs were retained in the spleen.

In 11 individuals with asymptomatic infections, less than 5% of total-body RBCs were retained in the spleen (Pf: 6 of 9; Pv: 5 of 6) [11]. The model yielded similar steady-state proportions for ring iRBC removal rates $\delta_{iR}\geq 0.1$, as shown in Fig 4. The remaining 4 individuals had massively enlarged spleens containing 10–32% of total-body RBCs, with the model yielding similar steady-state proportions for Pf infections at substantially lower ring iRBC removal rates $\delta_{iR}\leq 0.03$, also shown in Fig 4.

These lower iRBC removal rates result in extreme anaemia, with haemoglobin levels as low as 5 g/dL (shown in Fig 5), and extremely high parasite levels (Fig 3). Lower iRBC removal rates also caused the model dynamics to become particularly sensitive to the rates of uRBC and iRBC retention, and phagocytosis, as evident in Fig 4 for $\delta_{iR}\leq 0.03$.

### Model dynamics: uRBC loss and iRBC biomass

There are two processes in the model that remove uRBCs from the circulation: parasitisation by merozoites released by mature iRBCs, and retention of uRBCs in the spleen. In the model, the presence of iRBCs in the circulation results in a fold increase in uRBC removal from the circulation, and we can calculate the increase in uRBC retention in the spleen that is caused by infection.

**Fig 6:**
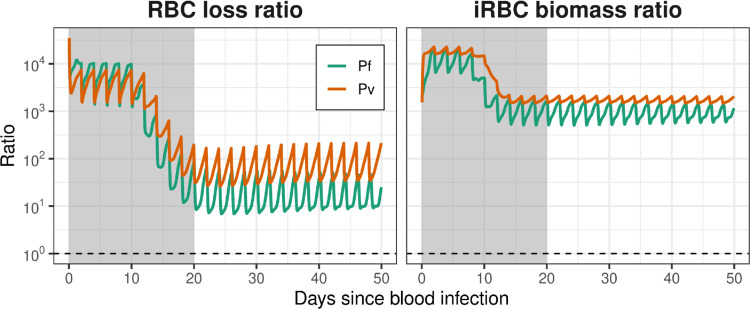
Time-varying ratios of uRBC loss and iRBC biomass. Left panel: the ratio of circulating uRBC loss due to increased retention in the spleen, versus the loss from circulation due to infection by malaria parasites. Right panel: ratio of iRBC biomass in the spleen to the iRBC biomass in the circulation. Dashed lines indicate ratios of 1:1. Shaded intervals indicate the acute infection phase (i.e., the transition from initial infection to chronic infection), which the model dynamics do not capture. Results are shown for the baseline parameter values listed in Table 4.

As shown in Fig 6 (left panel), this malaria-associated retention of uRBCs in the spleen is substantially greater than the direct loss of circulating uRBCs due to parasitisation. In chronic infections (day 20 onwards), parasitisation causes 5.5% of uRBC loss for Pf and 1.2% of uRBC loss for Pv, and the ratio of circulating uRBCs lost to splenic retention per circulating uRBC lost to parasitisation is 17:1 for Pf and 82:1 for Pv.

The ratio of iRBC biomass (the percentage of RBCs that are parasitised) in the spleen versus in the circulation is shown in Fig 6 (right panel). This ratio has been used by, e.g., Kho et al. [11] to characterise splenic tropism. The chronic steady-state ratios are 918:1 for Pf and 1701:1 for Pv; these values are similar orders of magnitude to those reported by Kho et al. [11] (Pf: mean 289, range 18–1530; Pv: mean 3590, range 2300–4210).

The difference in uRBCs loss and iRBC biomass ratios between Pf and Pv infections is primarily due to our assumptions regarding sequestration. If we make the counterfactual assumption that Pv-infected RBCs can be sequestered into the microvasculature at the same age-dependant rate ζ as for Pf infections, we obtain ratios very similar to those for Pf (17:1 and 943:1, respectively). Despite the *in vitro* ability of mature Pv-iRBC to adhere to endothelial cells, this is 10-fold lower than that seen with Pf-iRBC [48], and the available histopathological vivax literature supports our assumption of minimal extra-splenic microvascular sequestration in vivax malaria [29,49,50].

Recall that uRBC and iRBC phagocytosis rates in the model are directly proportional to the splenic macrophage population, and that increased RBC retention in the spleen stimulates an increase in the macrophage population. Relative to homeostasis, the phagocytosis rates are 11 times higher in chronic Pf and Pv infections (day 20 onwards).

### Sensitivity analysis

Across the 1,000 parameter samples for each species, the smallest net errors for Pf and Pv were both slightly larger than the smallest net errors for Pf and Pv obtained in our 2-parameter sweep for our chosen baseline parameter values (see Table 5). From the 2-parameter sweep, the parameter combination with the smallest combined Pf and Pv error ($\lambda_{i}^{M}=1.5\times 10^{-11}$, $\delta_{iR}=0.562$) also had a smaller net error for Pf than all sensitivity analysis simulations, but had a larger net error for Pv (Table 5).

**Table 5:** The smallest net errors for Pf and Pv simulations, reported across the 1,000 sensitivity analysis simulations for each species (top row), across the parameter sweep for $\lambda_{i}^{M}$ and $\delta_{iR}$ (middle row), and for our chosen parameter combination $\lambda_{i}^{M}=1.5\;\times \;10^{-11}$, $\delta_{iR}=0.562$ (bottom row).

Simulation	Net Error: Pf	Net Error: Pv
Best in sensitivity analysis	0.295	1.16
Best in 2-parameter sweep	0.224	1.11
Best Pf+Pv in 2-parameter sweep	0.244	1.42

The prior predictive intervals (shown in S2 File) are sufficiently broad that they span the range of the splenectomy patient data, except for the highest recorded Pv splenic uRBC population. In particular, the model yields iRBC biomass ratios that span the full range of the splenectomy patient data. The most striking difference between the prior predictive intervals for Pf and Pv are:

- The splenic uRBC population is evenly distributed above and below the baseline results for Pf, but mostly lies below the baseline results for Pv;
- Accordingly, the uRBC retention ratio is evenly distributed above and below the baseline results of Pf, but mostly lies below the baseline results for Pv;
- In contrast, the iRBC biomass ratio mostly lies below the baseline results for Pf, and is more evenly distributed above and below the baseline results for Pv.

Since we have used identical parameter values for the Pf and Pv simulations, the only differences in the model between these two species are (a) the age-dependent merozoite preferences for uninfected uRBCs; and (b) the ability for Pf-infected RBCs to become sequestered in the microvasculature, from where they can release merozoites into the circulation, but are protected from removal into the spleen.

An inspection of the partial rank correlation coefficients between model parameters and chronic infection steady-state outputs (shown in S2 File) shows that the initial RBC count *U*_*ss*_ strongly influences the circulating uRBC count and has smaller influence on the splenic uRBC count and the circulating and splenic iRBC counts. The threshold parameter for increased RBC production ($U_{c}^{l}$) influences the circulating and splenic uRBC counts, while the release rate of reticulocytes before they reach maturation age ($\rho_{0}$) strongly influences all model outputs except the splenic macrophage population.

Out of the uRBC removal and release parameters, the maximal fold-increase in uRBC removal ($k_{\nu }^{U}$) has the largest correlation with many outputs. In comparison, the maximal fold-increase in iRBC removal ($k_{\nu }^{I}$) has only a modest influence on the circulating and splenic iRBC counts. Out of the iRBC infection, removal, and release parameters, the largest correlations with the iRBC biomass and RBC loss ratios are shown by the proportion of merozoites released in the spleen that infect RBCs in the spleen (*ω*), the ring iRBC removal rate ($\delta_{iR}$), and the ring iRBC release rate scaling factor (*k*_*iR*_). For Pf infections, the iRBC sequestration parameters (shaded in orange) also have some effect on the iRBC biomass and RBC loss ratios. Finally, the iRBC phagocytosis parameter ($\lambda_{i}^{M}$) has large correlations with iRBC counts in the circulation and spleen, with the splenic macrophage population, and the uRBC retention ratio.

These observations suggest that the parameters that most strongly influence how well the model can characterise the cross-sectional patient data span (a) RBC production and release; (b) the maximal fold-increase in uRBC removal; (c) the rates at which iRBCs are removed and released; and (d) the iRBC phagocytosis rate.

We also noted differences in coefficient signs for Pf and Pv, driven by the differences in age-preference β(a) and/or Pf sequestration into the microvasculature (ζ>0). For Pf, the maximal fold-increase in uRBC removal ($k_{\nu }^{U}$) was negatively correlated with the splenic uRBC count, the ring iRBC removal rate ($\delta_{iR}$) was negatively correlated with the circulating iRBC count and positively correlated with splenic iRBC count, and the iRBC phagocytosis parameter ($\lambda_{i}^{M}$) was positively correlated with the circulating iRBC count. For Pv, each of these correlations was in the reverse direction. We ran simulations that explore a range of sequestration rates for Pv and observed that the iRBC biomass and RBC loss ratios approached those obtained for Pf as the Pv sequestration rate approached that of Pf (see S1 File), which suggests that it is this difference between the two species in the model that drives these differences in outputs.

Note that while the maximum fold-increase parameters $k_{\nu }^{U}$ and $k_{\nu }^{I}$ affect the splenic retention of uRBCs and iRBCs, respectively, the values of these parameters **are not equal** to the actual fold-increases in uRBC and iRBC retention. For example, using our baseline values we have a maximum 2-fold increase in uRBC removal rate, but both Pf and Pv infections result in 10-fold increases in uRBC retention.

## Discussion

To our knowledge, this is the first mathematical model of Pf and Pv infections that describes the dynamics of uninfected and infected RBCs with inclusion of the spleen as a separate compartment where RBCs can be retained and phagocytosed. In this model, the spleen significantly influences whole-body RBC dynamics and the relationship between parasitaemia (parasite counts in the circulation) and non-circulating parasite load. Through simulations using biologically plausible parameter values, we were able to produce chronic malaria infections where the spleen retained an overwhelming majority of iRBCs, and also retained large quantities of uRBCs. This vision is consistent with early speculations triggered by the observation of innate retention of ring-iRBC in human spleens *ex vivo* [14], pointing to stringent retention in chronic Pf infections [51]. Our results are also consistent with experimental observations in the spleen and peripheral blood of asymptomatic adults naturally infected with Pf and Pv in Papua, Indonesia [11,12].

Our model simulations showed that large quantities of parasites can be retained in the spleen when the iRBC retention rate is sufficiently high, and the splenic phagocytosis rate is low enough to allow for persistent parasitaemia. In these chronic infections, splenic retention of uRBCs and iRBCs stimulates a ten-fold increase in the splenic macrophage population, from an initial value of $1.2\times 10^{9}$ up to $13.1\times 10^{9}$ for Pf and $12.9\times 10^{9}$ for Pv. With our chosen iRBC phagocytosis rate ($\lambda_{i}^{M}=1.5\times 10^{-11}$ per macrophage), this corresponds to hourly phagocytosis probabilities of 0.178 for Pf and 0.176 for Pv. These phagocytosis rates are insufficient to control and overcome accumulation of parasites in the spleen.

The removal of uRBCs from circulation due to splenic retention was shown to be tens of times larger than the uRBC loss due to parasitisation in chronic infections, with the ratio of circulating uRBCs lost to splenic retention per circulating uRBC lost to parasitisation estimated to be 17:1 for Pf (5.5% due to parasitisation) and 82:1 for Pv (1.2% due to parasitisation). These ratios are larger than previous estimates for uRBC loss in acute clinical infections. These studies estimated that circulating RBC loss due to parasitisation, as a proportion of overall circulating RBC loss, was 10.5% (a ratio of 1:8.5) for acute Pf malaria in non-immune neurosyphilis patients [18]; 7.9% (a ratio of 1:11.7) for acute clinical Pf malaria [19]; 2.9% (a ratio of 1:33.5) for acute Pv malaria in non-immune neurosyphilis patients [20]; and 0.015% in Pf and Pv induced blood stage malaria volunteer infection studies [21]. In our model simulations this proportion differs by several orders of magnitude between the initial and chronic stages of asymptomatic Pf infections (0.01% and 5.5%, respectively) and asymptomatic Pv infections (0.02% and 1.2%, respectively). While our model was not calibrated to characterise acute symptomatic infections, the very early stage proportions in our model are consistent with the 0.015% reported in pre-symptomatic stages of Pf and Pv infection as reported by Woolley et al. [21]. This finding is consistent with major variations in the proportion of uRBC removed by the spleen versus parasitisation across the course of infection.

In order for our model to reproduce the highest levels of uRBC retention observed in the asymptomatic Papuan data (≥10%), we had to substantially decrease the iRBC removal rate. This resulted in greater parasitaemia and increased uRBC splenic retention, resulting in extremely low haemoglobin levels (5–7 g/dL). In a follow-up study of splenic RBC retention in 37 adult Papuans who underwent splenectomy for trauma or hyperreactive splenomegaly, Kho et al. [12] reported that splenic RBC load was negatively correlated with haemoglobin levels, reflecting lower numbers of circulating uRBCs in patients with splenomegaly. Patients with the highest levels of RBC retention by spleen-mimetic filtration in vivo (≥10%) had haemoglobin levels of ≈6 g/dL, consistent with the results obtained from our model.

Primary limitations of this model include the simplicity of the splenic retention rates, which does not explicitly account for the impact of RBC congestion in the spleen that may affect the ability for RBCs to flow through the spleen and return to the circulation, as suggested by the rapid retention of heated RBCs in the presence of splenomegaly [31]. We further assumed that the splenic macrophage population is proportional to the total quantity of retained RBCs, rather than distinguishing between retained uRBCs and retained iRBCs, due to an absence of data that might inform a quantifiable effect. Our model also did not account for erythropoiesis events occurring in the spleen during malaria [52], however its contribution to the size of splenic uRBC populations is likely negligible. Finally, the model does not explain the mechanics by which the splenic phagocytosis capacity might be dysfunctional or simply overwhelmed in immune or semi-immune individuals such as the Papuan patients. A recent observation in sickle cell disease has shown that the splenic density of macrophages was normal but not adapted to the intensity of congestion [53].

The primary limitation of our analysis is the lack of longitudinal RBC data for asymptomatic individuals, which meant that we were only able to calibrate the model against the reported cross-sectional data. For this reason we kept parameter values constant, rather than allowing them to vary over time. Accordingly, the model results for the initial stages of infection do not necessarily reflect the true progression from initial exposure to chronic infection, since many of these parameters will vary over the course of an infection. We also used identical values for Pf and Pv infections where possible, and the sensitivity analysis demonstrated that the model results were not sensitive to moderate adjustments to our reference values. However, parameters such as the parasite multiplication factor likely differ for Pf and Pv, given differing abundance of target cells. We note that cross-sectional studies in other populations (e.g., across Asia and Africa) and longitudinal studies of malaria in which a small number of participants may undergo splenectomy could provide data and insights that would make more robust analysis feasible in the future.

In summary, we have established the first modelling framework to study RBC dynamics in Pf and Pv infections that includes the spleen as a separate compartment where iRBCs and uRBCs can be retained and phagocytosed, consistent with recent knowledge advancements in malaria biology involving the spleen as a hidden compartment. Our findings are consistent with experimental data and suggest that the spleen can act as a reservoir for iRBCs and sustain chronic asymptomatic malaria infections, when the iRBC retention rate exceeds the phagocytosis capacity of the splenic macrophage population in the red-pulp. By quantifying RBC loss in this model, we also show that the vast majority of RBCs lost in chronic malaria are lost to splenic retention of uRBCs and not to obligatory destruction of iRBCs.

## Supporting information

S1 File Model description. Defines the model equations and parameters.
(PDF)

S2 File Sensitivity analysis. Presents the results of our sensitivity analysis.
(PDF)
