# Supplementary material for: The role of the spleen in red blood cell loss caused by malaria: A mathematical model
Source: PLoS Comput Biol. 2026 Jan 12;22(1):e1013865. doi: 10.1371/journal.pcbi.1013865 (PMC12810929; doi:10.1371/journal.pcbi.1013865)
Supplement: S1 File — Defines the model equations and parameters. (PDF) [file pcbi.1013865.s001.pdf]

Model Description

Contents

1 Compartments and cell populations 2

2 Homeostasis and initial state 3

3 RBC production in the bone marrow 7

4 RBC release from the bone marrow 9

5 Uninfected RBC removal from circulation 11

6 Uninfected RBC return to circulation 13

7 RBC infection 14

8 Infected RBC removal from circulation 16

9 Infected RBC return to circulation 19

10 Infected RBC sequestration 21

11 RBC destruction in the spleen 23

12 Differences between Pf and Pv 24

13 Final RBC equations 25

14 Baseline outputs: no infection 26

15 Baseline outputs: Pf 28

16 Baseline outputs: Pv 32

17 Exploring Pv sequestration 36

18 Model parameters 37

References 39

1 Compartments and cell populations

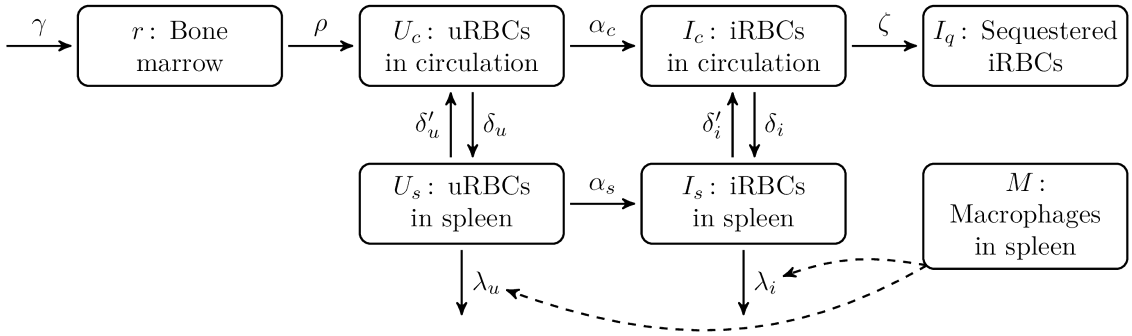

Figure A: An overview of the model structure.

Table A: The cell populations in the model, with respect to age a and time t where appropriate.

Symbol	Compartment	Cell type	Lifespan (days)
$r(a,t)$	Bone marrow	Normoblasts and reticulocytes	$T_R = 3.5$
$R_c(a,t)$	Circulation	Uninfected reticulocytes	$T_M = 4.5$
$N_c(a,t)$	Circulation	Uninfected normocytes	$T_N = 115.5$
$U_c(a,t)$	Circulation	Uninfected RBCs	$T_U = 120$
$I_c(a,t)$	Circulation	Infected RBCs	$T_I = 2$
$I_q(a,t)$	Microvasculature	Infected RBCs	$T_I = 2$
$U_s(a,t)$	Spleen	Uninfected RBCs	$T_U = 120$
$I_s(a,t)$	Spleen	Infected RBCs	$T_I = 2$
$M(t)$	Spleen	Macrophages	—

For convenience, we also define terms for the total RBC populations in the circulation and the spleen:

$$\mathbf{U_c}(t) \equiv \sum_a U_c(a,t) \tag{1}$$

$$\mathbf{U_s}(t) \equiv \sum_a U_s(a,t) \tag{2}$$

$$\mathbf{I_c}(t) \equiv \sum_a I_c(a,t) \tag{3}$$

$$\mathbf{I_s}(t) \equiv \sum_a I_s(a,t) \tag{4}$$

2 Homeostasis and initial state

Some model parameters are defined relative to the steady-state (homeostasis) in the absence of a malaria infection, where the circulating reticulocyte and normocyte populations — $R_c(a,t)$ and $N_c(a,t)$, respectively — are equal to the steady-state RBC population U_{ss}. The value of U_{ss} is the median RBC count (in the circulation) for patients with no fever (`fever24 == 0`) or malaria infection (`Species == 0`).

```
utils::data("rbc_steady_state", package = "spleenrbc")
rbc_steady_state
#> [1] 1.75197e+13
```

We use a root-finding method to solve the following conservation equation for the normoblast production rate γ *in the absence of a malaria infection*:

$$U_{ss} \approx \sum_a N_c(a) + \sum_a R_c(a) : r(1) = \gamma \tag{5}$$

```
p <- baseline_parameters("Pf")
steady_state <- retic_steady_state(p)
print(steady_state$gamma)
#> [1] 7202963644
print(sum(steady_state$r_a))
#> [1] 580623948414
print(sum(steady_state$R_a))
#> [1] 181144300035
print(sum(steady_state$N_a))
#> [1] 1.733856e+13
print(sum(steady_state$Ur_a))
#> [1] 23347264546
```

Note that **only a very small fraction** of the RBCs are retained in the spleen at homeostasis:

```
rbc_spleen <- sum(steady_state$Ur_a)
rbc_circ <- sum(c(p$R_a_ss, p$N_a_ss))
pcnt_in_spleen <- 100 * rbc_spleen / (rbc_spleen + rbc_circ)
cat("Retained RBCs:", sprintf("%0.2f%%", pcnt_in_spleen), "\n")
#> Retained RBCs: 0.13%
```

Table B: The steady-state model parameters. Note that some are age-dependent.

Symbol	Description	Baseline value
U_{ss}	RBC population	1.75197×10^{13}
$r_{ss}(a)$	Reticulocyte population (bone marrow)	5.8062395×10^{11}
$R_{ss}(a)$	Reticulocyte population (circulation)	1.811443×10^{11}
$N_{ss}(a)$	Normocyte population (circulation)	1.7338556×10^{13}

Symbol	Description	Baseline value
$U_{r,ss}(a)$	RBC population (spleen)	2.3347265×10^{10}
M_0	Initial macrophage population	1.2×10^9
I_0	Initial infected RBC population	100

```
s0 <- initial_spleenrbc_state(p)
```

These steady-state parameters define the initial (uninfected) cell populations:

$$r(a, t=0) = r_{ss}(a) \tag{6}$$
$$R_c(a, t=0) = R_{ss}(a) \tag{7}$$
$$N_c(a, t=0) = N_{ss}(a) \tag{8}$$
$$U_s(a, t=0) = U_{r,ss}(a) \tag{9}$$
$$M(t=0) = M_0 \tag{10}$$

We start with a small number I_0 of infected RBCs in the circulation, with a small bias towards middle-aged cells (using a truncated normal distribution), and some infected RBCs in the spleen and the microvasculature:

$$I_c(a, t=0) = I_0 \cdot f_X(a) : a \sim \mathcal{N}(\mu = 20, \sigma = 30) \tag{11}$$
$$I_q(a, t=0) = I_c(a, t=0) \cdot (1 - \exp[-\zeta]) \tag{12}$$
$$I_s(a, t=0) = I_c(a, t=0) \cdot (1 - \exp[-\delta_i]) \cdot \exp(-\lambda_i) \tag{13}$$

The initial cell populations for the baseline parameter values are shown in Figures B, C, and D.

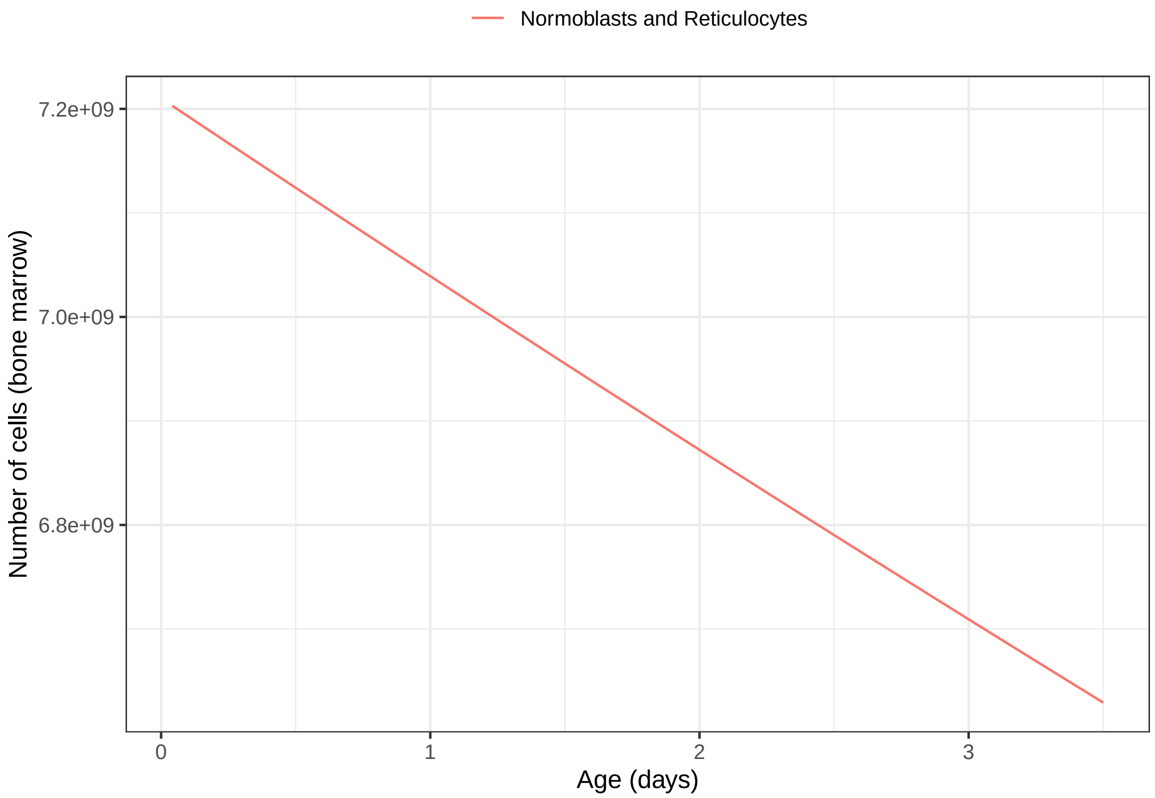

Figure B: Initial cell populations in the bone marrow.

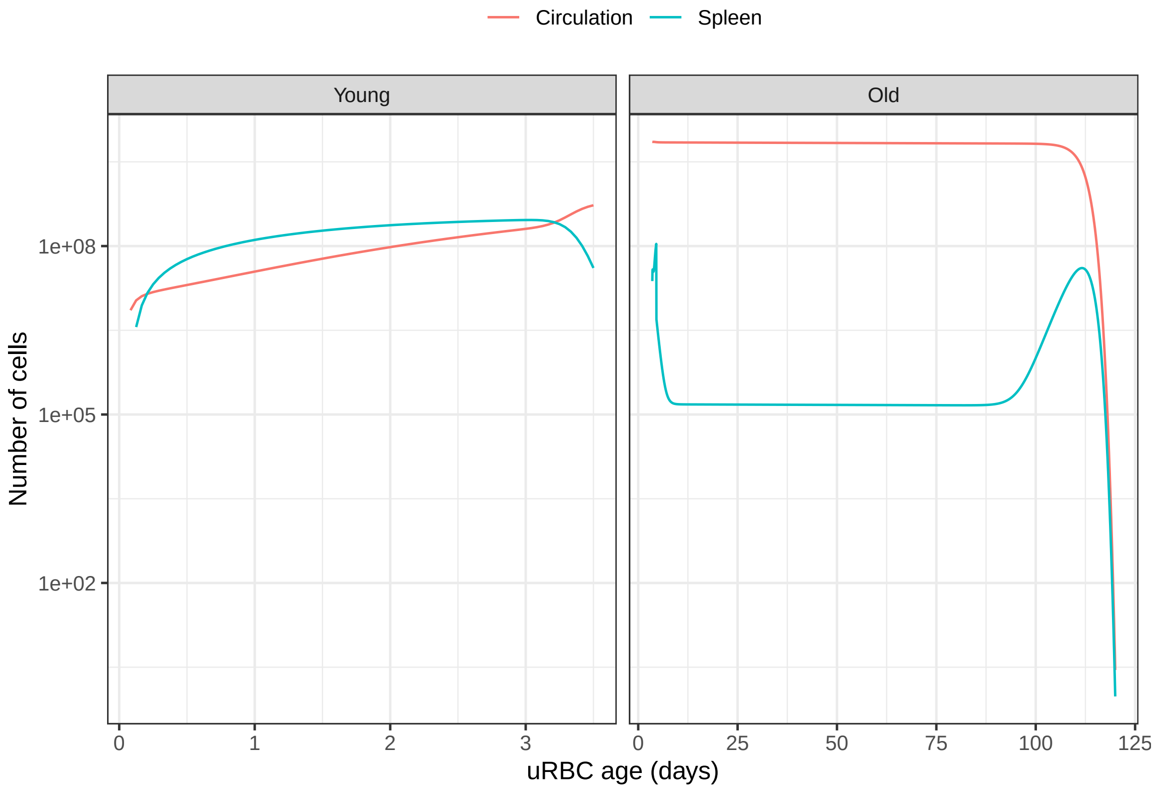

Figure C: Initial uninfected RBC populations.

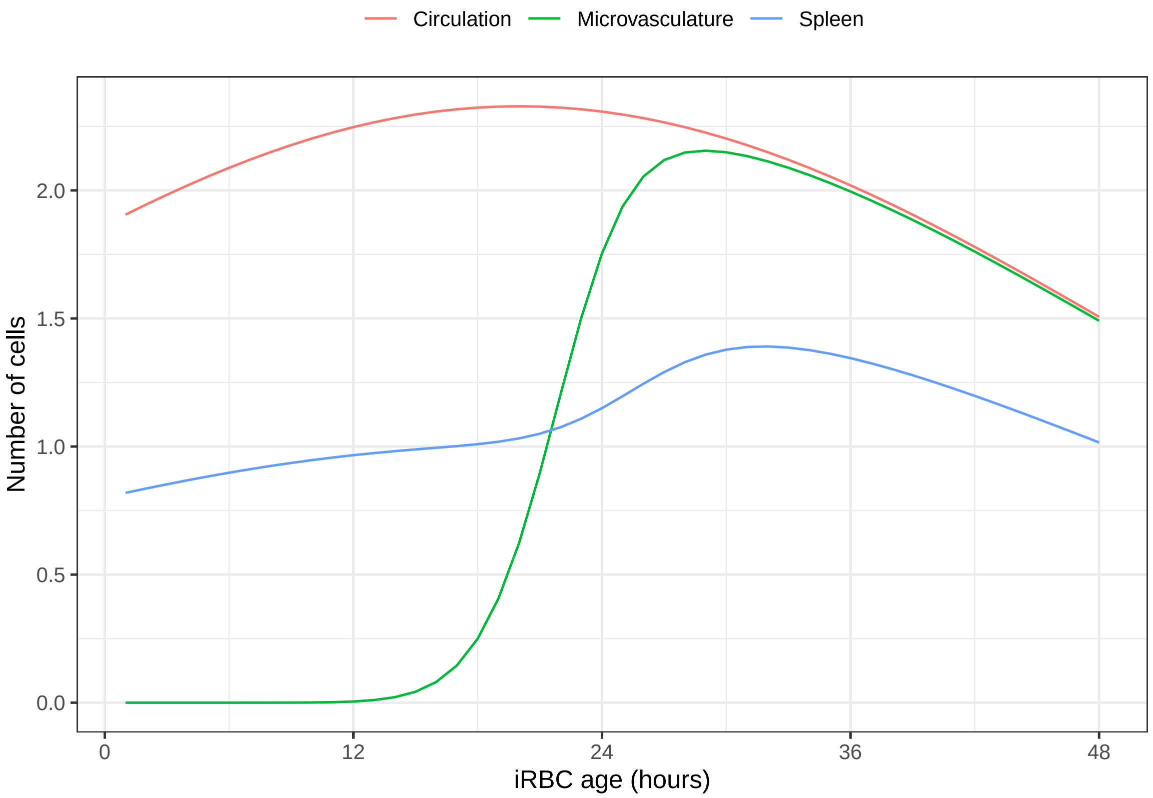

Figure D: Initial infected RBC populations.

3 RBC production in the bone marrow

The normoblast production rate depends on the population of circulating uninfected RBCs $\mathbf{U_c}(t)$, the steady-state uninfected RBC population U_{ss}, the steady-state normoblast production rate γ, and the threshold parameter U_c^l:

$$U_l = U_c^l \cdot U_{ss} \tag{14}$$

$$\text{eryth}(t) = \gamma \cdot \left[1 + \frac{f_{\max} - 1}{2} \cdot \left[1 - \tanh(e_{\text{sl}} \cdot \frac{\mathbf{U_c}(t) - U_l}{U_l})\right]\right] \tag{15}$$

Table C: Model parameters for erythropoiesis.

Symbol	Description	Baseline value
γ	Steady-state normoblast production	7.2029636×10^9
U_c^l	Threshold parameter	0.33
$f_{\max}$	Scaling parameter	10
e_{sl}	Slope parameter	16

Our chosen value of $f_{\max}$ is consistent with a previous modelling study (Watson et al., 2017), which noted that "in extreme anaemia [RBC production] can be increased fivefold or more", and produced model fits where RBC production was increased almost ten-fold.

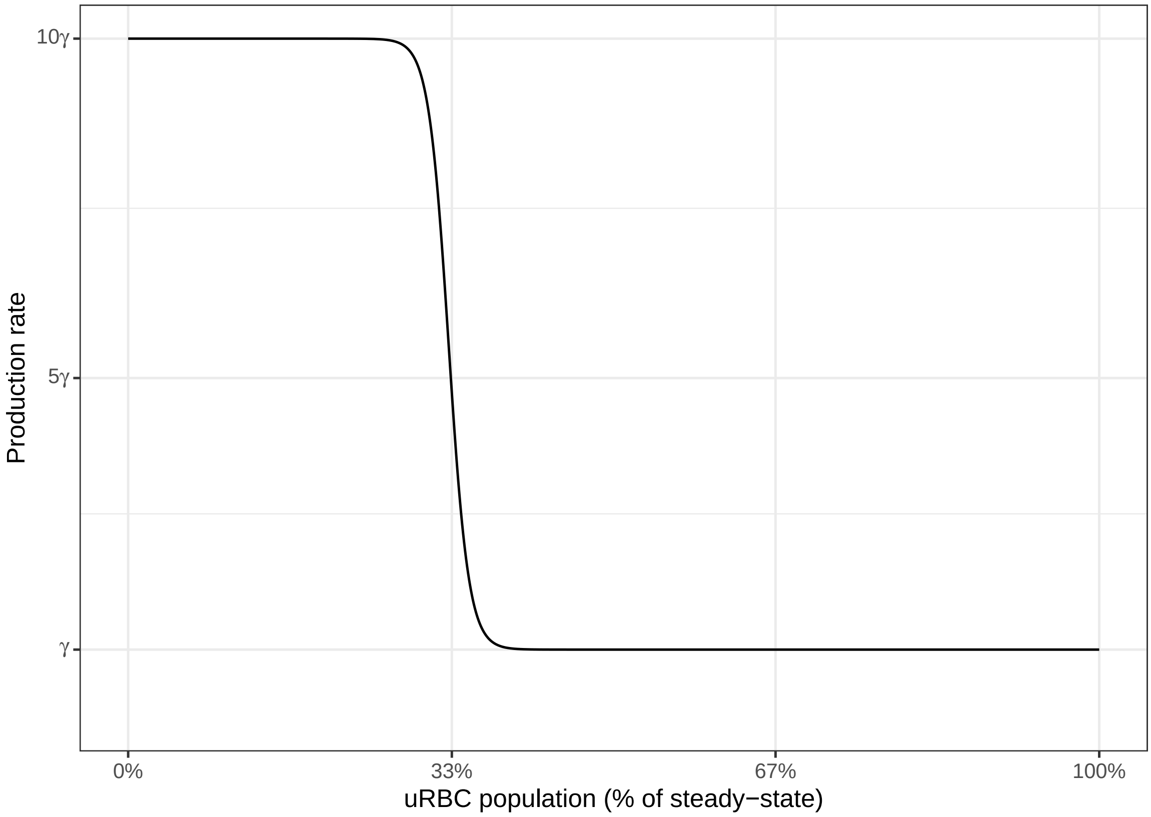

Figure E: The erythropoiesis rate varies from γ to $f_{\max} \cdot \gamma$.

4 RBC release from the bone marrow

The normoblast and reticulocyte populations in the bone marrow age over time, and the reticulocytes move into the circulation at the age-specific rate $\rho(a,t)$:

$$r(a,t) = \begin{cases} \text{eryth}(t) & \text{for } a = 1 \\ r(a-1,t-1)\cdot\exp[-\rho] & \text{for } 1 < a \le T_R \end{cases} \tag{16}$$

$$\rho(a,t) = \begin{cases} 10\cdot\min(20,\exp[\kappa\cdot(U_{ss}-\mathbf{U_c}(t))]) & \text{for } a \ge t_r \\ \rho_o\cdot\min(20,\exp[\kappa\cdot(U_{ss}-\mathbf{U_c}(t))]) & \text{for } a < t_r \end{cases} \tag{17}$$

$$t_r = \begin{cases} T_R & \text{when } \mathbf{U_c}(t) > U_{ss} \\ T_R^{\min} + (T_R - T_R^{\min})\cdot\left(1+\exp\left[-\rho_s\cdot\left(\frac{\mathbf{U_c}(t)}{U_{ss}}-\rho_i\right)\right]\right)^{-1} & \text{when } \mathbf{U_c}(t) \le U_{ss} \end{cases} \tag{18}$$

Table D: Model parameters for bone marrow cell equations.

Symbol	Description	Baseline value
ρ_0	Minimum release rate	0.001
ρ_s	Slope parameter	10
ρ_i	Inflection parameter	0.5
κ	Scaling factor	10^{-9}
$T_R^{\min}$	Minimum retention time	24 hours
T_R	Reticulocyte release time	84 hours

These parameters were calibrated using data from Koepke and Koepke (1986), as shown in Figure F.

We denote the number of reticulocytes released from the bone marrow in a time-step as $r_{\to c}$:

$$r_{\to c}(a,t) = r(a-1,t-1)\cdot(1-\exp[-\rho]) \tag{19}$$

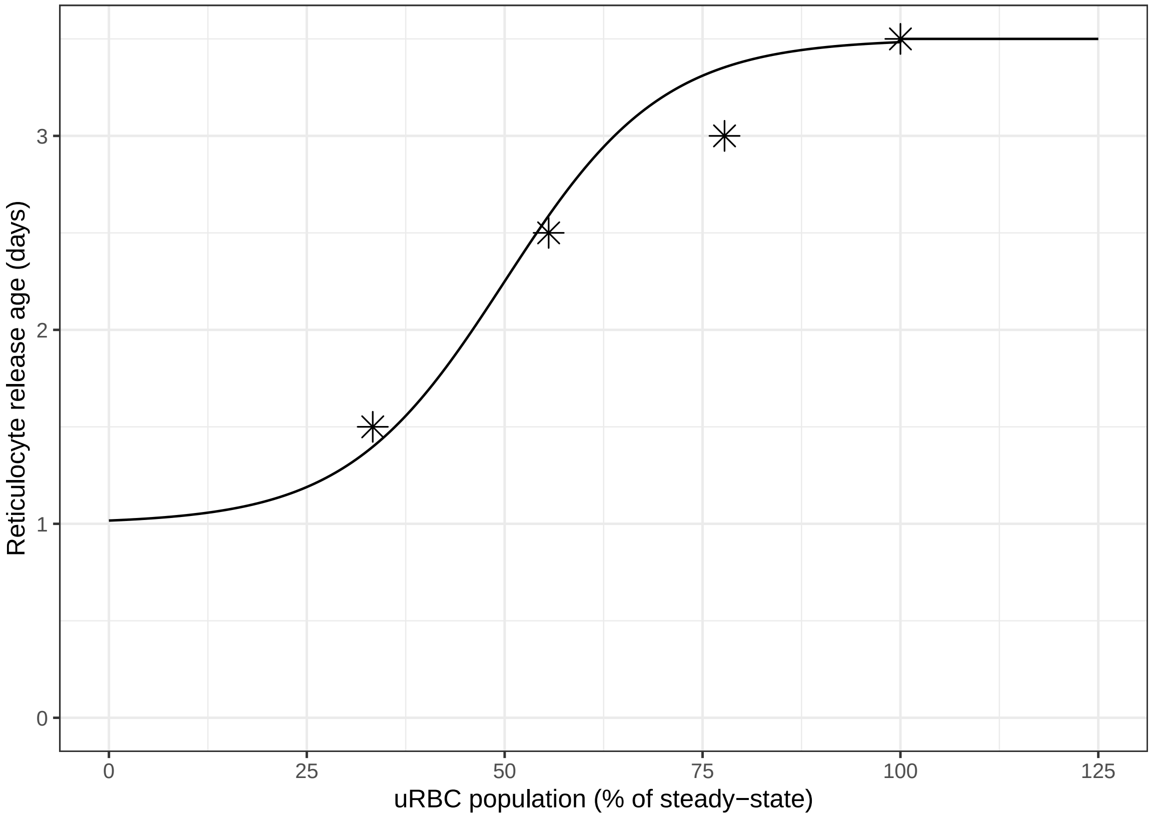

Figure F: The minimum age at which reticulocytes are released from the bone marrow. Point estimates are from Figure 2 of Koepke and Koepke (1986).

5 Uninfected RBC removal from circulation

The removal rate δ_u of uninfected RBCs from the circulation into the spleen depends on a number of offset, slope, and scaling parameters:

$$\delta_u(a,t) = F_U(t) \cdot \left(\delta_U^A \exp[-k_1 \cdot (a-1)] + \nu + \delta_U^{\min} + (\delta_U^{\max} - \delta_U^{\min}) \cdot \frac{(a-1)^{\delta_U^g}}{(a-1)^{\delta_U^g} + (\delta_U^{c50})^{\delta_U^g}} \right) \tag{20}$$

$$F_U(t) = 1 + k_\nu^U \cdot \frac{\mathbf{I_c}(t-1)^{g_d^U}}{\mathbf{I_c}(t-1)^{g_d^U} + \left(\delta_{50}^U \cdot [\mathbf{I_c}(t-1) + \mathbf{U_c}(t-1)]\right)^{g_d^U}} \tag{21}$$

$$k_1 = -\frac{\log\left(\frac{\delta_U^{\min}}{\delta_U^A}\right)}{23 \cdot 7} \tag{22}$$

Symbol	Description	Baseline value
δ_U^A	Scaling parameter	0.74303
$\delta_U^{\min}$	Scaling parameter	2.16405×10^{-5}
$\delta_U^{\max}$	Scaling parameter	1.206914
δ_U^{c50}	Half-maximal age	2954.306
δ_U^g	Scaling parameter	43.73335
ν	Offset parameter	0
k_ν^U	Scaling parameter	1
g_d^U	Slope parameter	1
δ_{50}^U	Scaling parameter	10^{-7}

We denote the number of uRBCs removed in a time-step as $U_{c\to s}$:

$$U_{c\to s}(a,t) = U_c(a-1, t-1) \cdot (1 - \exp[-\delta_u(a,t)]) \tag{23}$$

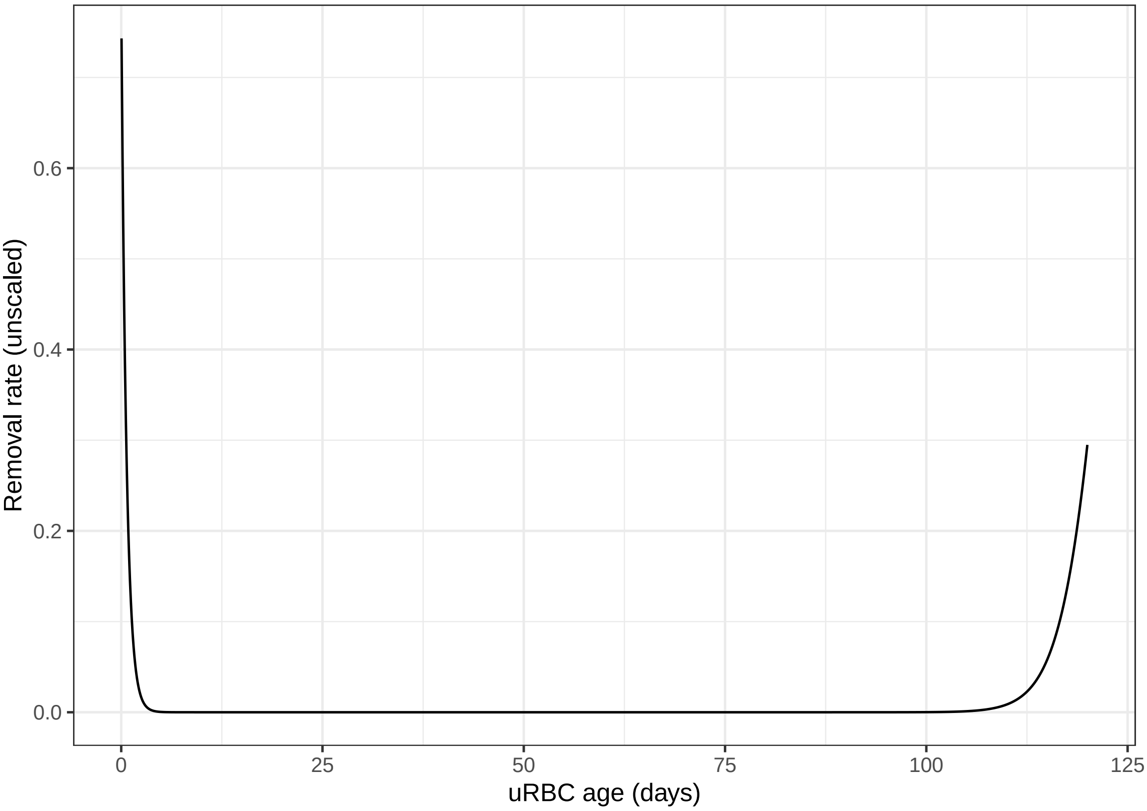

Figure G: The uRBC removal rate $\delta_u(a, t)$ from the circulation into the spleen when the scaling factor $F_U = 1$.

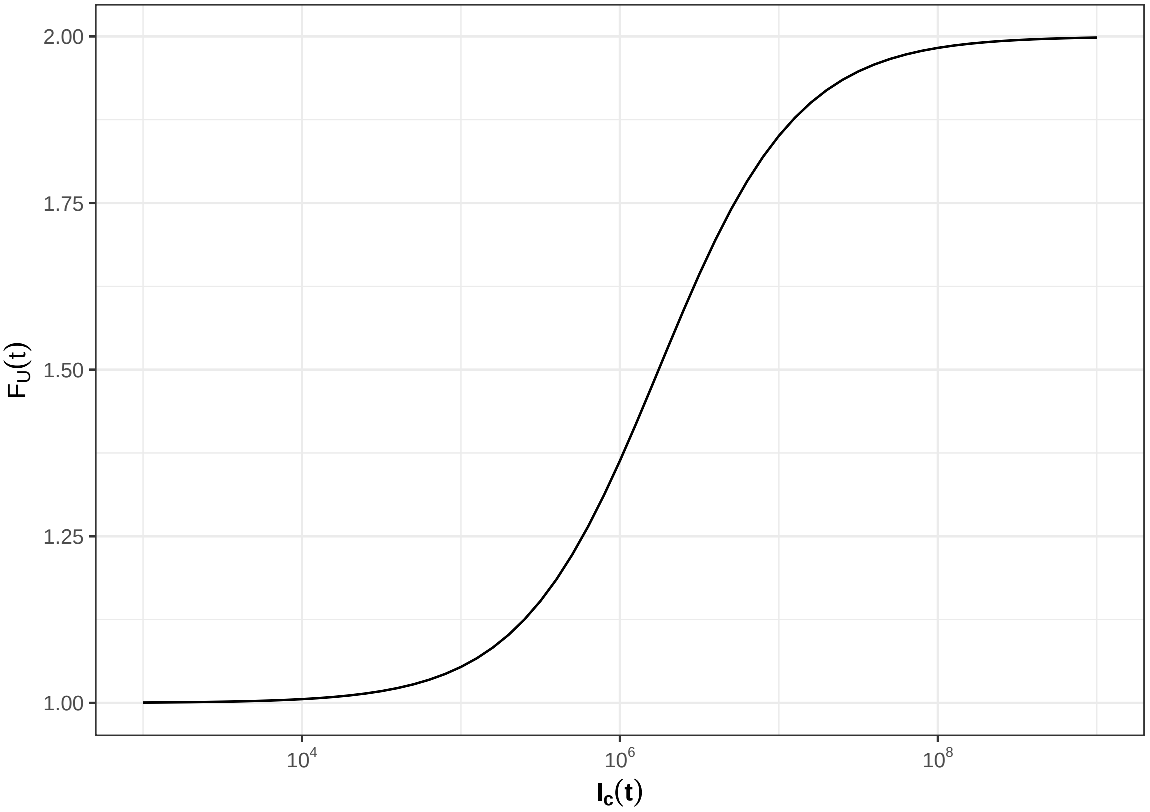

Figure H: The fold increase in uRBC removal rate due to the presence of iRBCs in the circulation, shown for $\mathbf{U_c}(t) = U_{ss}$.

6 Uninfected RBC return to circulation

Uninfected RBCs in the spleen return to the circulation at rate δ'_u, which is defined in terms of the log-normal probability density function F_X:

$$\delta'_u(a,t) = \text{mag} \cdot F_X(a) \tag{24}$$

$$\log(X) \sim \mathcal{N}(\mu = 24 \cdot \mu_U, \sigma = 24 \cdot \sigma_U) \tag{25}$$

Symbol	Description	Baseline value
mag	Scaling parameter	10
$_U$	Scaling parameter	3.65
$_U$	Scaling parameter	0.0025

We denote the number of uRBCs released in a time-step as $U_{s\to c}$:

$$U_{s\to c}(a,t) = U_s(a-1,t-1) \cdot \exp[-\lambda_u(a,t)] \cdot (1 - \exp[-\delta'_u(a)]) \tag{26}$$

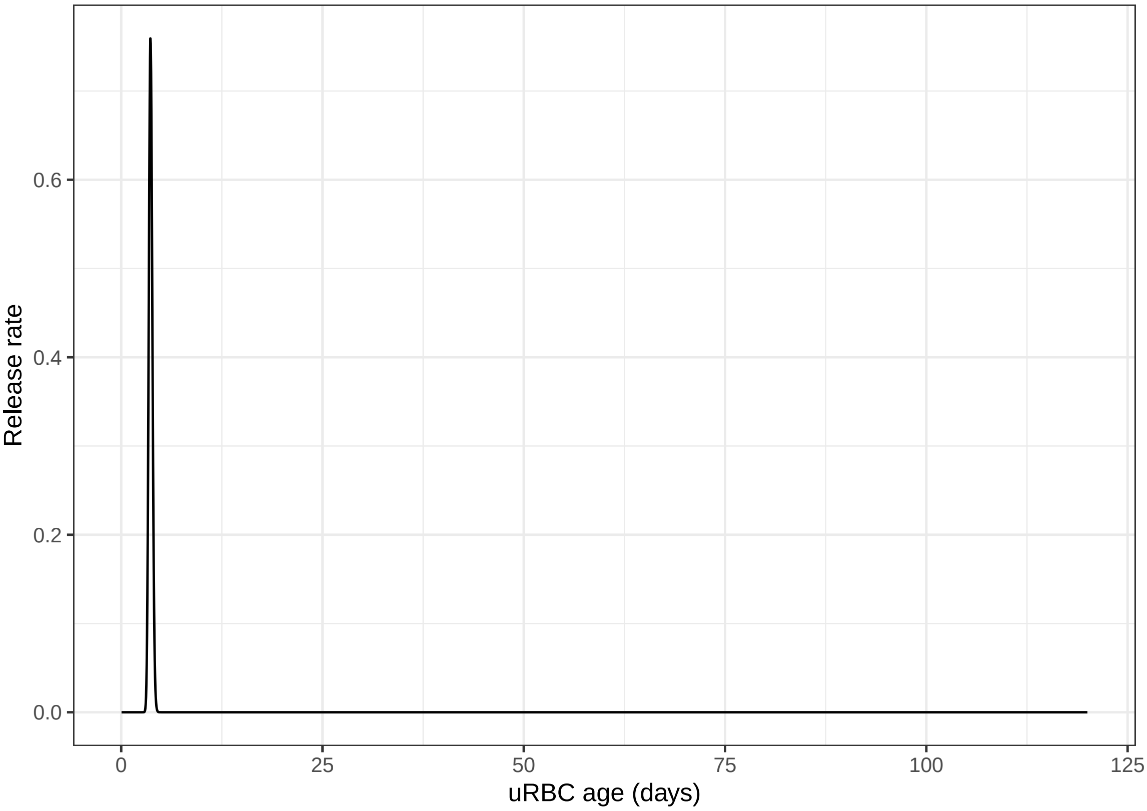

Figure I: The uRBC release rate $\delta'_u(a,t)$ from the spleen into the circulation.

7 RBC infection

Uninfected RBCs are infected at rates α_c and α_s in the circulation and spleen, respectively, which depend on the age-specific merozoite preference β:

$$\alpha_c(a,t) = \mathrm{PMF} \cdot \frac{\beta(a) \cdot U_c(a,t-1)}{\sum_{a'} \left[\beta(a') \cdot U_c(a',t-1)\right]} \tag{27}$$

$$\alpha_s(a,t) = \mathrm{PMF} \cdot \frac{\beta(a) \cdot U_s(a,t-1)}{\sum_{a'} \left[\beta(a') \cdot U_s(a',t-1)\right]} \tag{28}$$

Pf parasites invade RBCs of all ages, with some preference for younger RBCs:

$$\beta'(a) = \frac{a_\beta^{50} \cdot \exp\left[-5 \times 10^{-4} \cdot (a-1)\right]}{(a-1)^{\mathrm{sl}_\beta^{\mathrm{Pf}}} + (a_\beta^{50})^{\mathrm{sl}_\beta^{\mathrm{Pf}}}} \tag{29}$$

In contrast, Pv parasites only invade reticulocytes and prefer immature reticulocytes:

$$\beta'(a) = \begin{cases} 1 - \left[\mathrm{sl}_\beta^{\mathrm{Pv}} \cdot (a-1)\right]^{-1} & \text{if } a \leq T_M \\ 0 & \text{otherwise} \end{cases} \tag{30}$$

We normalise these age-specific preferences so that they sum to 1:

$$\beta(a) = \frac{\beta'(a)}{\sum_A \beta'(A)} \tag{31}$$

Merozoites are released when infected RBCs rupture at age T_{irbc}. Here we use the notation $\nabla_{x \to y}$ to define the number of uRBCs infected in location y by merozoites released in location x:

$$\nabla_{c \to c}(a,t) = \alpha_c(a,t) \cdot \left[I_c^\nabla + I_q(T_{\mathrm{irbc}}, t-1)\right] \tag{32}$$

$$\nabla_{s \to c}(a,t) = \alpha_c(a,t) \cdot \omega \cdot I_s^\nabla \tag{33}$$

$$\nabla_{s \to s}(a,t) = \alpha_s(a,t) \cdot (1-\omega) \cdot I_s^\nabla \tag{34}$$

These in turn are defined in terms of the iRBCs that remain in the circulation and spleen, respectively:

$$I_c^\nabla = I_c(T_{\mathrm{irbc}}, t-1) \cdot \exp[-\delta_i] \cdot \exp[-\zeta] \tag{35}$$

$$I_s^\nabla = I_s(T_{\mathrm{irbc}}, t-1) \cdot \exp[-\delta_i'] \cdot \exp[-\lambda_i] \tag{36}$$

We can then define the total number of infected RBCs in the circulation and in the spleen:

$$\nabla_c(t) = \sum_a \nabla_{c\to c}(a,t) + \sum_a \nabla_{s\to c}(a,t) \tag{37}$$

$$\nabla_s(t) = \sum_a \nabla_{s\to s}(a,t) \tag{38}$$

Symbol	Description	Baseline value
PMF	Parasite multiplication factor	8
ω	Proportion of merozoites released in the spleen that infect circulating uRBCS	0.1
$\mathrm{sl}_\beta^{\mathrm{Pf}}$	Age-specific slope parameter for Pf	20
a_β^{50}	Half-maximal age for Pf	80
$\mathrm{sl}_\beta^{\mathrm{Pv}}$	Age-specific slope parameter for Pv	4.5

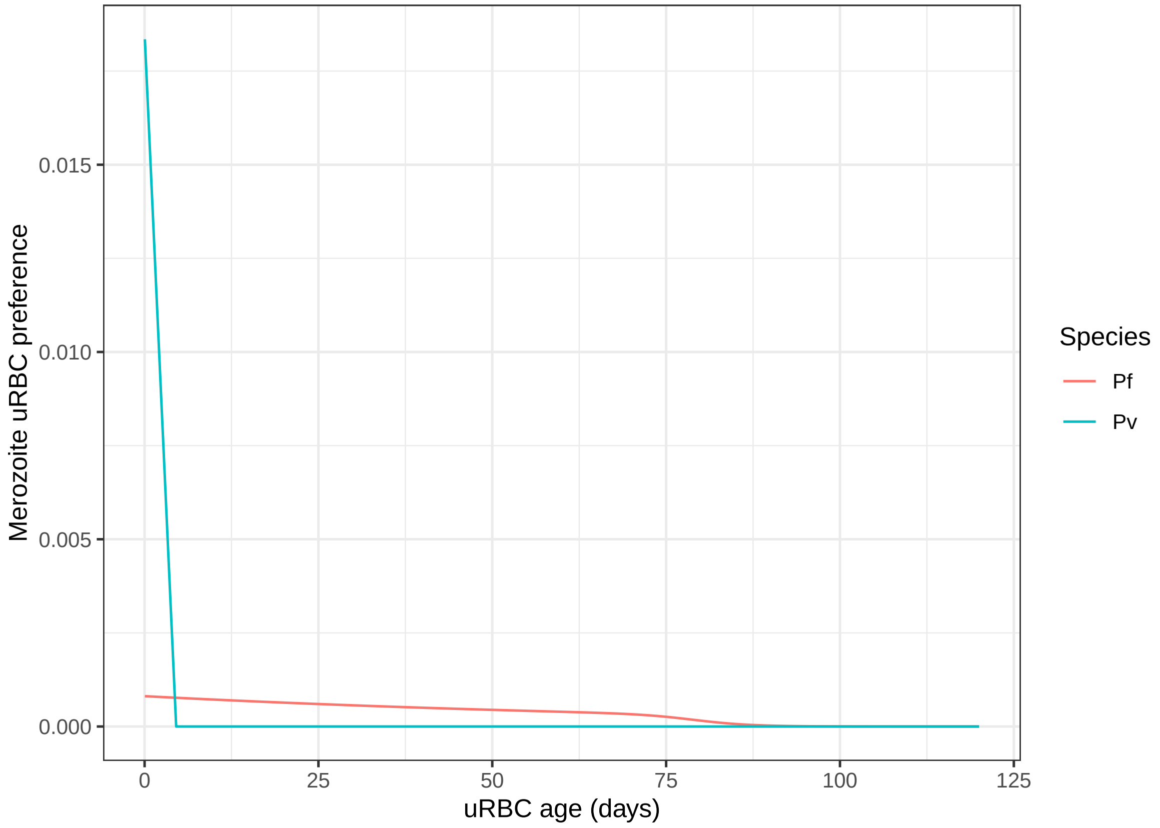

Figure J: The age-dependent merozoite preference for uninfected RBCs.

8 Infected RBC removal from circulation

The removal rate δ_i of infected RBCs from the circulation into the spleen depends on a number of offset, slope, and scaling parameters:

$$\delta_i(a,t) = F_I(t) \cdot \left[\delta_{iR} + (\delta_{iS} - \delta_{iR}) \cdot \frac{(a-1)^{\delta_I^{sl}}}{(a-1)^{\delta_I^{sl}} + (\delta_I^{c50})^{\delta_I^{sl}}} \right] \tag{39}$$

$$F_I(t) = 1 + k_\nu^I \cdot \frac{\mathbf{I_c}(t-1)^{g_d^U}}{\mathbf{I_c}(t-1)^{g_d^U} + \left(\delta_{50}^I \cdot [\mathbf{I_c}(t-1) + \mathbf{U_c}(t-1)]\right)^{g_d^U}} \tag{40}$$

$$\delta_{iS} = \delta_{iR} \cdot k_{iS} \tag{41}$$

Symbol	Description	Baseline value
k_{iS}	Schizont scaling parameter	2
δ_{iR}	**Pf** ring removal rate	0.562
δ_{iS}	**Pf** schizont removal rate	1.124
δ_{iR}	**Pv** ring removal rate	0.562
δ_{iS}	**Pv** schizont removal rate	1.124
δ_I^{sl}	Slope parameter	10
δ_I^{c50}	Half-maximal age	26
k_ν^I	Scaling parameter	3
g_d^U	Slope parameter	1
δ_{50}^I	Scaling parameter	10^{-4}

Safeukui et al. (2008) conducted in vitro experiments that showed 11% and 20% of Pf rings and schizonts, respectively, are retained in the spleen in every passage of the iRBCs through the spleen. Accordingly, we assume here that $\delta_{iS} \approx 2 \cdot \delta_{iR}$.

We denote the number of iRBCs removed in a time-step as $I_{c\to s}$:

$$I_{c\to s}(a,t) = I_c(a-1,t-1) \cdot (1 - \exp[-\delta_i(a,t)]) \tag{42}$$

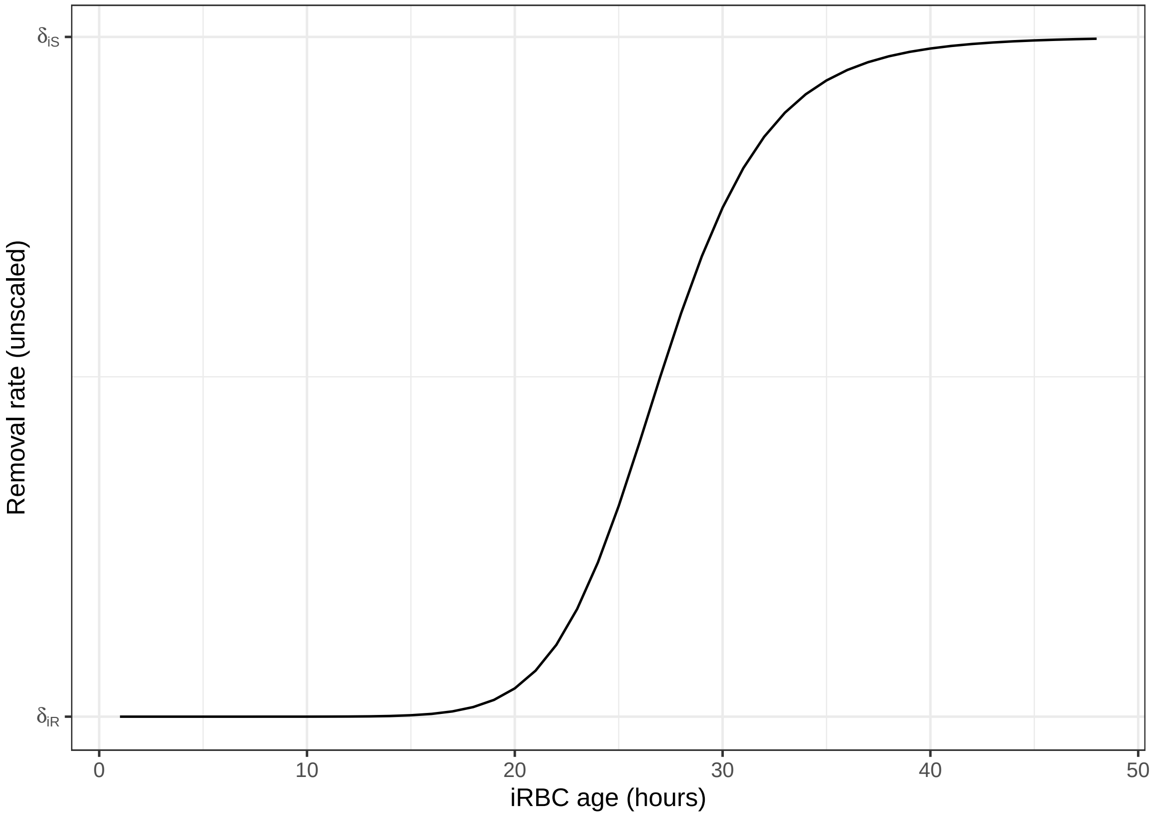

Figure K: The iRBC removal rate $\delta_i(a, t)$ from the circulation into the spleen when the scaling factor $F_I = 1$.

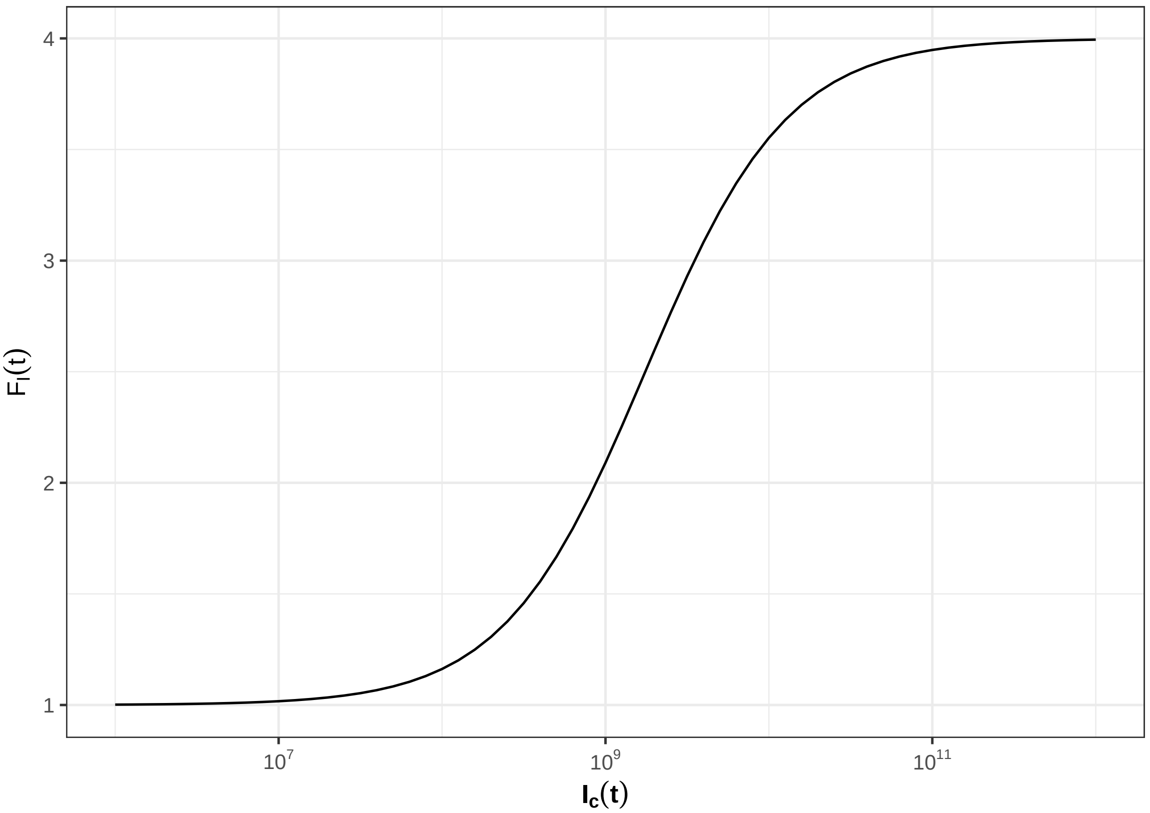

Figure L: The fold increase in iRBC removal rate due to the presence of iRBCs in the circulation, shown for $\mathbf{U_c}(t) = U_{ss}$.

9 Infected RBC return to circulation

Infected RBCs in the spleen return to the circulation at rate δ_i'. We assume that ring-stage RBCs are more likely to return to the circulation than are schizont-stage RBCs, and that infected RBCs are returned at a much lower rate than they are removed from the circulation into the spleen.

$$\delta_i'(a) = \delta_{iR}' + (\delta_{iS}' - \delta_{iR}') \cdot \frac{(a-1)^{\delta_I^{sl}}}{(a-1)^{\delta_I^{sl}} + (\delta_I^{c50})^{\delta_I^{sl}}} \tag{43}$$

$$\delta_{iR}' = \delta_{iR} \cdot k_{iR} \tag{44}$$

$$\delta_{iS}' = \delta_{iS} \cdot k_{iS} \tag{45}$$

Symbol	Description	Baseline value
k_{iR}	Scaling parameter	0.03
k_{iS}	Scaling parameter	0.01
δ_{iR}'	Ring iRBC return rate	0.01686
δ_{iS}'	Schizont iRBC return rate	0.01124
δ_I^{sl}	Slope parameter	10
δ_I^{c50}	Half-maximal age	26

We denote the number of iRBCs released in a time-step as $I_{s\to c}$:

$$I_{s\to c}(a,t) = I_s(a-1,t-1) \cdot \exp[-\lambda_i(a,t)] \cdot (1 - \exp[-\delta_i'(a)]) \tag{46}$$

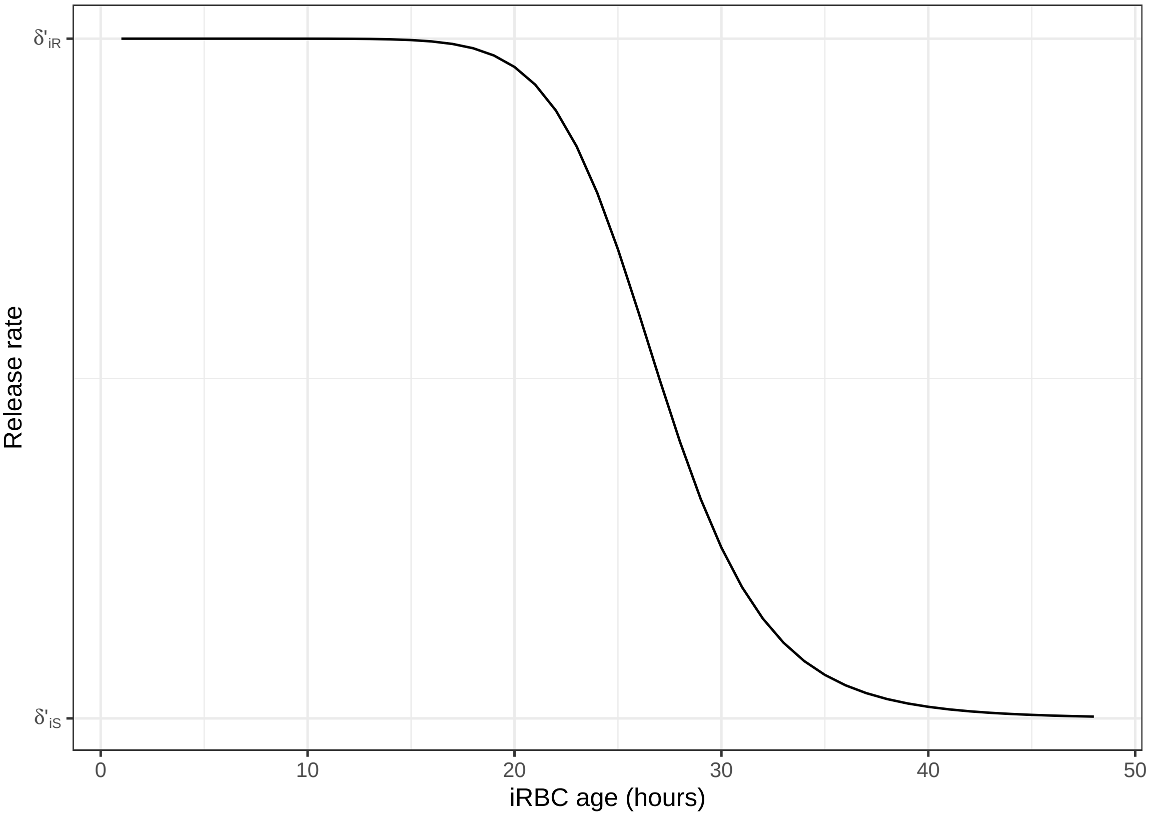

Figure M: The iRBC release rate $\delta_i'(a)$ from the spleen into the circulation.

10 Infected RBC sequestration

The sequestration rate depends on the parameters ζ_{sl} and ζ_{50}:

$$\zeta(a) = -\log(1 - 0.99) \cdot \frac{a^{\zeta_{\mathrm{sl}}}}{a^{\zeta_{\mathrm{sl}}} + \zeta_{50}^{\zeta_{\mathrm{sl}}}} \tag{47}$$

Circulating iRBCs are sequestered at rate $\zeta(a)$, which is very low for Pf rings, and increases with maturity. The maximum rate corresponds to a sequestration probability of 0.99.

We denote the number of iRBCs sequestered in a time-step as $I_{c\to q}$:

$$I_{c\to q}(a,t) = I_c(a-1,t-1) \cdot (1 - \exp[-\zeta(a)]) \tag{48}$$

$$I_q(a,t) = I_q(a-1,t-1) + I_{c\to q}(a,t) \tag{49}$$

Symbol	Description	Baseline value
ζ_{sl}	Slope parameter	10
ζ_{50}	Half-maximal age	26 hours

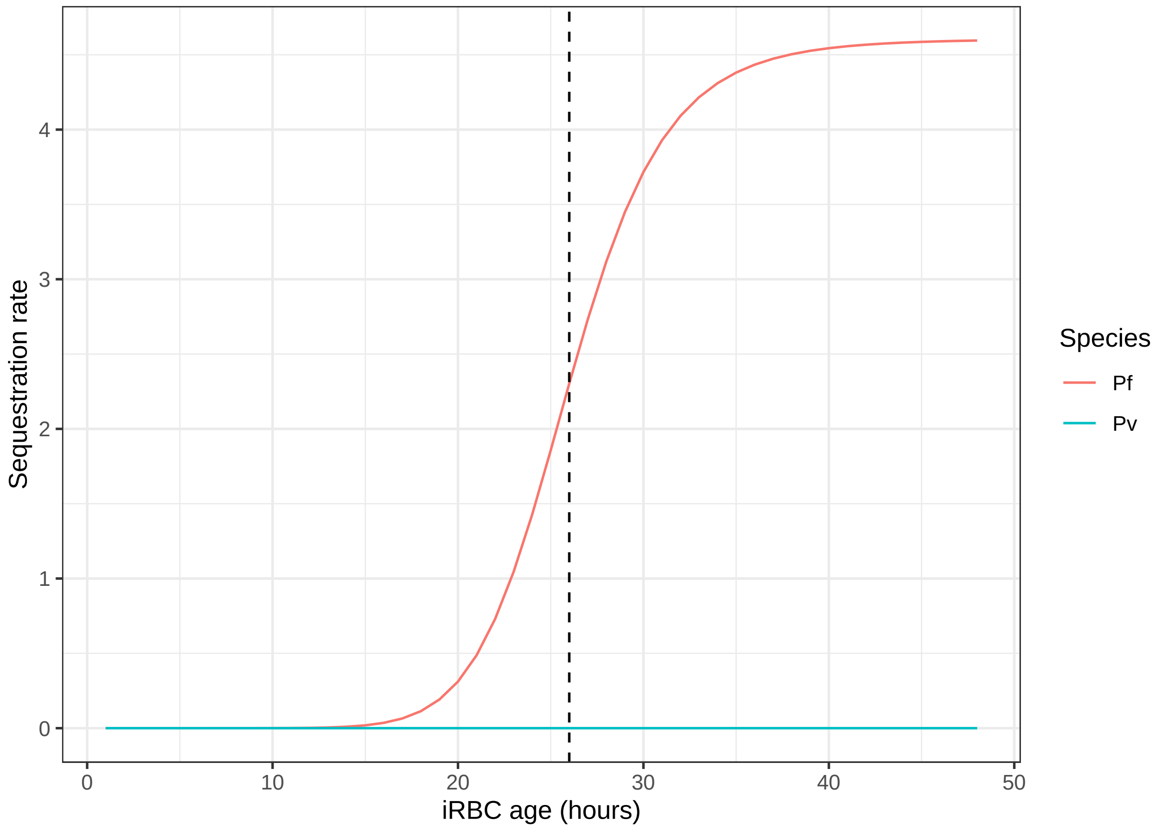

Figure N: The age-specific rate $\zeta(a)$ of iRBC sequestration.

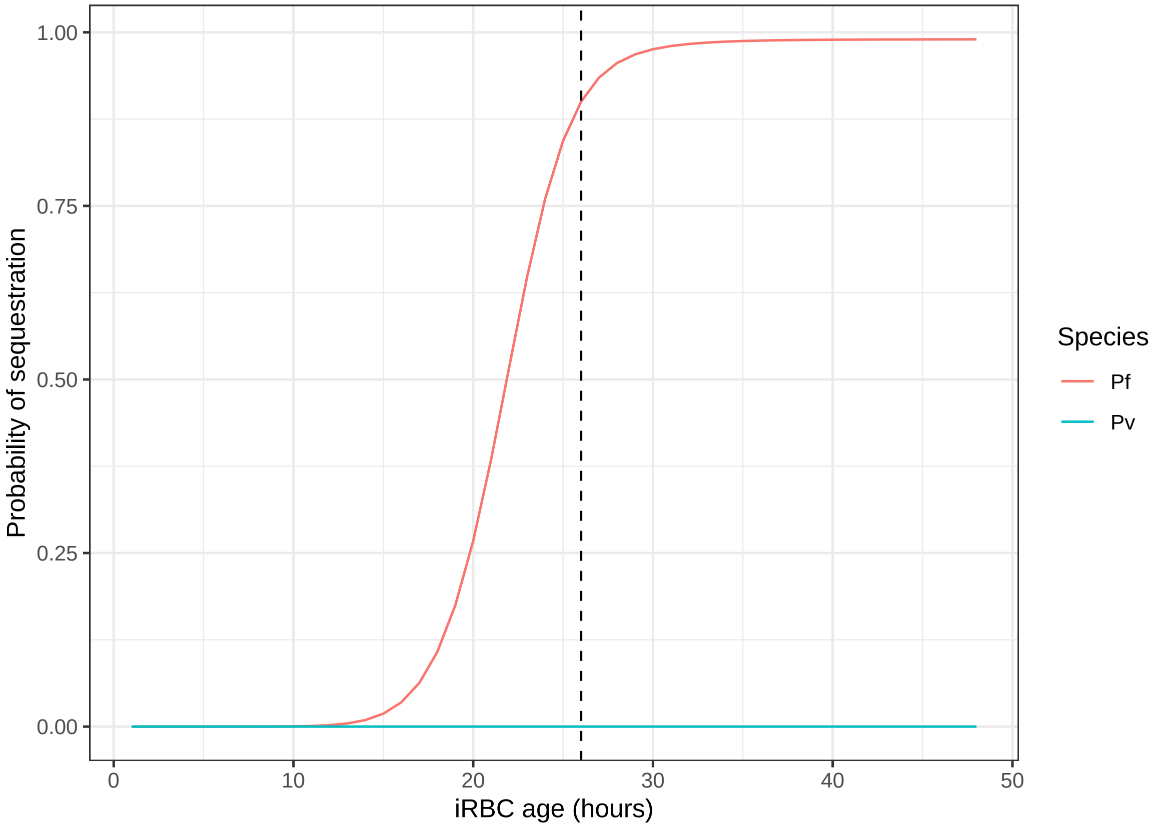

Figure O: The age-specific probability of iRBC sequestration.

11 RBC destruction in the spleen

Phagocytosis rates depend on the macrophage population $M(t)$ and rate parameters λ_u^M and λ_i^M:

$$\lambda_u(a,t) = \begin{cases} 0 & \text{for } a \leq T_M \\ \lambda_u^M \cdot M_u(t-1) & \text{for } a > T_M \end{cases} \tag{50}$$

$$\lambda_i(a,t) = \lambda_i^M \cdot M_i(t-1) \tag{51}$$

$$M_u(t) = M(t) \cdot \frac{\mathbf{U_s}(t-1)^{\gamma_M}}{\mathbf{U_s}(t-1)^{\gamma_M} + \mathbf{I_s}(t-1)^{\gamma_M}} \tag{52}$$

$$M_i(t) = M(t) \cdot \frac{\mathbf{I_s}(t-1)^{\gamma_M}}{\mathbf{U_s}(t-1)^{\gamma_M} + \mathbf{I_s}(t-1)^{\gamma_M}} \tag{53}$$

The macrophage population is defined with respect to the steady-state ratio of macrophages to RBCs retained in the spleen:

$$M(t) = k_M \left[b_M \left(\mathbf{U_s}(t-1) + \mathbf{I_s}(t-1) \right) - M(t-1) \right] + M(t-1) \tag{54}$$

$$b_M = \frac{M_0}{U_{r,ss}} \tag{55}$$

Table K: Model parameters for RBC destruction and macrophage populations.

Symbol	Description	Baseline value
λ_u^M	Rate parameter for uRBCs	5×10^{-7}
λ_i^M	Rate parameter for iRBCs	1.5×10^{-11}
k_M	Scaling factor	0.01
b_M	Steady-state ratio of M to U_s	0.0513979
γ_M	Scaling factor	0.25
T_M	Age at which reticulocytes mature	108 hours

12 Differences between Pf and Pv

- The two species have different age-dependent merozoite preferences for uninfected RBCs; see RBC infection for details.
- Pf-infected RBCs can become sequestered in the microvasculature; see Infected RBC sequestration for details.

13 Final RBC equations

We can now define the update rules for the RBC populations in the circulation and spleen, in terms of the equations defined above:

$$R_c(a,t) = \begin{cases} 0 & \text{for } a = 1 \\ R_{c\to c}(a,t) + U_{s\to c}(a,t) + r_{\to c}(a,t) - \nabla_{c\to c}(a,t) - \nabla_{s\to c}(a,t) & \text{for } 1 < a \leq T_M \end{cases} \tag{56}$$

$$N_c(a,t) = \begin{cases} R_{c\to c}(a,t) + U_{s\to c}(a,t) - \nabla_{c\to c}(a,t) - \nabla_{s\to c}(a,t) & \text{for } a = T_M + 1 \\ N_{c\to c}(a,t) + U_{s\to c}(a,t) - \nabla_{c\to c}(a,t) - \nabla_{s\to c}(a,t) & \text{for } T_M + 1 < a \leq T_U \end{cases} \tag{57}$$

$$U_c(a,t) = \begin{cases} R_c(a,t) & \text{for } 1 < a \leq T_R \\ N_c(a,t) & \text{for } T_R < a \leq T_U \end{cases} \tag{58}$$

$$U_s(a,t) = U_{s\to s}(a,t) + U_{c\to s}(a,t) - \nabla_{s\to s}(a,t) \tag{59}$$

$$I_c(a,t) = \begin{cases} \nabla_c(t) & \text{for } a = 1 \\ I_{c\to c}(a,t) + I_{s\to c}(a,t) & \text{for } 1 < a \leq T_I \end{cases} \tag{60}$$

$$I_s(a,t) = \begin{cases} \nabla_s(t) & \text{for } a = 1 \\ I_{s\to s}(a,t) + I_{c\to s}(a,t) & \text{for } 1 < a \leq T_I \end{cases} \tag{61}$$

where:

$$R_{c\to c}(a,t) = R_c(a-1,t-1) \cdot \exp[-\delta_u] \tag{62}$$

$$N_{c\to c}(a,t) = N_c(a-1,t-1) \cdot \exp[-\delta_u] \tag{63}$$

$$U_{s\to s}(a,t) = U_s(a-1,t-1) \cdot \exp[-\delta'_u - \lambda_u] \tag{64}$$

$$I_{c\to c}(a,t) = I_c(a-1,t-1) \cdot \exp[-\delta_i - \zeta] \tag{65}$$

$$I_{s\to s}(a,t) = I_c(a-1,t-1) \cdot \exp[-\delta'_i - \lambda_i] \tag{66}$$

14 Baseline outputs: no infection

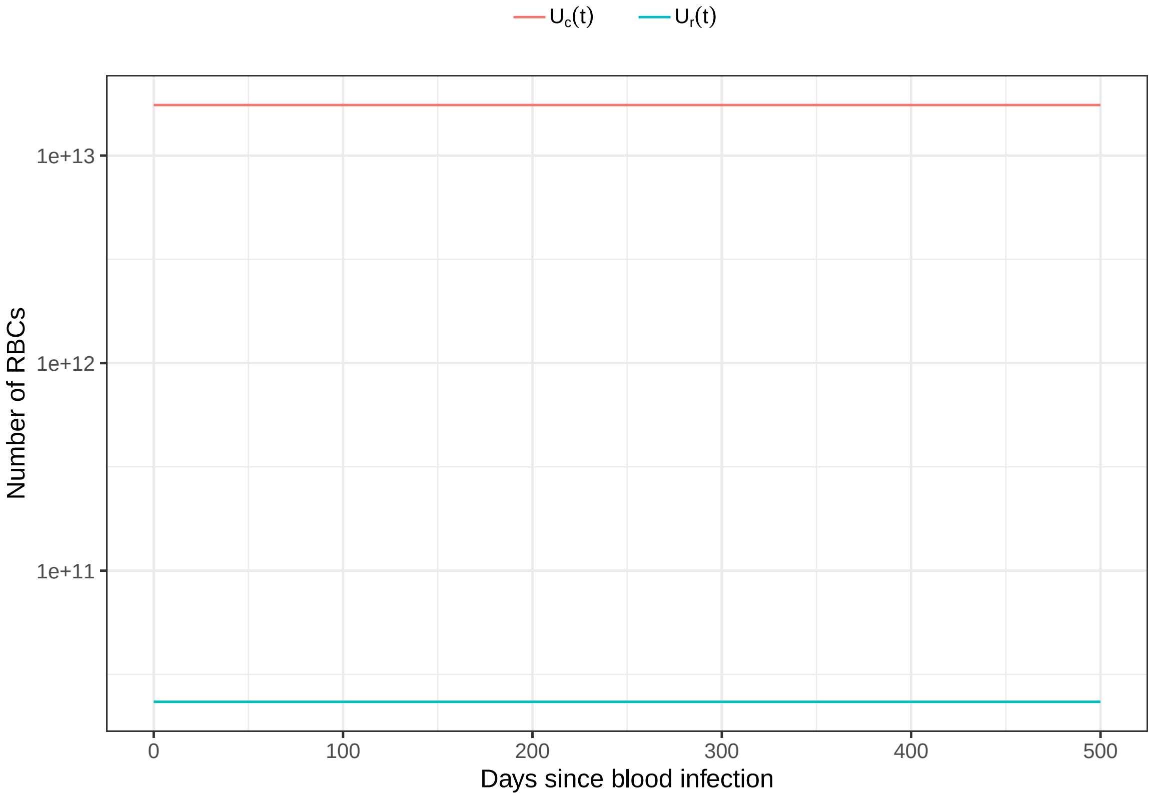

Figure P: RBC populations over time for the baseline parameter values (no infection).

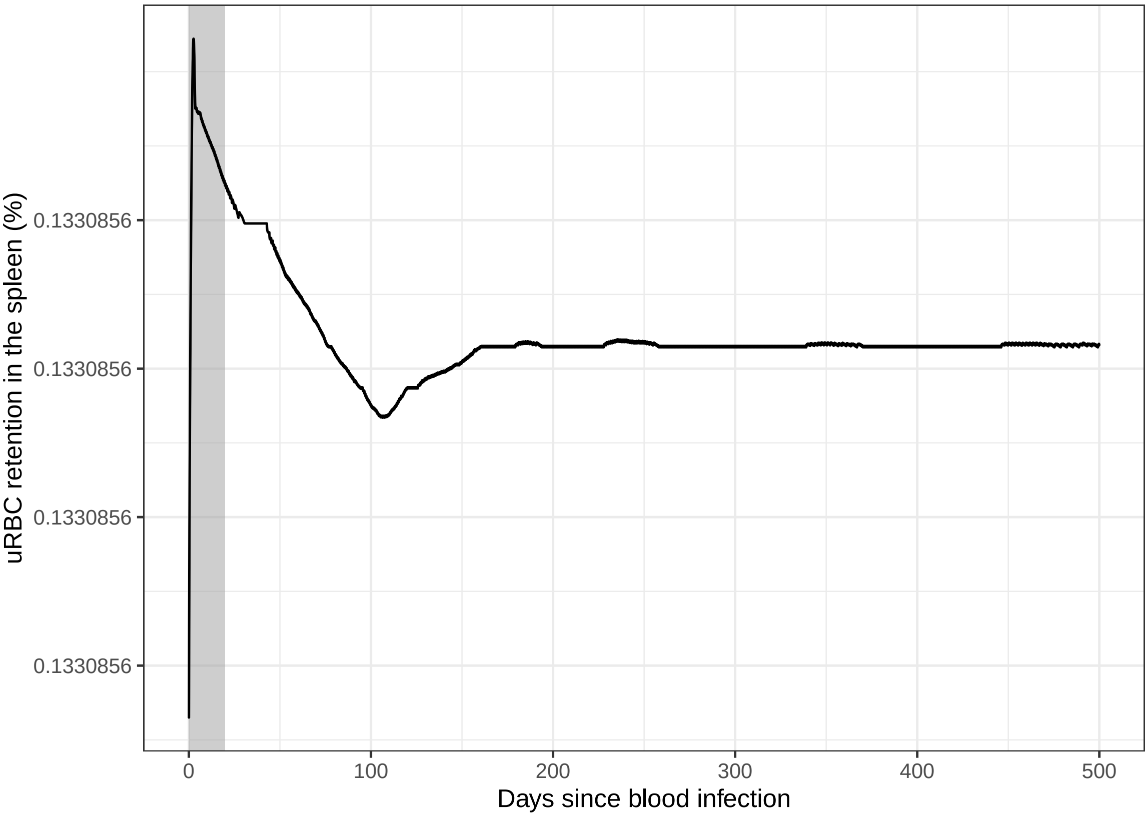

Figure Q: Uninfected RBC retention in the spleen (no infection).

15 Baseline outputs: Pf

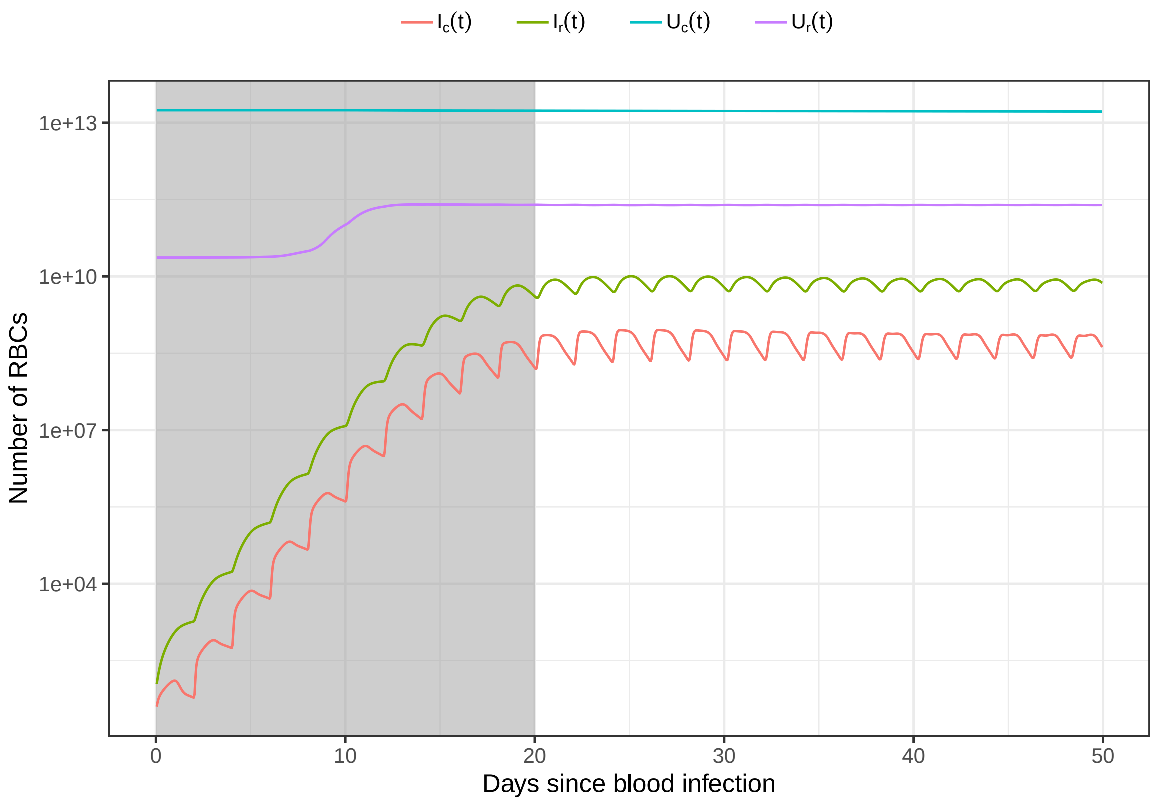

Figure R: RBC populations over time for the baseline parameter values (Pf infection).

```
#> [1] 11.08146
```

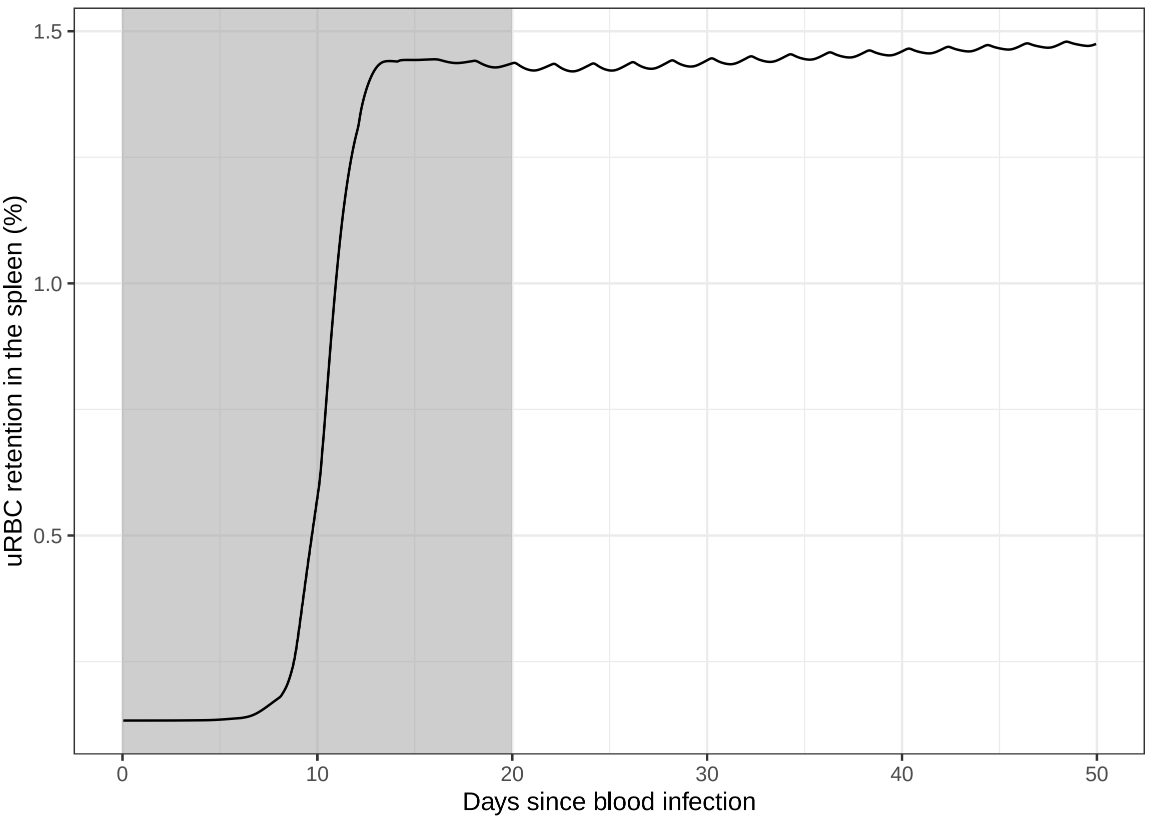

Figure S: Uninfected RBC retention in the spleen (Pf infection).

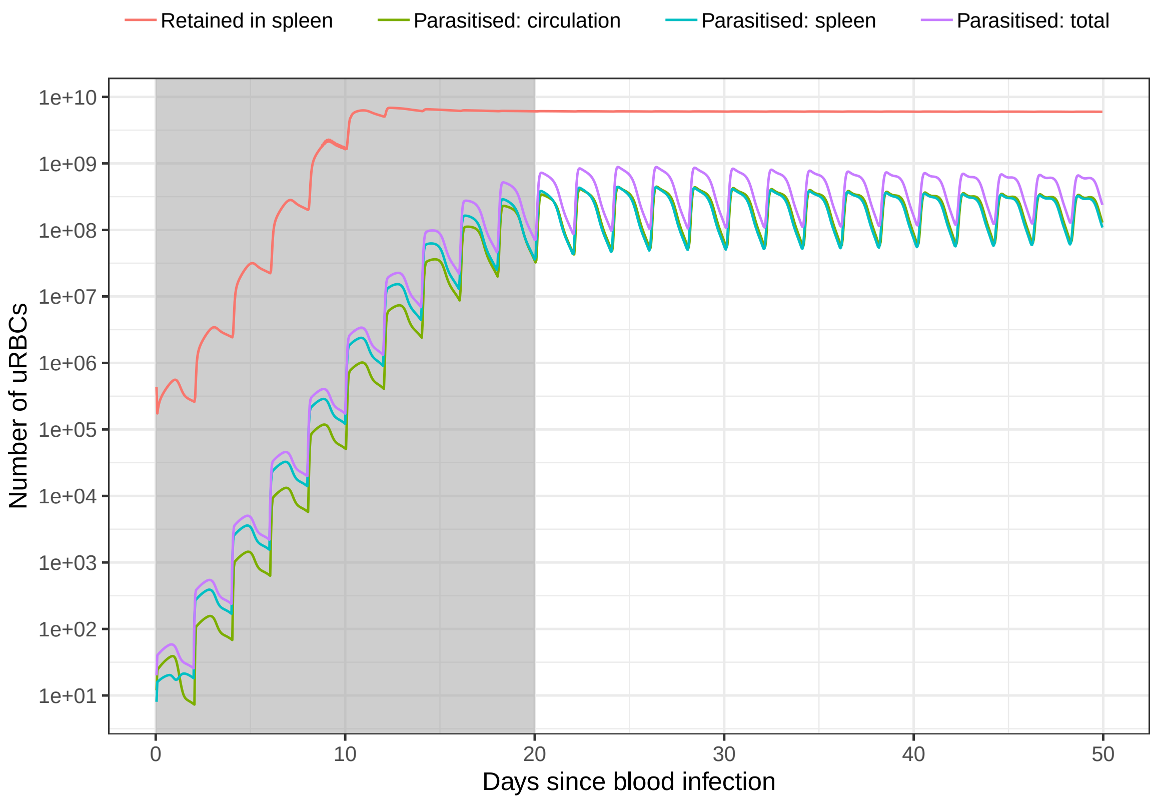

Figure T: Uninfected RBC loss due to malaria, by infection and by retention in the spleen (Pf infection). Splenic retention of uRBCs is substantially higher than parasitisation of circulating uRBCs at all stages of the infection. Shaded intervals indicate the acute infection phase (i.e., the transition from initial infection to chronic infection), which the model dynamics do not capture.

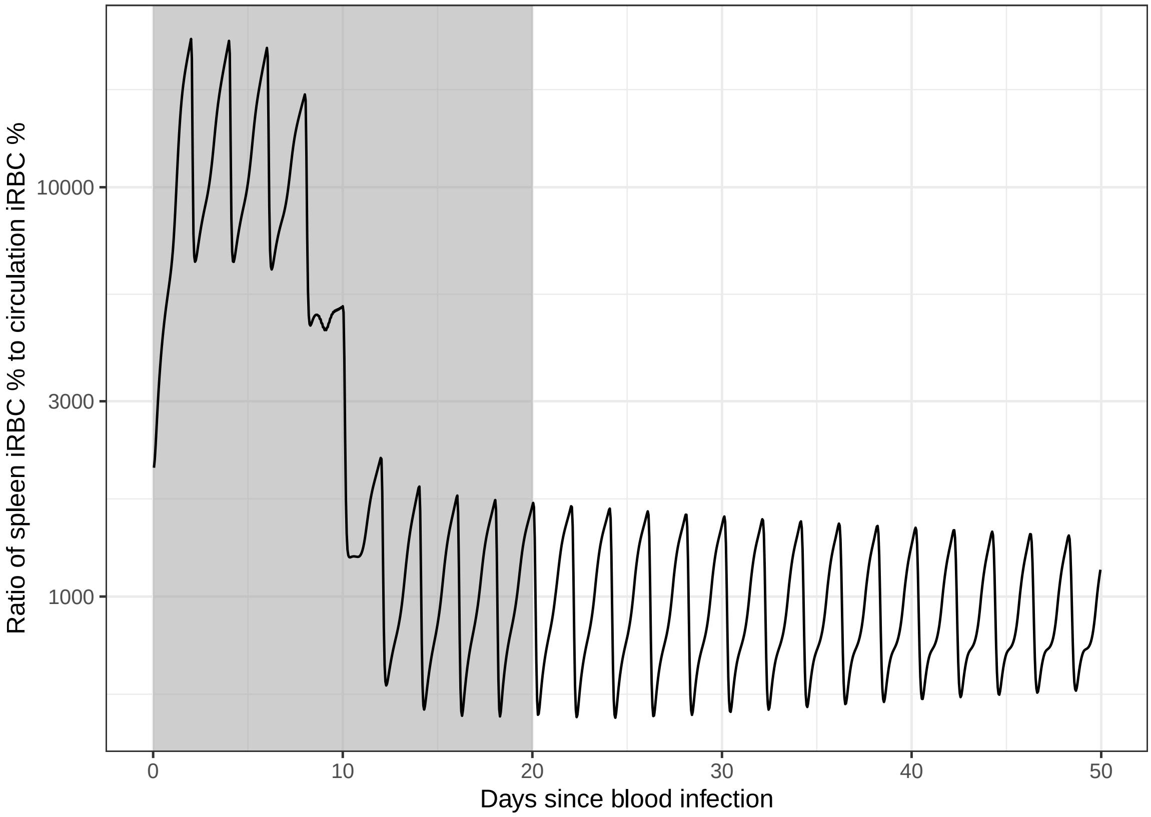

Figure U: The ratio of (a) the proportion of RBCs in the spleen that are infected; to (b) the proportion of RBCs in the circulation that are infected (Pf infection).

16 Baseline outputs: Pv

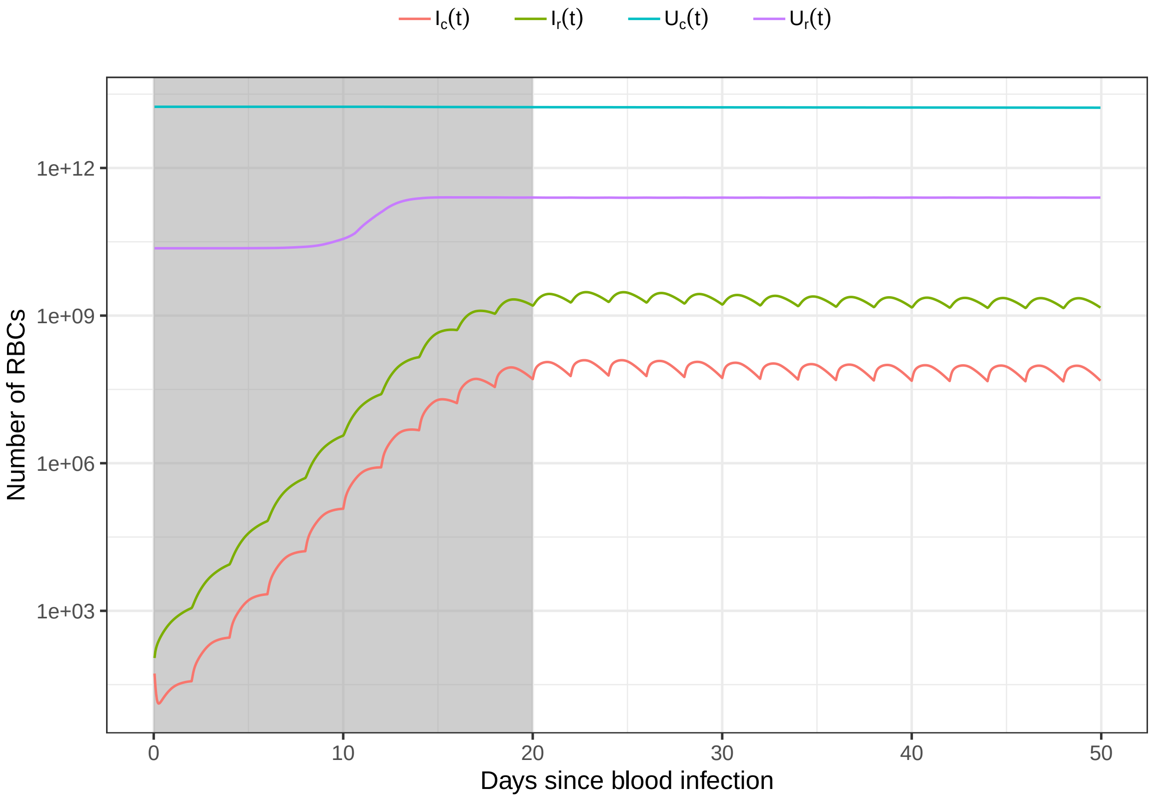

Figure V: RBC populations over time for the baseline parameter values (Pv infection).

```
#> [1] 11.0282
```

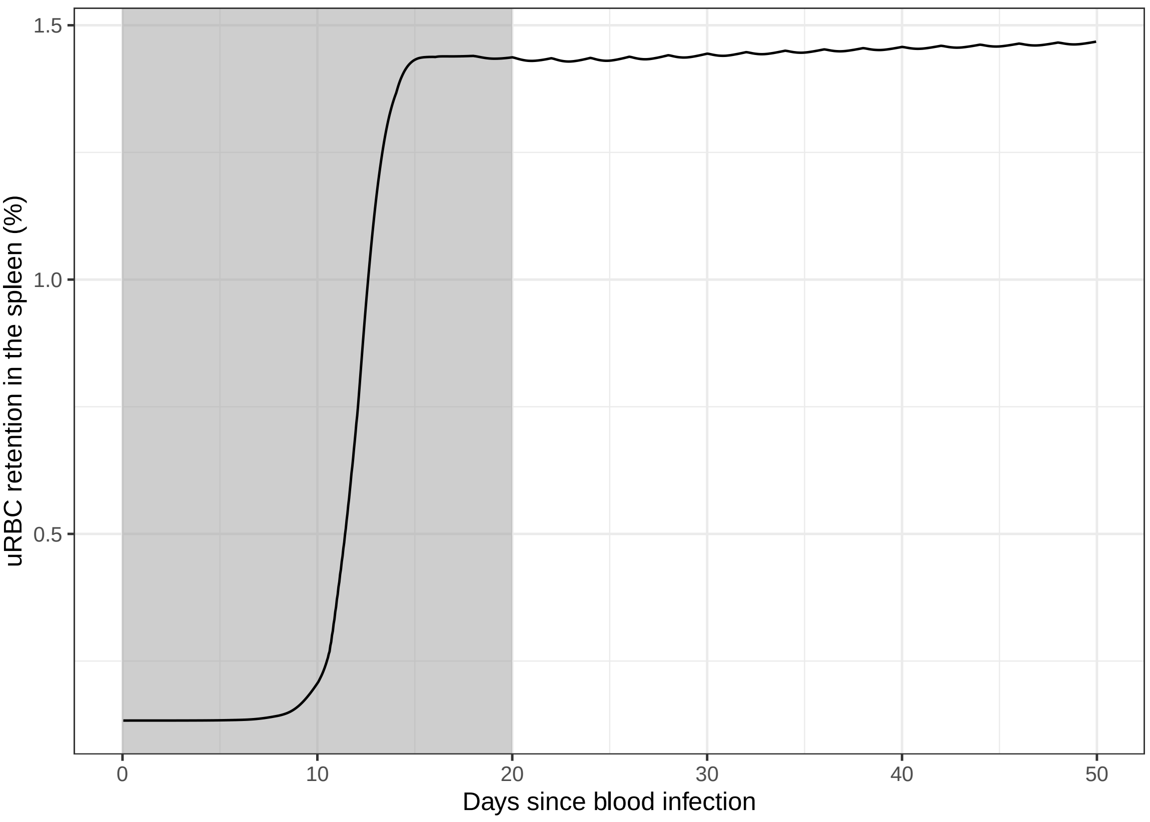

Figure W: Uninfected RBC retention in the spleen (Pv infection).

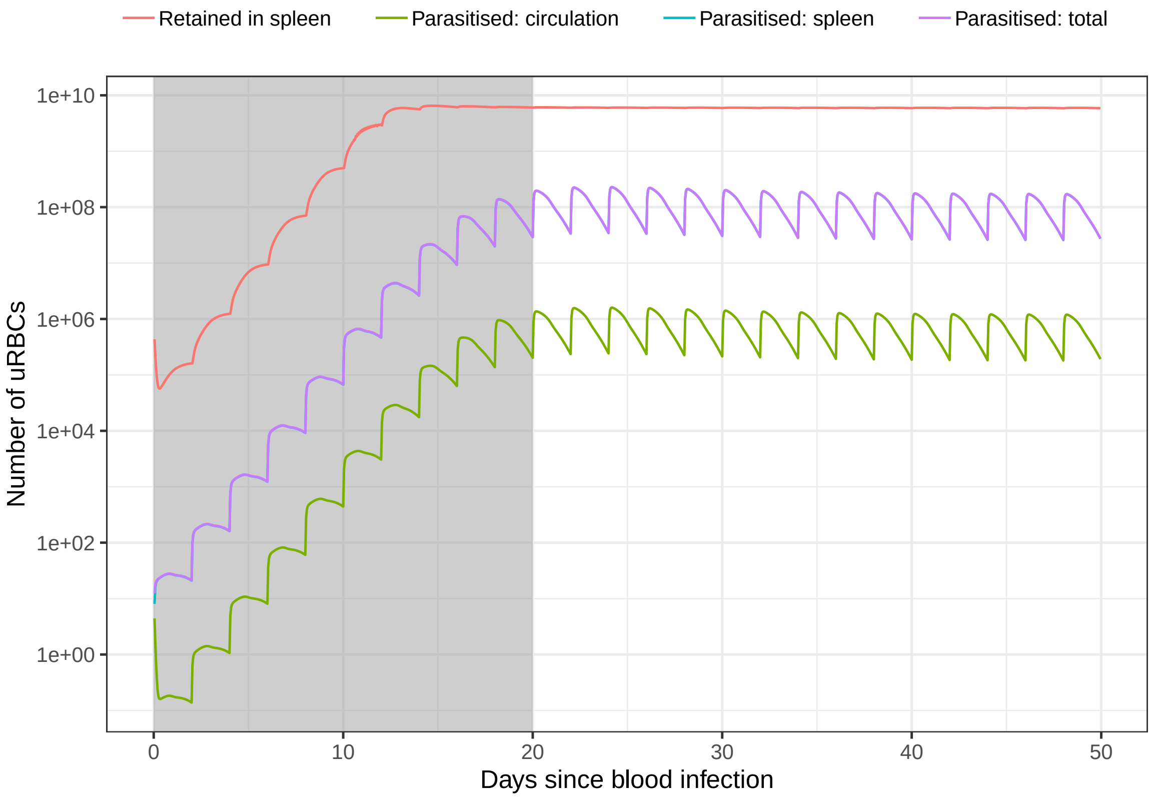

Figure X: Uninfected RBC loss due to malaria, by infection and by retention in the spleen (Pv infection). Splenic retention of uRBCs is substantially higher than parasitisation of circulating uRBCs at all stages of the infection. Shaded intervals indicate the acute infection phase (i.e., the transition from initial infection to chronic infection), which the model dynamics do not capture.

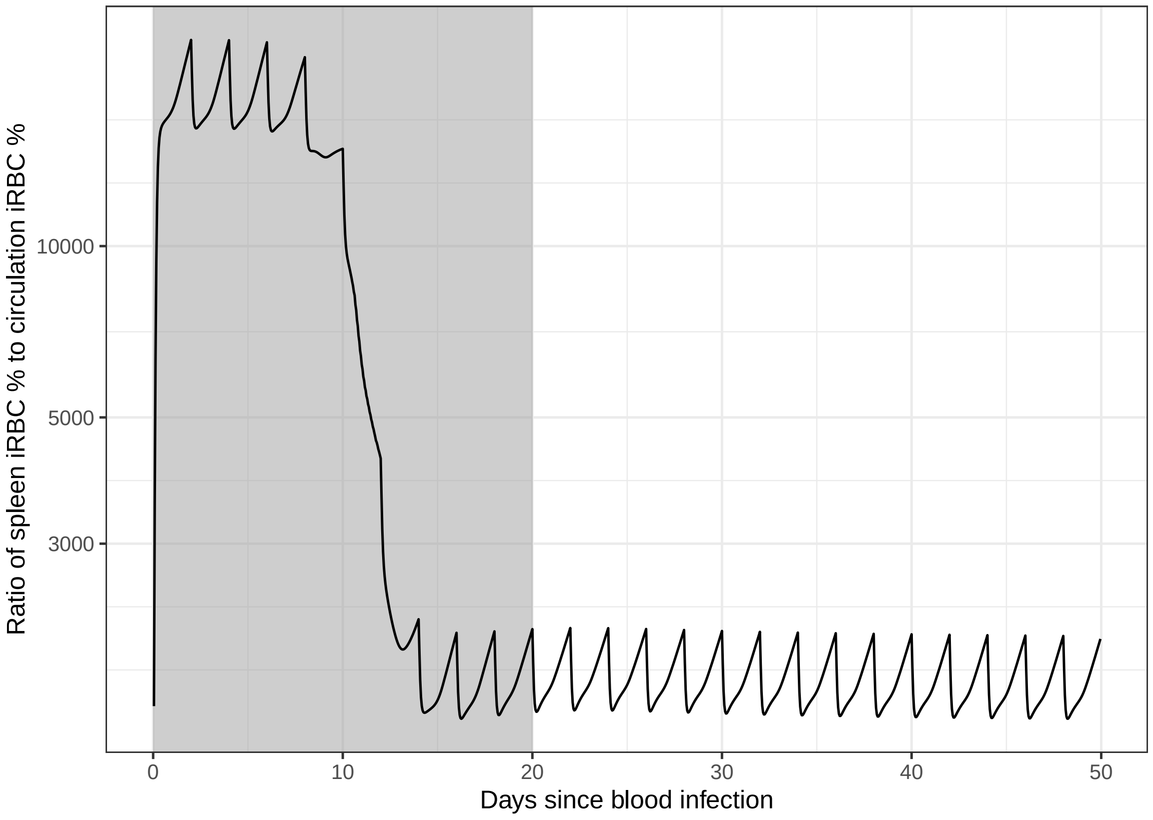

Figure Y: The ratio of (a) the proportion of RBCs in the spleen that are infected; to (b) the proportion of RBCs in the circulation that are infected (Pv infection).

17 Exploring Pv sequestration

We ran simulations that explored a range of Pv sequestration rates (defined relative to the Pf sequestration rate ζ), and observed that the iRBC biomass and RBC loss ratios both approached those obtained for Pf as the Pv sequestration rate approached the Pf sequestration rate.

Species	Sequestration	iRBC biomass	RBC loss
Pv	0%	1701:1	86:1
Pv	10%	1224:1	47:1
Pv	20%	1109:1	34:1
Pv	30%	1056:1	28:1
Pv	40%	1026:1	25:1
Pv	50%	1006:1	22:1
Pv	60%	988:1	21:1
Pv	70%	974:1	19:1
Pv	80%	962:1	18:1
Pv	90%	952:1	18:1
Pv	100%	943:1	17:1
Pf	100%	918:1	17:1

18 Model parameters

Process	Parameter	Value
Normoblast production	e_{sl}	16
	U_c^l	0.33
	$f_{\max}$	10
Reticulocyte release	ρ_0	0.001
	ρ_s	10
	ρ_i	0.5
	$T_R^{\min}$	24
	κ	10^{-9}
uRBC removal	δ_U^A	0.74303
	$\delta_U^{\min}$	2.16405×10^{-5}
	$\delta_U^{\max}$	1.206914
	δ_U^{c50}	2954.306
	δ_U^g	43.73335
	ν	0
	k_ν^U	1
	g_d^U	1
	δ_{50}^U	10^{-7}
uRBC release	mag	10
	μ_U	3.65
	σ_U	0.0025
RBC infection	ω	0.1
	PMF	8
	$\mathrm{sl}_\beta^{\mathrm{Pf}}$	20
	a_β^{50}	80
	$\mathrm{sl}_\beta^{\mathrm{Pv}}$	4.5
iRBC removal	$\delta_i R$ (Pf)	0.562
	$\delta_i S$ (Pf)	1.124
	$\delta_i R$ (Pv)	0.562
	$\delta_i S$ (Pv)	1.124
	δ_I^{sl}	10
	δ_I^{c50}	26
	k_ν^I	3
	δ_{50}^I	10^{-4}
iRBC release	k_{iR}	0.03
	k_{iS}	0.01
	δ'_{iR}	0.01686
	δ'_{iS}	0.01124
iRBC sequestration	ζ_{sl}	10
	ζ_{50}	26
RBC destruction	λ_u^M	5×10^{-7}
	λ_i^M	1.5×10^{-11}
	k_M	0.01
	b_M	0.0513979
	γ_M	0.25

Process	Parameter	Value
	T_M	108

References

Koepke, J.F., Koepke, J.A., 1986. Reticulocytes. Clinical & Laboratory Haematology 8, 169–179. https://doi.org/10.1111/j.1365-2257.1986.tb00093.x

Safeukui, I., Correas, J.-M., Brousse, V., Hirt, D., Deplaine, G., Mulé, S., Lesurtel, M., Goasguen, N., Sauvanet, A., Couvelard, A., Kerneis, S., Khun, H., Vigan-Womas, I., Ottone, C., Molina, T.J., Tréluyer, J.-M., Mercereau-Puijalon, O., Milon, G., David, P.H., Buffet, P.A., 2008. Retention of plasmodium falciparum ring-infected erythrocytes in the slow, open microcirculation of the human spleen. Blood 112, 2520–2528. https://doi.org/10.1182/blood-2008-03-146779

Watson, J., Taylor, W.R., Menard, D., Kheng, S., White, N.J., 2017. Modelling primaquine-induced haemolysis in G6PD deficiency. eLife 6. https://doi.org/10.7554/elife.23061
