# Supplementary material for: The role of the spleen in red blood cell loss caused by malaria: A mathematical model
Source: PLoS Comput Biol. 2026 Jan 12;22(1):e1013865. doi: 10.1371/journal.pcbi.1013865 (PMC12810929; doi:10.1371/journal.pcbi.1013865)
Supplement: S2 File — Presents the results of our sensitivity analysis. (PDF) [file pcbi.1013865.s002.pdf]

Sensitivity analyses for Pf and Pv

Contents

1 Overview **1**

1.1 Sampling distributions 1

1.2 Quantities of interest 6

1.3 Prior predictive intervals 6

1.4 Rank correlations 7

1.5 Partial rank correlations 9

1.6 Relative errors 9

1.7 Varying all but 2 parameters 10

References **10**

1 Overview

We assessed the model sensitivity to each parameter, and to the initial RBC count U_{ss}, by:

- Defining a distribution for each parameter and U_{ss};
- Drawing 1000 parameter combinations from these distributions (using Latin hypercube sampling);
- Running Pf and Pv model simulations for each parameter combination;
- Calculating prior predictive intervals for model outputs such as uninfected and infected RBC populations in the circulation and spleen; and
- Calculating rank correlation coefficients between (a) each parameter and U_{ss}; and (b) chronic infection steady-state outputs.

1.1 Sampling distributions

We defined log-uniform distributions $\mathcal{U}_{\log}(a, b)$ for parameters whose values might plausibly span multiple orders of magnitude, and defined uniform distributions $\mathcal{U}(a, b)$ for the remaining parameters. In the distributions listed below, we define the lower and upper bounds relative to the baseline value for each parameter.

1.1.1 Initial RBC count

We based this distribution on RBC counts from uninfected Papuans with no reported fever, as collected in a cross-sectional household survey (Pava et al., 2016), and used the 5%–95% interval:

$$U_{ss} \sim \mathcal{U}(1.15 \times 10^{13}, 2.36 \times 10^{13})$$

1.1.2 Normoblast production

The erythropoesis slope parameter e_{sl} has a baseline value of 16; we varied this by $\pm 50\%$:

$$e_{sl} \sim \mathcal{U}(50\%, 150\%)$$

The erythropoesis threshold parameter U_c^l is a fraction of the steady-state RBC count and has a baseline value of 0.33; we varied this by $\pm 50\%$:

$$U_c^l \sim \mathcal{U}(50\%, 150\%)$$

The maximum fold-increase parameters $f_{\max}$ has a baseline value of 10; we varied this by $\pm 20\%$:

$$f_{\max} \sim \mathcal{U}(80\%, 120\%)$$

1.1.3 Reticulocyte release

As described in the Model Description vignette, the baseline values for these parameters were obtained by calibrating the reticulocyte release curve to match experimental data (Koepke and Koepke, 1986).

The minimum release rate ρ_0 has a baseline value of 10^{-3}; we varied this 10-fold in each direction:

$$\rho_0 \sim \mathcal{U}_{\log}(10\%, 1000\%)$$

The slope parameter ρ_s for the reticulocyte release age has a baseline value of 10; we varied this by $\pm 50\%$:

$$\rho_s \sim \mathcal{U}(50\%, 150\%)$$

The inflection point ρ_i for the release age has a baseline value of 0.5; we varied this by ± 50:

$$\rho_i \sim \mathcal{U}(50\%, 150\%)$$

The scaling factor κ for the release rate has a baseline value 10^{-9}; we varied this by $\pm 50\%$:

$$\kappa \sim \mathcal{U}(50\%, 150\%)$$

1.1.4 uRBC removal

Note that the age-specific removal rate is a combination of an exponential curve (for young RBCs, <10 days old) and a sigmoid (for old RBCs, >100 days old).

The scaling parameter δ_U^A for the exponential decrease by RBC age has a baseline value of 0.74303, and the scaling parameters $\delta_U^{\min}$, $\delta_U^{\max}$, and δ_U^g for the sigmoid have baseline values of 2×10^{-5}, 1.2, and 43.7, respectively. We varied each of these parameters by $\pm 50\%$:

$$\begin{aligned}
\delta_U^A &\sim \mathcal{U}(50\%, 150\%) \\
\delta_U^{\min} &\sim \mathcal{U}(50\%, 150\%) \\
\delta_U^{\max} &\sim \mathcal{U}(50\%, 150\%) \\
\delta_U^g &\sim \mathcal{U}(50\%, 150\%)
\end{aligned}$$

The half-maximal age δ_U^{c50} for the sigmoid has a baseline value of 123 days; we varied this by $\pm$2days:

$$\delta_U^{c50} \sim \mathcal{U}(-2 \text{ days}, +2 \text{ days})$$

The maximum fold-increase parameter k_ν^U for uRBC removal rate has a baseline value of 1, which represents a maximum 2-fold increase in the removal rate; we defined a log-uniform distribution a mean of 1.5:

$$k_\nu^U \sim \mathcal{U}_{\log}(0.1, 5)$$

The scaling parameter δ_{50}^U for the contribution of retained uRBCs to this fold-increase has a baseline value of 10^{-7}; we varied this 100-fold in each direction:

$$\delta_{50}^U \sim \mathcal{U}_{\log}(1\%, 10000\%)$$

1.1.5 uRBC release

The scaling parameter mag for the maximum uRBC release rate has a baseline value of 10; we varied this 10-fold in each direction:

$$\text{mag} \sim \mathcal{U}_{\log}(10\%, 1000\%)$$

The mean age μ_U at which uRBCs are released has a baseline value of 3.65 days (i.e., maturing reticulocytes); we varied this by $\pm 20\%$:

$$\mu_U \sim \mathcal{U}(80\%, 120\%)$$

The standard deviation σ_U for the age at which uRBCs are released has a baseline value of (0.06 hours); we varied this by $\pm 50\%$:

$$\sigma_U \sim \mathcal{U}(50\%, 150\%)$$

1.1.6 RBC infection

The proportion ω of merozoites released in the spleen that infect circulating uRBCs has a baseline value of 0.1; we varied this by $\pm 100\%$:

$$\omega \sim \mathcal{U}(0\%, 200\%)$$

The parasite multiplication factor PMF has a baseline value of 8; we defined the bounds using the 90% prediction interval from Simpson et al. (2002):

$$\text{PMF} \sim \mathcal{U}(5.5, 12.3)$$

The age-specific merozoite preference for Pf is characterised by the slope parameter $\text{sl}_\beta^{\text{Pf}}$ and the half-maximal age a_β^{50}, which have baseline values of 20, and 80 days, respectively. We varied these parameters by ± 50 and ± 20 days, respectively:

$$\text{sl}_\beta^{\text{Pf}} \sim \mathcal{U}(50\%, 150\%) \quad (1)$$
$$a_\beta^{50} \sim \mathcal{U}(-20 \text{ days}, +20 \text{ days}) \quad (2)$$

The age-specific merozoite preference for Pf is characterised by the slope parameter $\text{sl}_\beta^{\text{Pv}}$, which has a baseline value of 4.5; we varied this by $\pm 50\%$:

$$\text{sl}_\beta^{\text{Pv}} \sim \mathcal{U}(50\%, 150\%)$$

1.1.7 iRBC removal

The removal rate δ_{iR} of ring iRBCs has a baseline value of 0.562; we varied this 10-fold in each direction:

$$\delta_{iR} \sim \mathcal{U}(10\%, 1000\%)$$

The removal rate δ_{iS} of schizont iRBCs, relative to ring iRBCs, has a baseline value of 2; we varied this by $\pm 50\%$:

$$\delta_{iS} \sim \delta_{iR} \times \mathcal{U}(50\%, 150\%)$$

The slope parameter δ_I^{sl} has a baseline value of 10; we varied this by $\pm 50\%$:

$$\delta_I^{sl} \sim \mathcal{U}(50\%, 150\%)$$

The half-maximal age δ_I^{c50} has a baseline value of 26 hours; we varied this by ± 12 hours, so that the maximum value was equal to the iRBC lifespan of 48 hours:

$$\delta_I^{c50} \sim \mathcal{U}(-12 \text{ hours}, +12 \text{ hours})$$

The maximum fold-increase parameter k_ν^I for iRBC removal rate has a baseline value of 3, which represents a maximum 4-fold increase in the removal rate; we defined a log-uniform distribution with a mean of 2.97 (approximately baseline) and absolute bounds:

$$k_\nu^I \sim \mathcal{U}_{\log}(0.1, 15)$$

The scaling parameter δ_{50}^I for the contribution of retained uRBCs to this fold-increase has a baseline value of 10^{-4}; we varied this 100-fold in each direction:

$$\delta_{50}^I \sim \mathcal{U}_{\log}(1\%, 10000\%)$$

1.1.8 iRBC release

The iRBC release rate parameters δ'_{iR} and δ'_{iS} are defined relative to the corresponding removal rate parameters δ_{iR} and δ_{iS}, via scaling parameters k_{iR} and k_{iS} with baseline values of 0.03 and 0.01, respectively. The release rate parameters δ'_{iR} and δ'_{iS} will therefore vary as we sample values for δ'_{iR} and δ'_{iS}, but we defined distributions for k_{iR} and k_{iS} to vary the relative scales of these removal and release rates. We varied each scaling parameter by $\pm 67\%$:

$$k_{iR} \sim \mathcal{U}(33\%, 167\%)$$
$$k_{iS} \sim \mathcal{U}(33\%, 167\%)$$

1.1.9 iRBC sequestration

The age ζ_{50} at which the sequestration rate is half-maximal has a baseline value of 26 hours; we varied this by ± 12 hours, so that the maximum value was equal to the iRBC lifespan of 48 hours:

$$\zeta_{50} \sim \mathcal{U}(-12 \text{ hours}, +12 \text{ hours})$$

The slope parameter for the sequestration rate ζ_{sl} has a baseline value of 10; we varied this by $\pm 90\%$:

$$\zeta_{sl} \sim \mathcal{U}(10\%, 190\%)$$

1.1.10 Phagocytosis

We varied the per-macrophage phagocytosis rates of uRBCs (λ_u^M) and iRBCs (λ_i^M) 10-fold in each direction:

$$\lambda_u^M \sim \mathcal{U}_{\log}(10\%, 1000\%)$$
$$\lambda_i^M \sim \mathcal{U}_{\log}(10\%, 1000\%)$$

The parameter kM controls how rapidly the macrophage population $M(t)$ changes in response to the quantity RBCs retained in the spleen; we varied this by $\pm 50\%$:

$$kM \sim \mathcal{U}(50\%, 150\%)$$

1.2 Quantities of interest

In addition to the uRBC and iRBC populations in the circulation, spleen, and microvasculature (Pf only), we also report the following model quantities:

- The ratio of iRBC biomass — the percentage of RBCs that are parasitised — (a) in the spleen; versus (b) in the circulation;
- The ratio of circulating uRBC loss due to (a) increased retention in the spleen; versus (b) infection by malaria parasites; and
- The ratio of uRBC retention — the percentage of uRBCs that are retained in the spleen — relative to the baseline value in the absence of a malaria infection.

1.3 Prior predictive intervals

We present the resulting prior predictive intervals for the uRBC and iRBC populations, and other quantities of interest, for a period of 150 days following the initial blood infection, for Pf infections (Figure A) and for Pv infections (Figure B). The uRBC and iRBC populations in the circulation and spleen are compared to the splenectomy patient data, which are shown as violin plots and red asterisks on the right-hand side of each plot. The circulating uRBC population is also compared to the circulating uRBC counts for uninfected Papuans, as collected in a cross-sectional household survey (Pava et al., 2016), shown as dashed violins on the left-hand side. For comparison, the model results for our baseline parameter values are also shown as yellow lines.

The intervals are sufficiently broad that they span the range of the splenectomy patient data, except for the highest recorded Pv splenic uRBC population. In particular, the model yields iRBC biomass ratios that span the full range of the splenectomy patient data.

The most striking difference between the intervals for Pf and Pv are:

- The splenic uRBC population is evenly distributed above and below the baseline results for Pf, but mostly lies below the baseline results for Pv;

- According, the uRBC retention ratio is evenly distributed above and below the baseline results of Pf, but mostly lies below the baseline results for Pv;
- In contrast, the iRBC biomass ratio mostly lies below the baseline results for Pf, and is more evenly distributed above and below the baseline results for Pv.

Since we have used identical parameter values for the Pf and Pv simulations, the only differences in the model between these two sets of results are:

- The different age-dependent merozoite preferences for uninfected uRBCs; and
- The ability for Pf-infected RBCs to become sequestered in the microvasculature, from where they can release merozoites into the circulation, but are protected from removal into the spleen.

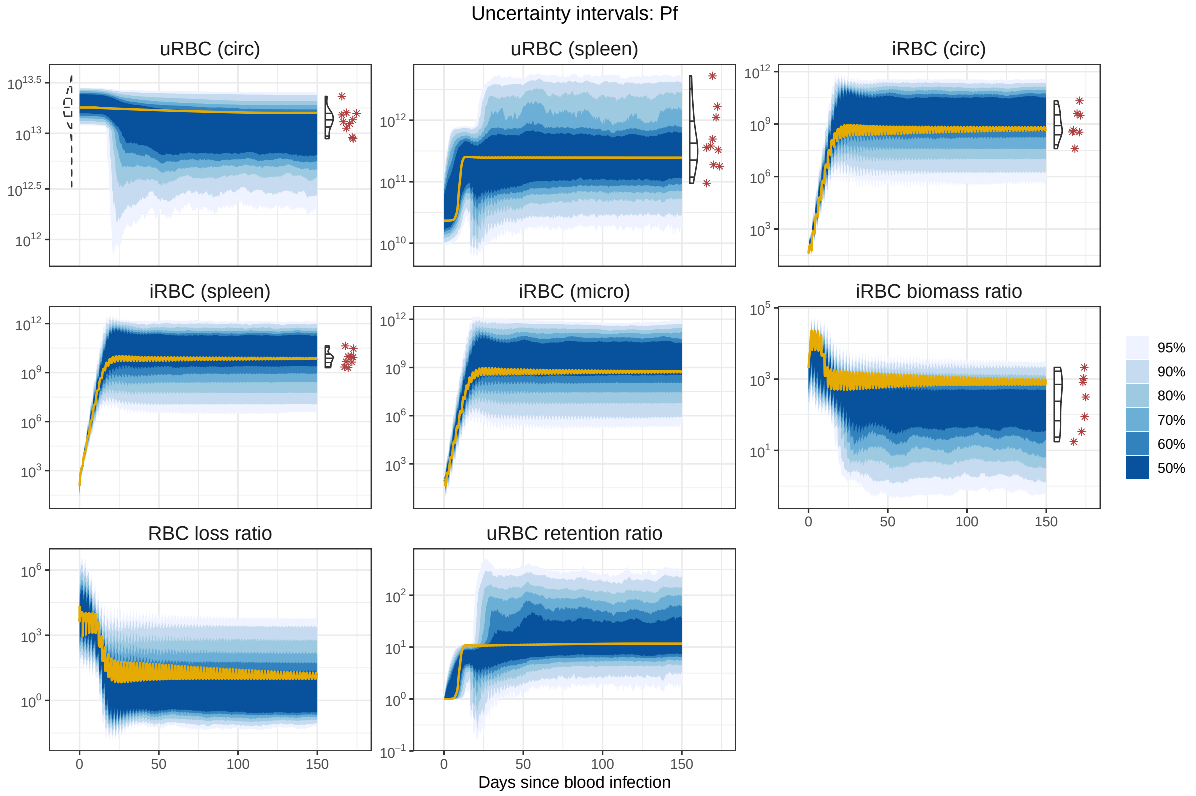

Figure A: Results for Pf infections. Prior predictive intervals are shown for the uninfected and infected RBC populations in the circulation, spleen, and microvasculature, and for the iRBC biomass ratio, RBC loss ratio, and uRBC retention rate. Yellow lines indicate the model results for baseline parameter values. Corresponding data in the splenectomised patients (Pf: n=9) are shown on the right of each plot for comparison (violin plots for distribution and red asterisks for observed data points). Dashed violins on the left indicate the circulating RBC counts for uninfected Papuans, as reported in a cross-sectional household survey.

1.4 Rank correlations

Spearman rank correlations between each model parameter and chronic infection steady-state outputs are shown in Figure C. The initial RBC count U_{ss} influences the circulating

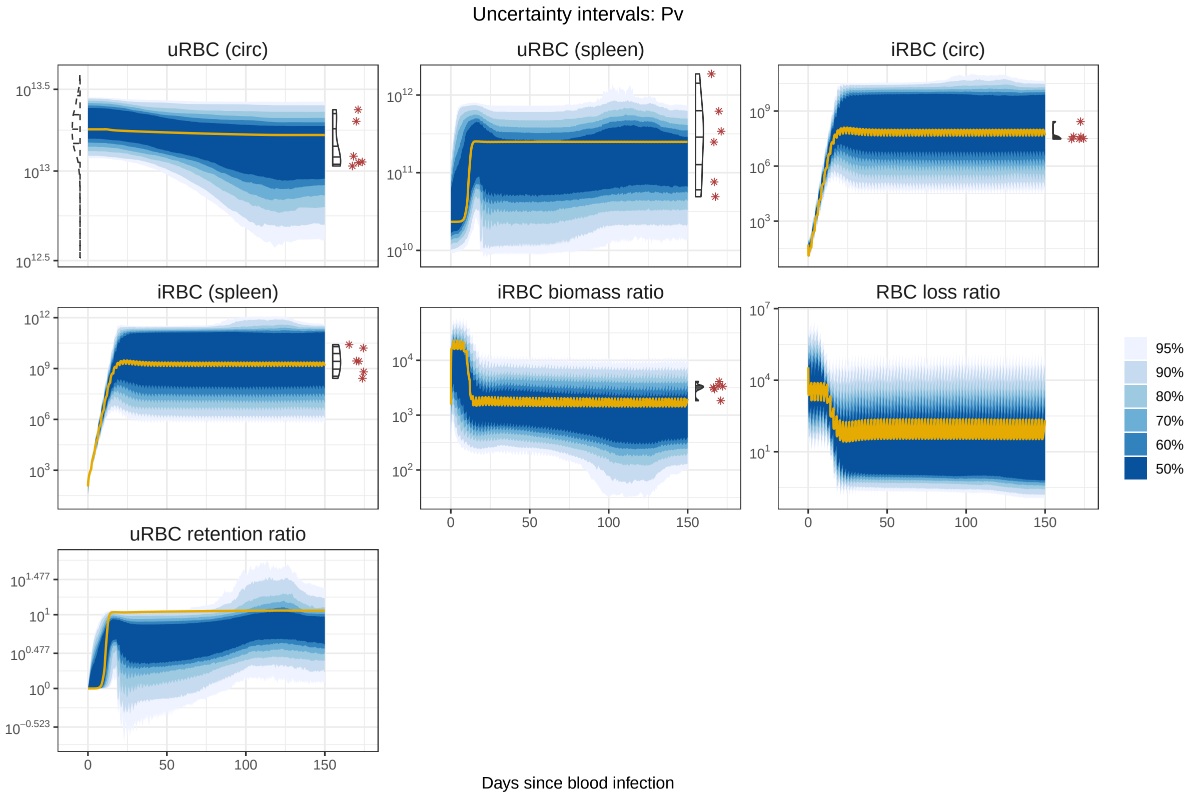

Figure B: Results for Pv infections. Prior predictive intervals are shown for the uninfected and infected RBC populations in the circulation and spleen, and for the iRBC biomass ratio, RBC loss ratio, and uRBC retention rate. Yellow lines indicate the model results for baseline parameter values. Corresponding data in the splenectomised patients (Pv: n=6) are shown on the right of each plot for comparison (violin plots for distribution and red asterisks for observed data points). Dashed violins on the left indicate the circulating RBC counts for uninfected Papuans, as reported in a cross-sectional household survey.

uRBC count and, to a lesser degree, the splenic uRBC count. Model parameters that have noticeable influence on the chronic infection steady-state include:

- The erythropoesis threshold parameter U_c^l also influences the circulating uRBC count.
- The minimum reticulocyte release rate ρ_0 (for reticulocytes younger than the release age) is negatively correlated with the circulating uRBC count and positively correlated with the splenic uRBC count and with the circulating and splenic iRBC counts. Recall that immature reticulocytes are removed into the spleen, where they are retained until they mature. In addition, the increased supply of reticulocytes provides both Pf and Pv merozoites more target RBCs to infect.
- The maximum fold-increase k_ν^U in the uRBC removal rate is positively correlated with the splenic macrophage population (which increases in proportion to the splenic RBC population) and with the circulating uRBC loss ratio and the uRBC retention ratio. It has minimal impact on the iRBC populations.
- The maximum fold-increase k_ν^I in the iRBC removal rate is positively correlated with the iRBC biomass ratio.
- The iRBC phagocytosis rate per individual macrophage λ_i^M has the largest absolute correlation with most of the model outputs. In particular, it is positively correlated with the circulating uRBC count, the circulating uRBC loss ratio, and the iRBC biomass ratio, and negatively correlated with the circulating and splenic iRBC populations, and with the splenic macrophage population.

Note that while the maximum fold-increase parameters k_ν^U and k_ν^I affect the splenic retention of uRBCs and iRBCs, respectively, the values of these parameters **are not equal** to the actual fold-increases in uRBC and iRBC retention.

1.5 Partial rank correlations

Spearman partial rank correlations between each model parameter and chronic infection steady-state outputs are shown in Figure D.

1.6 Relative errors

For each simulation in the sensitivity analysis, we calculate errors for the output variable $\hat{y}$ relative to the values $y = \{y_1, y_2, \dots\}$ reported in the patient cohort:

$$E(\hat{y}, y) = \frac{|\log(\hat{y}) - \log(\text{med}(y))|}{\log(\max(y)) - \log(\min(y))}$$

Figures E and F show local polynomial regression fits (`loess`) for the relative errors over each model parameter, for each of the following output variables:

- The uRBC populations in the circulation and spleen;
- The iRBC populations in the circulation and spleen;
- The iRBC count ratio (splenic iRBC population : circulating iRBC population); and
- The iRBC biomass ratio.

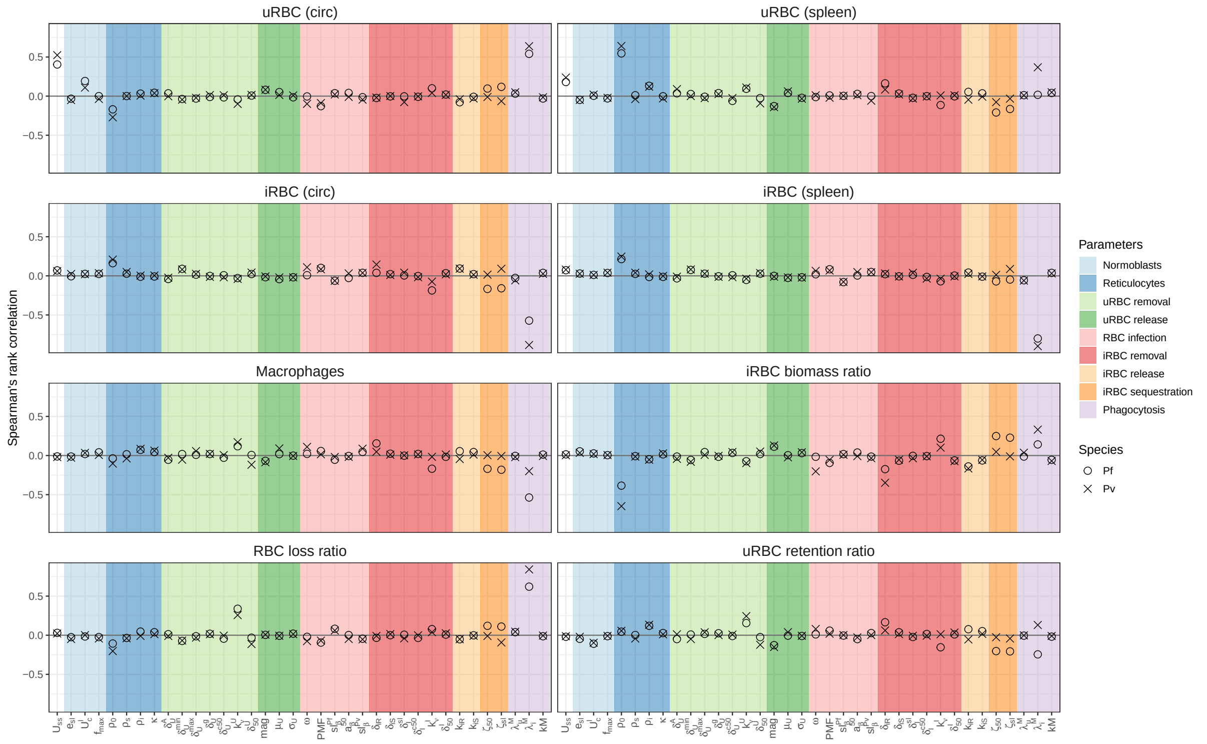

Figure C: Rank correlation coefficients between each model parameter and chronic infection steady-state outputs.

Figure G shows local polynomial regression fits for the net relative error (the sum of the relative errors for each output variables listed above).

For Pf the splenic iRBC population is consistently the source of the largest relative error, while for Pv the circulating iRBC population and iRBC biomass ratio are consistently the two largest relative errors and are responsible for the consistently larger net error observed in the Pv simulations, relative to the Pf simulations.

1.7 Varying all but 2 parameters

If we keep λ_i^M and δ_{iR} fixed at their baseline values and sample values for all other parameters from the sampling distributions defined above, we are able to obtain a better fit for Pv (a net error of 0.417, compared to 1.11 in the 2-parameter sweep) but this is achieved with several parameters taking values at the very extremes of the sampling distributions, including the RBC count at homeostasis (U_{ss}).

References

Koepke, J.F., Koepke, J.A., 1986. Reticulocytes. Clinical & Laboratory Haematology 8, 169–179. https://doi.org/10.1111/j.1365-2257.1986.tb00093.x

Pava, Z., Burdam, F.H., Handayuni, I., Trianty, L., Utami, R.A.S., Tirta, Y.K., Kenangalem, E., Lampah, D., Kusuma, A., Wirjanata, G., Kho, S., Simpson, J.A., Auburn, S., Douglas, N.M., Noviyanti, R., Anstey, N.M., Poespoprodjo, J.R., Marfurt, J., Price, R.N., 2016. Submicroscopic and asymptomatic Plasmodium parasitaemia asso-

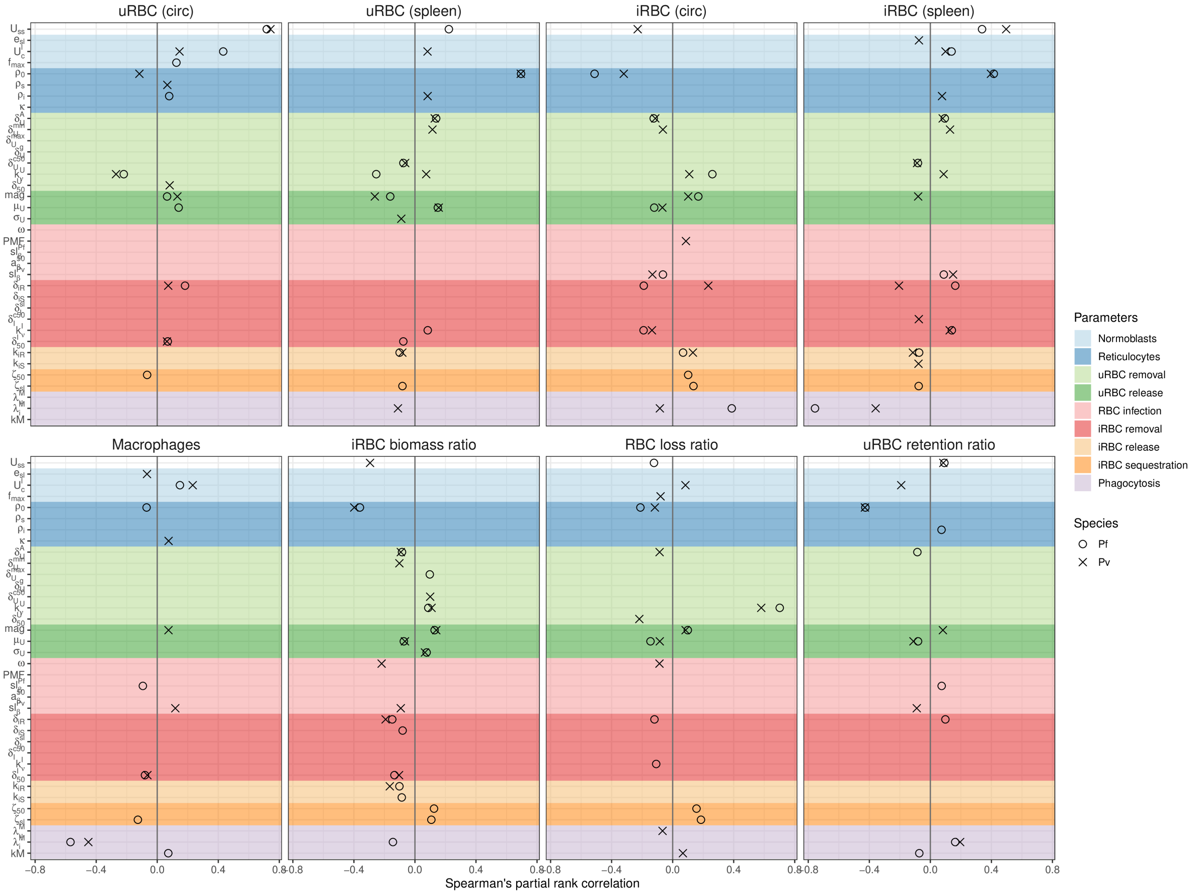

Figure D: Partial rank correlation coefficients between each model parameter and chronic infection steady-state outputs, showing only those coefficients where $p < 0.05$.

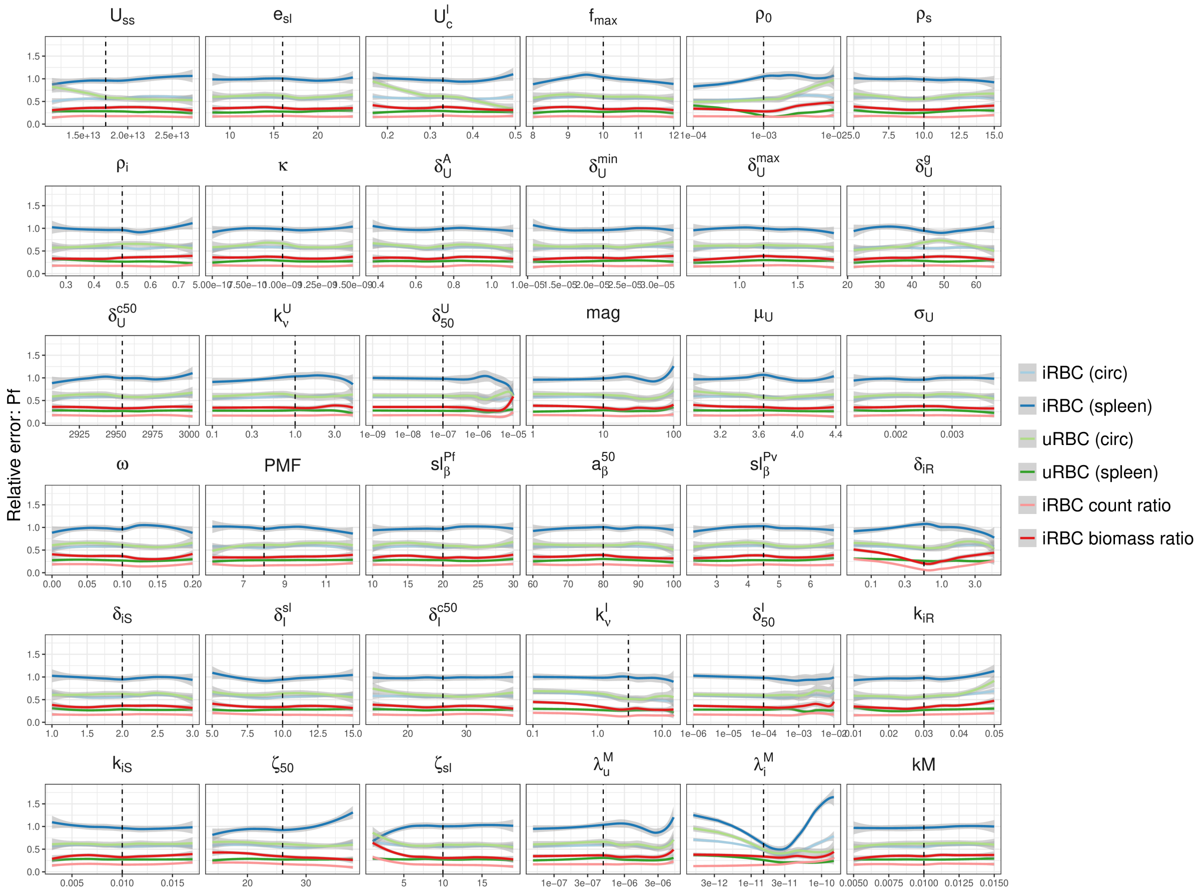

Figure E: Smoothed trend in relative error for each output variable, shown separately for each model parameter (Pf infections). Vertical dashed lines indicate our chosen baseline values.

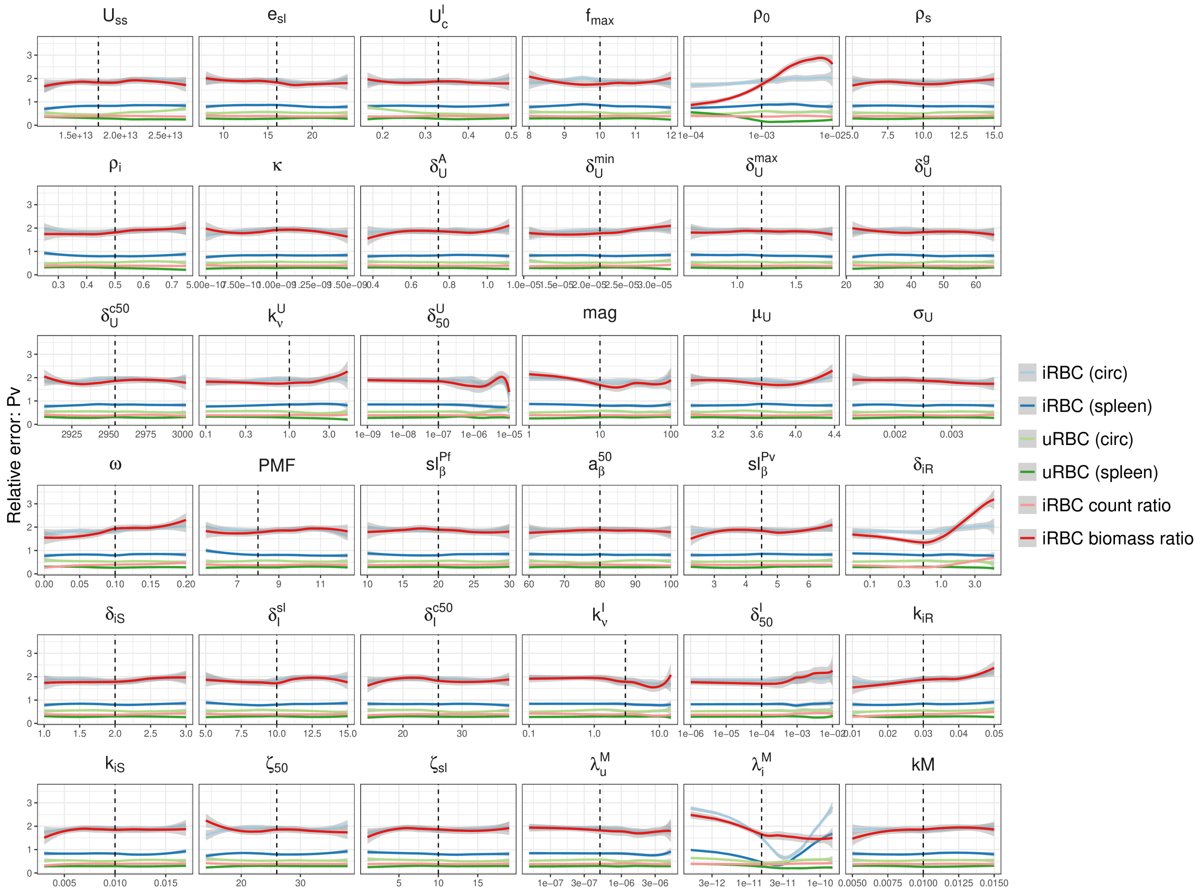

Figure F: Smoothed trend in relative error for each output variable, shown separately for each model parameter (Pv infections). Vertical dashed lines indicate our chosen baseline values.

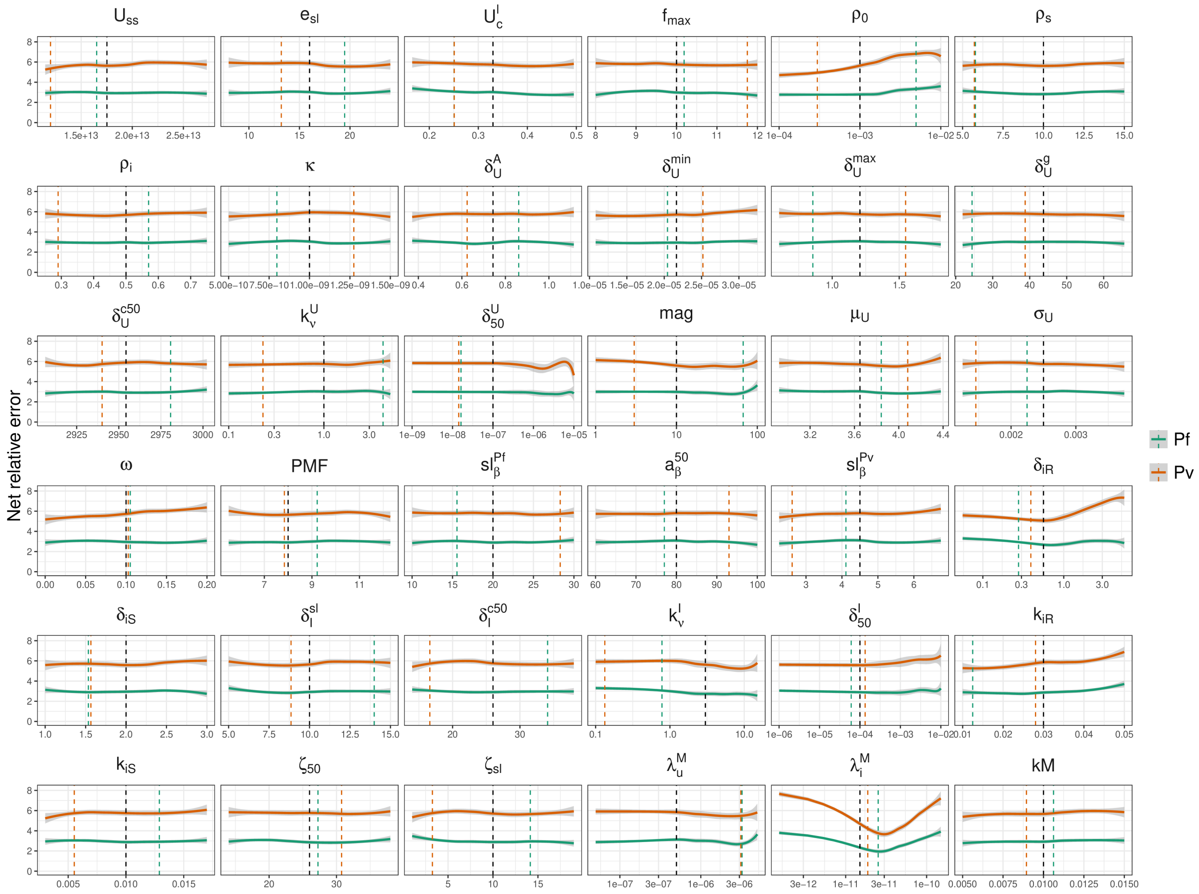

Figure G: Smoothed trend in net relative error, shown separately for each model parameter. Vertical dashed lines indicate our chosen baseline values (black) and the values for which the net error was minimal for each species (coloured).

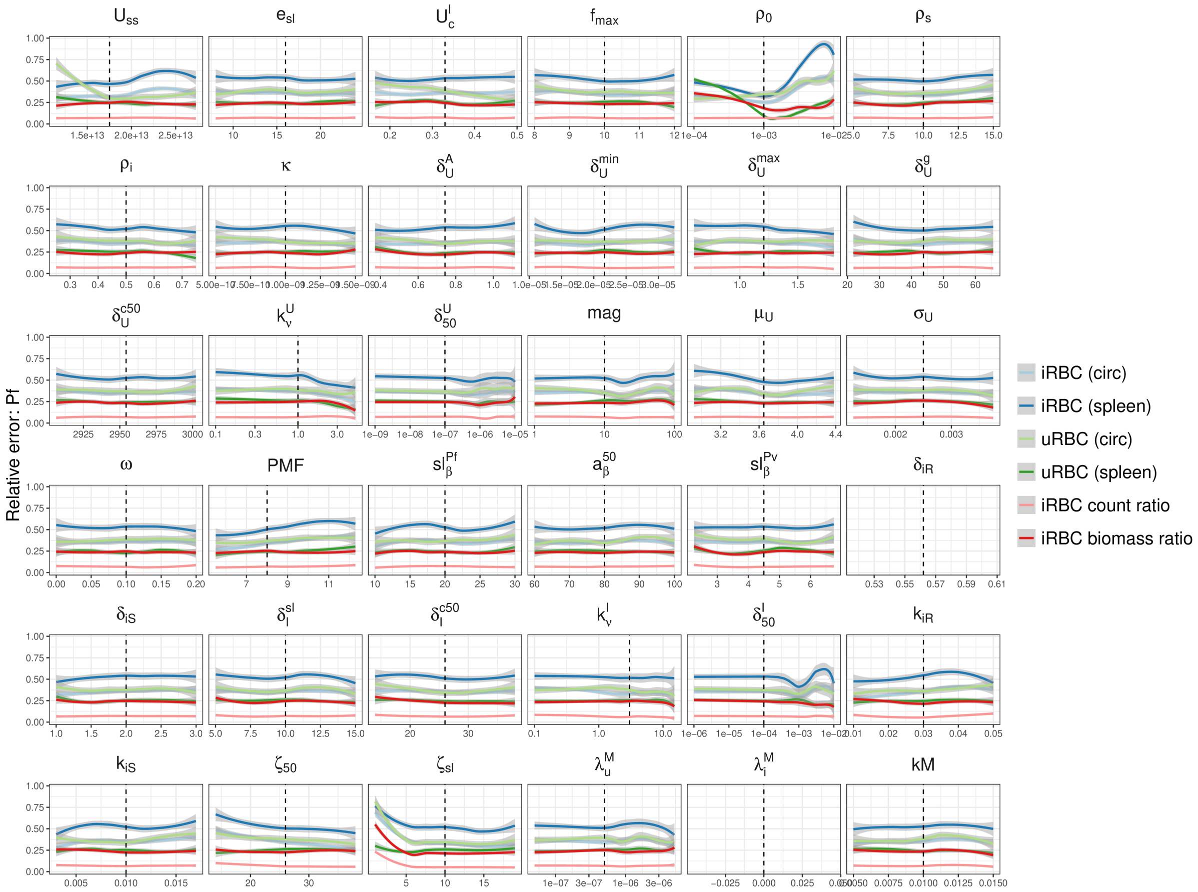

Figure H: Smoothed trend in relative error for each output variable, shown separately for each model parameter (Pf infections). Vertical dashed lines indicate our chosen baseline values.

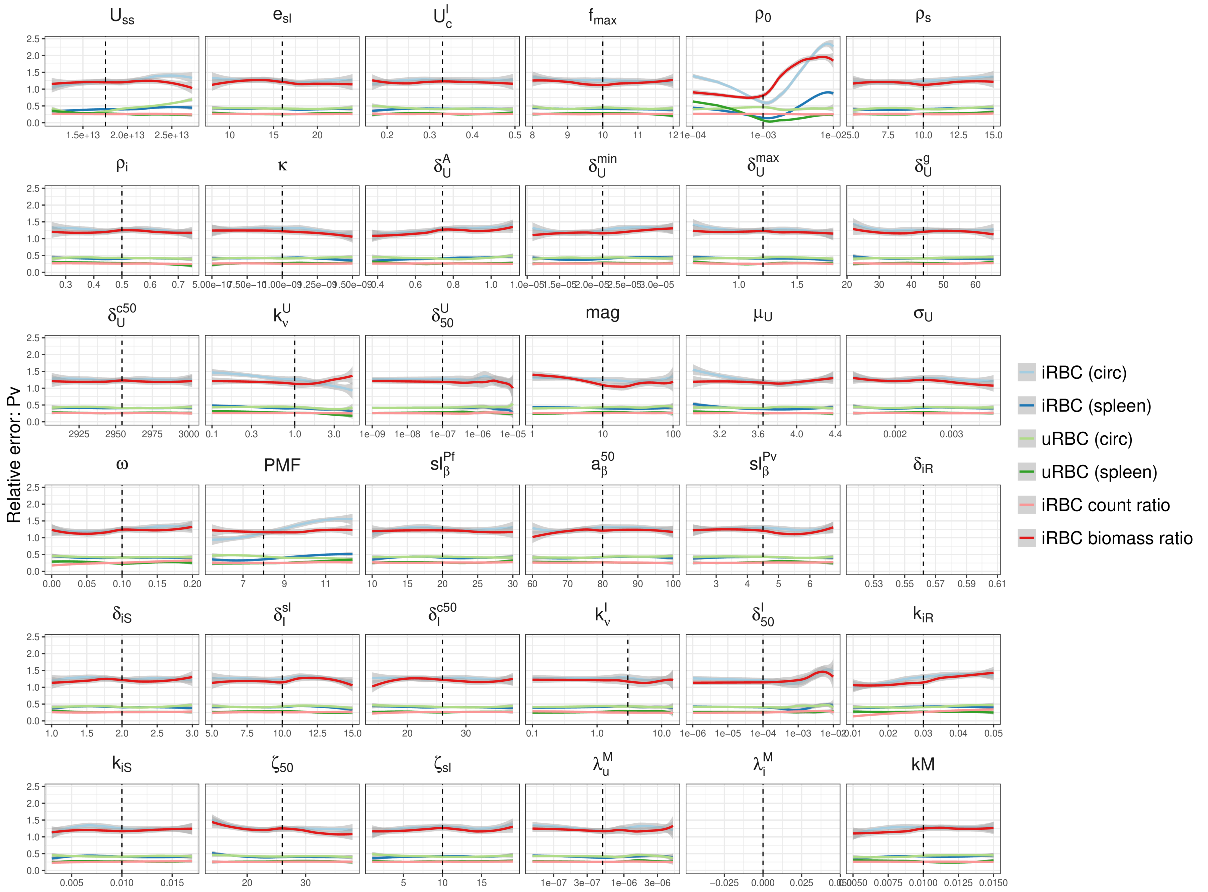

Figure I: Smoothed trend in relative error for each output variable, shown separately for each model parameter (Pv infections). Vertical dashed lines indicate our chosen baseline values.

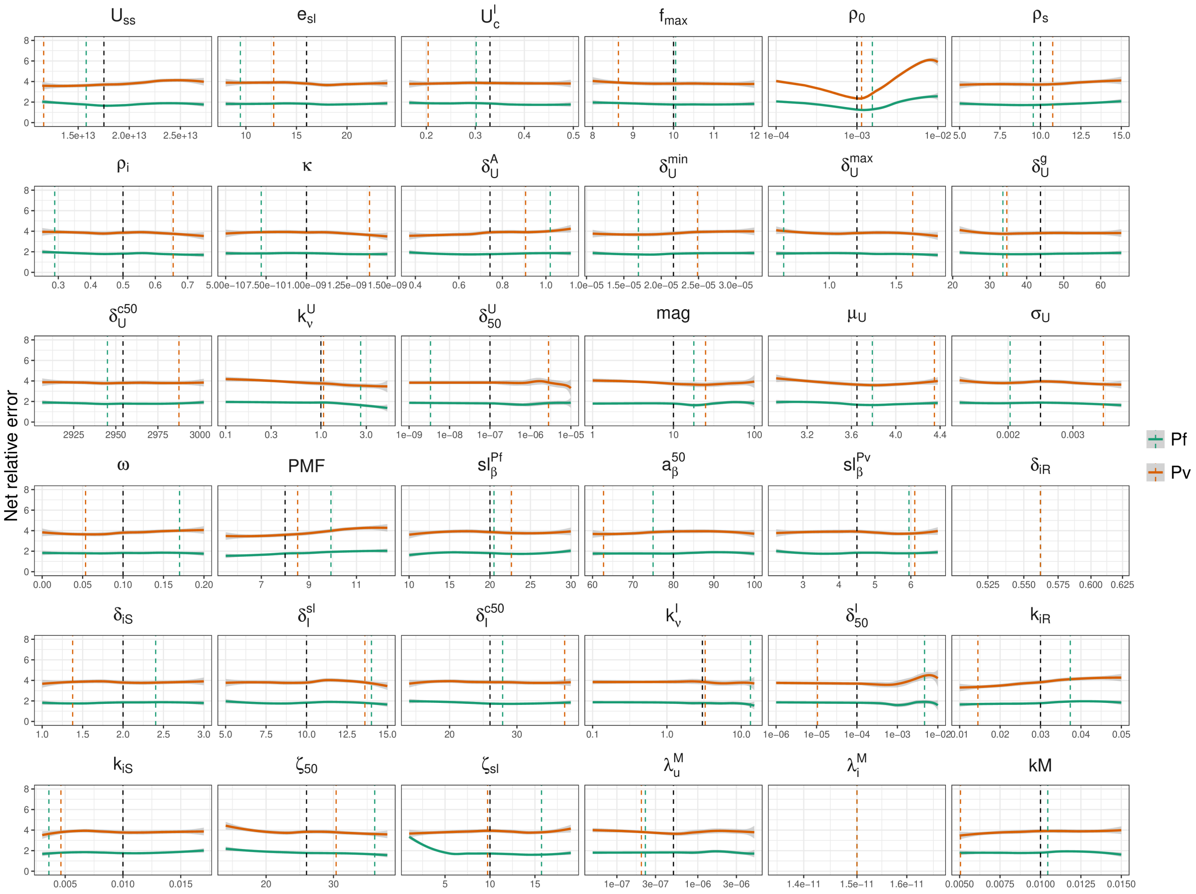

Figure J: Smoothed trend in net relative error, shown separately for each model parameter. Vertical dashed lines indicate our chosen baseline values (black) and the values for which the net error was minimal for each species (coloured).

ciated with significant risk of anaemia in Papua, Indonesia. PLOS ONE 11, e0165340. https://doi.org/10.1371/journal.pone.0165340

Simpson, J.A., Aarons, L., Collins, W.E., Jeffery, G.M., White, N.J., 2002. Population dynamics of untreated plasmodium falciparum malaria within the adult human host during the expansion phase of the infection. Parasitology 124, 247–263. https://doi.org/10.1017/s0031182001001202
